# How health systems facilitate patient-centered care and care coordination: a case series analysis to identify best practices

**DOI:** 10.1186/s12913-022-08623-w

**Published:** 2022-11-29

**Authors:** Kaitlyn Simpson, Wilson Nham, Josh Thariath, Hannah Schafer, Margaret Greenwood-Eriksen, Michael D. Fetters, David Serlin, Timothy Peterson, Mahshid Abir

**Affiliations:** 1 Acute Care Research Unit, Institute for Healthcare Policy and Innovation, University of Michigan, Ann Arbor, MI USA; 2 University of Michigan Medical School, University of Michigan, Ann Arbor, MI USA; 3 University of Michigan School of Public Health, University of Michigan, Ann Arbor, MI USA; 4 Department of Emergency Medicine, University of New Mexico, Albuquerque, New Mexico USA; 5 Michigan Mixed Methods Program, University of Michigan, Ann Arbor, MI USA; 6 Department of Family Medicine, Michigan Medicine, University of Michigan, Ann Arbor, MI USA; 7 Department of Emergency Medicine, Michigan Medicine, University of Michigan, Ann Arbor, MI USA; 8 RAND Corporation, Santa Monica, CA USA

**Keywords:** Patient centered care, Care coordination, Healthcare delivery, Health system transformation, Quality improvement, Case series analysis, Realist review

## Abstract

Large- and small-scale transformation of healthcare delivery toward improved patient experience through promotion of patient-centered and coordinated care continues to be at the forefront of health system efforts in the United States. As part of a Quality Improvement (QI) project at a large, midwestern health system, a case series of high-performing organizations was explored with the goal of identifying best practices in patient-centered care and/or care coordination (PCC/CC). Identification of best practices was done through rapid realist review of peer-reviewed literature supporting three PCC/CC interventions per case. Mechanisms responsible for successful intervention outcomes and associated institutional-level facilitators were evaluated, and cross-case analysis produced high-level focus items for health system leadership, including (1) institutional values surrounding PCC/CC, (2) optimization of IT infrastructure to enhance performance and communication, (3) pay structures and employment models that enhance accountability, and (4) organizing bodies to support implementation efforts. Health systems may use this review to gain insight into how institutional-level factors may facilitate small-scale PCC/CC behaviors, or to conduct similar assessments in their own QI projects. Based on our analysis, we recommend health systems seeking to improve PCC/CC at any level or scale to evaluate how IT infrastructure affects provider-provider and provider-patient communication, and the extent to which institutional prioritization of PCC/CC is manifest and held accountable in performance feedback, incentivization, and values shared among departments and settings. Ideally, this evaluation work should be performed and/or supported by cross-department organizing bodies specifically devoted to PCC/CC implementation work.

**Supplementary Information:**

The online version contains supplementary material available at 10.1186/s12913-022-08623-w.

## Background

Healthcare delivery innovation in the United States is focused on improving the “patient experience of care” as part of the triple aim proposed by the Institute for Health Care Improvement in 2007 [1]. These efforts can be attributed to the identification of patient-centered care (PCC) as a criterion of quality by the National Academies of sciences, engineering, and medicine (NASEM) [2] and the Affordable Care Act’s (ACA) support of innovations such as the patient centered medical home (PCMH) [3]. The NASEM’s definition of PCC includes “coordination and integration of care” recognizing the “special vulnerability that accompanies illness or injury” and concomitant dependence on providers to coordinate services and deliver timely information [2]. From the patient perspective, care coordination is integral to having their needs and preferences met, the failure of which is often perceived as “unreasonable levels of effort required on the part of themselves or their informal caregivers in order to meet care needs.” [4] therefore, despite care coordination (CC) and PCC often being seen as distinct healthcare delivery goals, PCC and/or CC (PCC/CC) are not mutually exclusive [5] and may be reasonably examined together.

Evidence-based PCC/CC interventions are a key tool for health system leadership seeking to improve PCC and CC, however the vast majority of PCC and CC efforts are explored through pilot, effectiveness, or mixed-methods studies at single institutions or within a single department. However, such studies are unable to provide evidence that adoption of the study intervention in a different health system would have similar outcomes [6].

Therefore, peer-reviewed studies exploring how various health systems facilitate PCC/CC at the institutional level may be particularly informative. The goals of the present study are to explore institutional-level facilitators of PCC/CC within multiple high-performing health systems and provide recommendations for health system leadership seeking to improve PCC/CC within their own institutions. Several recent studies examine institutional-level PCC/CC facilitators in US health systems through systematic review [7], single-case study [3], qualitative [8, 9], and mixed-methods [10] investigations. To date, peer-reviewed case series as well as realist reviews on this topic are limited. The present study adds a unique perspective to this body of work by comparing multiple cases, each individually analyzed using a realist review approach.

## Methods

### Study design and rationale

This study was conducted as part of a one-year project at a large, midwestern US health system seeking to improve PCC/CC. Sponsors for the project included executive leaders of the health system with expertise in health system management. These executive sponsors identified six US health systems with reputations for providing PCC/CC from which to identify best practices: Geisinger, Kaiser Permanente, Mayo Clinic, Cleveland Clinic, Allina Health, and Saint Joseph Mercy (Trinity Health System). The health systems they selected are widely acknowledged as high performing health systems across many parameters in the US, with the lattermost health system having special relevance as a neighboring institution. These health systems represent a diversity of models found in the US; for example, fee-for-service versus salaried payment structures, or varied acceptance of the wide range of health insurance plans available in US versus management and exclusive acceptance of their own health insurance plans.

We used a holistic multiple case study design with a revelatory rationale; in this way institutional-level facilitators of successful PCC/CC interventions could be explored in-depth and then compared to reveal patterns of similarity, and ultimately, generate insights previously inaccessible in the literature [11, 12]. Each case was developed using a rapid realist review [13, 14] of peer-reviewed literature. Realist methodology is primarily concerned with “how complex programs work in particular contexts or settings” in order to “enable decision-makers to reach a deeper understanding of the intervention and how it can be made to work most effectively.” [15] Unlike randomized control trials (RCTs) in which causality must be successive (i.e. if X, then Y), realist-informed causality is generative such that outcomes are the result of a special relationship – that of a specific mechanism or set of mechanisms working simultaneously within a specific context. Following this logic, realist review is well suited to delineating how the outcomes of PCC/CC inventions are influenced by mechanisms that necessarily operate within the context of their institution. In order to do this, the supporting literature for at least three successful PCC/CC interventions implemented per case institution were selected for analysis. Additionally, realist approaches may strengthen case studies or case series sensitivity to identify drivers of change in complex adaptive systems. Over the last 15 years, realist methodologies have been encouraged by the United Kingdom’s Medical Research Council to enhance health services research [6, 16]. In 2014, the formalized reporting guidelines for secondary realist evaluations was developed, from which this paper takes guidance [13] alongside elements of rapid realist review appropriate for the project from which this inquiry emerged, namely by focusing the research question and process to produce findings of practical relevance to a specific audience, rather than the development of a comprehensive theory [14].

### Search strategy and selection criteria

**Fig. 1:**
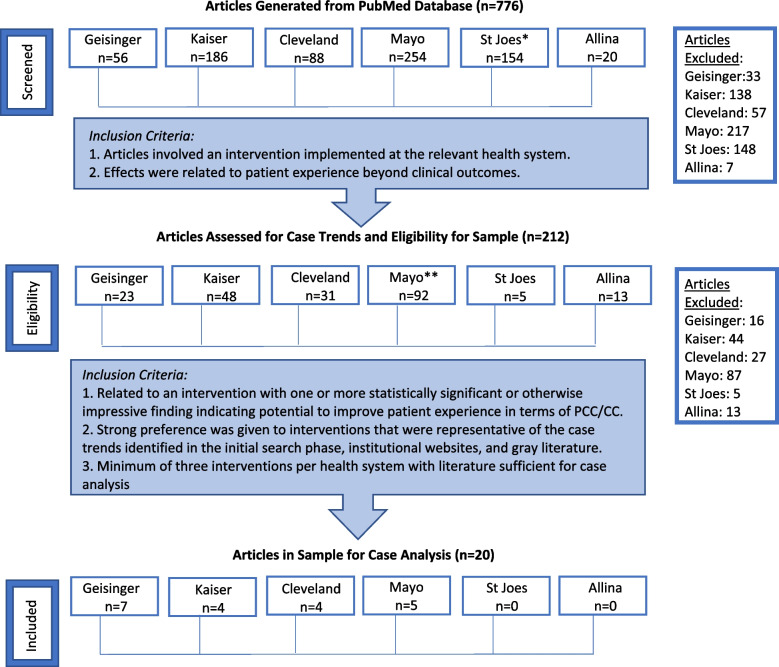
Adapted PRIMSA chart for peer-reviewed literature search and selection. PCC/CC = Patient-centered care and/or care coordination. *Broader search stream for affiliation only, due to zero hits when specified to PCC/CC. **54 additional articles regarding telehealth added from lists on institutional websites

Peer-reviewed literature supporting each case institution’s PCC/CC interventions was queried by one researcher in consultation with an information scientist in October of 2019 using the PubMed database. The following search was performed for each health system: “(Health System[Affiliation]) AND (“Patient-centered Care“ OR “Patient centered care” OR “Care coordination”).” If this yielded no results, a broader search by affiliation only was performed. One researcher screened peer-reviewed abstracts based on relevancy. Next, three researchers extracted information from articles deemed relevant from the screening into a matrix and assessed inclusion eligibility for the realist review sample. Two researchers at minimum read each article with one researcher reading every article and matrix entry to ensure consistency. Additionally, for each case, two researchers gathered gray literature queried via Google until saturation alongside detailed extraction of pertinent programs/initiatives on each health system’s institutional websites. Articles included in the review sample had to support successful interventions representative of each institution’s historical PCC/CC efforts, with literature rich enough for case analysis, i.e., detailed information about the intervention’s features, implementation, and institutional enviroment. An adapted PRISMA table with total and case-specific yields as well as inclusion criteria at each stage is depicted in Fig. 1. To be included in the final sample, an intervention had to have statistically significant or otherwise impressive findings demonstrating measurable positive impact on PCC/CC outcomes.

### Data extraction and synthesis

Case profiles for each health system were compiled using both the gray and peer-reviewed literature, as well as any pertinent literature identified in the review articles themselves [14]. Intervention data from the literature were compiled by four researchers into open-ended templates [14] to explore mechanism-outcome relationships via the following sections: (1) Background: *How did the intervention come into being? How was it designed? How was it implemented?*; (2) Intervention Details: *How does the intervention work? How do the “actors” interact with each other as a result of the intervention and/or its implementation*?; and (3) Facilitators: *What are explicitly or implicitly reported factors that helped with the success of the intervention*? Intervention impacts and outcomes were also compiled, including impacts not part of the a priori framework of the study. One researcher with close familiarity with each article checked other researchers’ templates to ensure accuracy and consistency. The case profiles were then independently analyzed by four researchers, at least two per case, for institutional-level PCC/CC facilitators, and then compared in cross-case analysis. To do this, researchers paid special attention to how the health system facilitated the mechanism-outcome relationships. Researchers met to reach consensus on key findings through discussion, with one researcher participating in all meetings for consistency. Any discrepancies were resolved through discussion.

## Results

First we describe our overall findings, then for each case we describe the institutional-level facilitators supporting their PCC/CC interventions, and lastly we describe the institutional-level facilitators identified in our cross-case analysis.

**Fig. 2:**
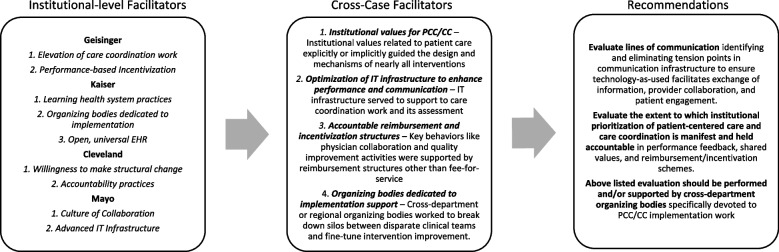
Key findings and recommendations

Our peer-review search yielded 20 articles related to 12 index interventions for 4 health systems: Geisinger, Kaiser, Cleveland Clinic and Mayo Clinic. Because Saint Joseph Mercy and Allina yielded research supporting less than three interventions, these cases were excluded. Table 1 provides key facts related to region, size, and population served for the included cases. Table 2 provides peer-review article information organized by case and intervention. The interventions reviewed represent a variety of strategies to improve PCC/CC related broadly to PCMH models, care transitions, provider communication, electronic health record (EHR) optimization, and teleservices. Interventions were implemented from the early 2000s to the late 2010s, with 83% having been implemented in the last 10 years. One intervention was not set up to report statistical significance [35] and another only reported limited statistically significant findings [26, 27]. They both otherwise report measurable positive effects that demonstrate potential of the intervention to improve PCC/CC. Table 3 provides the mechanism-outcome findings for each intervention identified through realist review. Figure 2 summarizes institutional-level facilitators by case, cross-case findings, and recommendations.

**Table 1:** Key health system facts for cases included in the final sample

Health System	Region	Facilities and size	Populations Served
Geisinger	Headquartered in Danville, Pennsylvania with facilities throughout Pennsylvania with affiliates in Delaware, Maine, and New Jersey.^a^	The organization includes 13 hospital campusesb and more than 84 primary and specialty care sites.^c^	1.4 million patients annually, including approximately 583,000 Geisinger health plan members.^d^
		30,609 employees,^d^ including 1800 physicians^b^ and more than 4500 nurses.^c^ Annually, these employees manage approximately 106,000 inpatient admissions and 6.4million clinic visits.^d^	Their service area covers approximately 45 counties with a population of more than 3 million.^e^
Kaiser^f,g^	Headquartered in Oakland, California with facilities throughout the Western United States with additional service areas of Hawaii, Georgia and the greater Washington, D.C. and Baltimore area.	39 hospitals and 706 medical offices.	Serves 12.2 million plan members.
		218,297 employees including approximately 23,000 physicians and 60,000 nurses.	
Cleveland Clinic^h,i^	Headquartered in Cleveland, Ohio, with facilities primarily in northeast Ohio and several smaller services areas in Southeast Florida and Nevada as well as international locations in Canada and Abu Dhabi	18 hospitals and 210 outpatient locations	Serves 2.4 million patients annually.
		67,554 employees, including 4520 physicians and scientists, and 14,458 nurses. 51,731 of these employees are based in Ohio.	Their primary service area includes 21 counties in northeast Ohio covering a regional population of approximately 4.4 million.
Mayo Clinic^j,k^	Headquartered in Rochester, Minnesota at their flagship campus, they also have large campuses in Arizona and Florida.	Operates at least 22 hospitals and 76 outpatient facilities.	Facilities serve 1.2 million patients annually.
	In addition, Mayo Clinic owns a regional health network called the “Mayo Clinic Health System” which directly operates or partners with facilities across Minnesota, Wisconsin, and Iowa.^l^	65,000 employees with 4800 are physicians and scientists. Their flagship campus accounts for more than half of these employees, staffing 36,330 with 2543 physicians.	Mayo Clinic considers its three campuses “destination medical centers”, reporting patients arriving from 138 countries.

^a^Our service area. (n.d.) Geisinger. Retrieved June 18, 2020 from https://www.geisinger.org/health-plan/about/service-areas

^b^Community Health Needs Assessment. (2018). Geisinger. Retrieved June 18, 2020 from https://www.geisinger.org/about-geisinger/in-our-community/chna

^c^Transforming Healthcare Through Continuous Innovation: 2014 System Report. (2014). Geisinger. Retrieved June 18, 2020 from https://www.geisinger.org/-/media/OneGeisinger/pdfs/ghs/about-geisinger/news-and-media/annual-reports/77219-1-2014SystemReport-Rev15-spreads.pdf?la=en

^d^Geisinger 2017 Annual Report: Changing the face of healthcare. (2017). Retrieved June 18, 2020 from https://www.geisinger.org/-/media/OneGeisinger/pdfs/ghs/about-geisinger/news-and-media/annual-reports/87179-Geisinger-AR_2017_final5_spreads.pdf?la=en

^e^For media: Fast facts. (n.d) Geisinger. Retrieved June 18, 2020 from https://www.geisinger.org/about-geisinger/news-and-media/for-media

^f^About: Fast facts. (n.d). Kaiser Permanente. Retrieved June 18, 2020 from https://about.kaiserpermanente.org/who-we-are/fast-facts

^g^2018 Annual Report. (2018). Kaiser Permanente. Retrieved June 18, 2020 from https://healthy.kaiserpermanente.org/static/health/annual_reports/kp_annualreport_2018/

^h^State of the Clinic 2019. (2019). Cleveland Clinic. Retrieved June 18, 2020 from https://my.clevelandclinic.org/-/scassets/files/org/about/who-we-are/state-of-the-clinic.ashx?la=en

^i^Governance & Leadership. (n.d.) Cleveland Clinic. Retrieved June 18, 2020 from https://my.clevelandclinic.org/about/overview/leadership

^j^Facts and highlights. (2011). Mayo Clinic. Retrieved June 18, 2020 from https://www.mayoclinic.org/documents/mc2045-pdf/doc-20078949

^k^An Inside Look at Mayo Clinic. (2019). Retrieved June 18, 2020 from https://mcforms.mayo.edu/mc7300-mc7399/mc7360.pdf and Fast facts

^l^2017 Report to Our Community. (2017). Mayo Clinic Health System. Retrieved June 18, 2020 from https://www.mayoclinichealthsystem.org/-/media/local-files/la-crosse/la-crosse-live-pdf-files/report-to-community-2017.pdf?la=en&rev=7f42ea5c795c4ee3af77be36b290f6de&hash=FF69E18843229B2092F3586F81B75067

**Table 2:** Peer-reviewed articles included in realist review sample, by health system case and intervention

Health System	Intervention Name and Description	Author, Year	Study Design	Key Findings or Data Reported to Support Intervention
Geisinger	“Proven Health Navigator (PHN)”	Gilfillan, 2010 [17]	Pre-experimental static comparison case control	• Admissions of PHN members were reduced 18% per year (p < .01) when compared to the expected outcomes if PHN had not been implemented • Readmissions of PHN members were reduced 36% per year (*p* = .02) when compared to the expected outcomes if PHN had not been implemented • Total cumulative spending was reduced 7% per year (*p* = .21; not significant) when compared to the expected outcomes if PHN had not been implemented
		Maeng, 2012 [18]	Pre-experimental static comparison	• For elderly patient exposed to PHN, the estimated odds ratio of experiencing diabetic amputation or reaching the status of end-stage renal disease significantly deceased – OR: 0.178 and 0.688 respectively (*p* < .01).
	Patient Centered Medical Home (PCMH) model of care designed to improve the quality, efficiency, and patient experience of care by blending chronic and primary care to offer an integrated form of management to meet all needs	Tomcavage, 2012 [19]	N/A	• Not an effectiveness study. Commentary and background information on PHN by nursing leadership.
		Maeng, 2013 [20]	Pre-experimental static comparison	• PHN respondents were twice as likely as non-PHN respondents to have noticed differences in their care (29.2% vs. 15.4%), care coordination (34.3% vs. 14.7%), and service (31.4% vs 14.6%).• PHN respondents were more likely to cite their primary care office as their usual source of care (83% vs. 68%) and were less likely to cite the emergency room (ER) as their usual source of care (11% vs. 23%).
		Maeng, 2015 [21]	Pre-experimental static comparison	• Over a 90-month period, total costs associated with patient centered medical home exposure declined by 7.9%• Largest source of saving was acute inpatient care ($34 or 19% savings per month per member)
Geisinger	“Geisinger Monitoring Program (GMP)”	Graham, 2012 [22]	Pre-post parallel Quasi experimental matched	• Admissions during GMP enrollment had a significantly lower readmission rate than admissions before or after GMP enrollment (10.1% vs.27.1 and 18.8%, *p* < 0.0001). • 44% reduction in 30-day readmissions in the study cohort compared to the matched control group (*p* = 0.0004),
	Readmission prevention telemonitoring program for Medicaid recipients using interactive voice response (IVR) technology.			
Geisinger	“Comprehensive Care Clinic (CCC)”	Maeng, 2017 [23]	Pre-post design, Pre-experimental. Cost focus.	• CCC enrollment associated with 78% reduction in acute hospital admissions (*p* = 0.053) and 60.3% reduction in ED visits (*p* = 0.017). • Estimated 28% reduction in per member per month total cost of care, primarily attributed to IP care reduction ($3931 observed vs. $5451 expected, *P* = 0.028) • Most significant source primarily attributed to IP care reduction (*P* = 0.028).
	Intensive primary care case management program for Adolescence and Young Adults with Special Care and Health Needs (AYASCHN)			
Kaiser	“5-Element Transitional Bundle”	Rice, 2016 [24]	Prospective Cohort	• Readmission rates decreased from 12.1% before the implementation of the intervention to 10.6% after the intervention was in place (2008 vs 2014) (*p* < .0001). • HCAHPS scores for the discharge instruction composite increased from 80 to 90% (*p* < .0001) moving from below the 50th to above the 90th national percentile. • Average time to the first post discharge appointment decreased from 9.7 days to 5.3 days (p < .0001)
	Standardized, multidisciplinary protocol for discharge, created and maintained based on patient-identified post-discharge needs			
Kaiser	“Nurse Knowledge Exchange *Plus*” (NKE*Plus*)”	Lin, 2015 [25]	Prospective Cohort	• Improvement in the mean HCAHPS score on the Nurse Knowledge Exchange Plus (NKEplus) nursing behavior bundle from 65.9 to 71.3% (p <. 001). (Behaviors reported were that the care board in room was updated with new caregiver’s names and patient plan, nurse reviewed patient’s daily care with the patient in a way they could understand, at change of shift the nurse caring for the patient introduced them to the new nurse, and that the nursing staff asked patient for input about daily care) • Improvement in the mean HCAHPS score for nurse communication from 73.1% before implementation to 76.4% after implementation (2010 vs 2014) (*p* <. 001).
	Model of nursing communication at shift change to reflect the principles of patient centered care			
Kaiser	“Clinical Pharmacy Call Center (CCPC)”	Stubbings, 2005 [26]	Review case study/report	• Mean cost avoidance was $324 per member per year • Patients treated by CPCC pharmacists for allergic rhinitis were more likely to receive a nasal corticosteroid prescription than control patients (90.7% vs.77.8%, *p* = 0.009)
	Telephone pharmacy service that integrates primary care, specialty care, nursing facilities and clinical pharmacy. CCPC clinical pharmacists provide care via telephone using established communication, documentation, and interaction protocols with patients and caregivers.	McGaw, 2007 [27]	Single Arm Pre-Post Analysis	• Patients transitioned by CPCC clinical pharmacists were 78% less likely to die (95% confidence interval (CI), 0.06–0.88); 29% less likely to need an ED visit(95% CI, 0.36–1.39); and 17% more likely to follow-up with primary care clinicians (95% CI, 0.99–1.37) than patients in the Usual Care group• Demonstrated an annualized per patient savings of $5276.
Cleveland	“Connected Care SNF”	Kim, 2017 [28]	Pre experimental static comparison Retrospective cohort	• Improvement in the 30-day readmission rates at the intervention SNFs (28.1 to 21.7%, P < 0.001) and slight increase in the 30-day readmission rates at the control SNFs (27.1 to 28.5%, *P* < 0.001). • Absolute reductions in 30-day readmission rates ranged from 4.6% reduction for patients at low risk for readmission and 9.1% reduction for patients at high risk. • Improvements in 30-day readmission rates were greater for medical patients (31.0 to 24.6%, p < 0.001) than surgical patients (22.4 to 17.7%, *p* < 0.001).
	Transitions of care model to prevent readmissions for patients discharged to SNF. “Connected Care team” of 2 geriatricians, 1 internist, 1 family physician and 5 APP provided care directly within participating SNFs 4-5 days per week.			
Cleveland	“R.E.D.E. (Relationship: Establishment, Development and Engagement) to Communicate: Foundations of Health Care Communication”	Windover, 2014 [29]	N/A	• Model explaining intervention
	Communication course for physician targeting relationship centered training.	Boissy, 2016 [30]	Observational study (qualitative/pre-post)	• Following the course, overall CGCAHPS scores for physician communication were higher for intervention physicians than for controls (92.09 vs. 91.09, *p* = 0.03).• Significant improvement in the post-course HCAHPS respect was seen in intervention versus control groups (91.08 vs. 88.79, *p* = 0.02)• Physicians also showed significant improvement in empathy (116.4 ± 12.7 vs. 124 ± 11.9, *p* < 0.001)
Cleveland	“Mobile Stroke Treatment Unit (MSTU)”	Taqui, 2017 [31]	Unmatched Quasi experimental	• Significant reduction of median alarm-to-CT scan completion times (33 minutes MSTU vs 56 minutes control group (traditional ambulance), *p* < 0.0001) • Significant reduction of median alarm-to-thrombolysis times (55.5 minutes MSTU vs 94 minutes control group (traditional ambulance), *p* < 0.0001) • Significant reduction of median door-to-thrombolysis times (31.5 minutes MSTU vs 58 minutes control group (traditional ambulance), *p* = 0.0012), and symptom-onset-to-thrombolysis times (97 minutes MSTU vs 122.5 minutes control group (traditional ambulance), *p* = 0.0485).
	EMS vehicle equipped with CT scanner allows for remote assessment and instruction by neuroradiologist and vascular neurologist			
Mayo	Teleneonatology service with hub and spoke model	Fang, 2016 [32]	Case study: measurements included reason for consult and a survey	• After completion of the telemedicine consultation, 27 neonates (32.1%) were able to remain at the referring hospital • User assessment of the technology revealed that audio and video quality were poor or unusable in 16 (25%) and 12 (18.8%) of cases, respectively. Providers failed to establish a video connection in 8 consults (9.5%). • 93.3% (*n* = 14 of 15) of surveyed local providers agreed that the telemedicine consult improved patient safety, quality of care, or both
		Fang, 2018 [33]	Pre-experimental: RCT [33]	• Greater resuscitation quality rating for the teleneonatology group vs control group (7 vs 4, median difference 1, (*P* = .002)• Newborns in the teleneonatology group were significantly more likely than their matched control to undergo measurement of temperature (79% vs 55%, *P* = .02), blood glucose (94% vs 81%, *p* = .03), and blood gas (49% vs 28%, *P* = .008).• When analyzing the matched pairs that had a consult within 1 h of birth, the positive impact of teleneonatology was greater (median resuscitation quality rating of 8 vs 4, median difference 2, *p* = .003)
		Fang, 2018 [34]	Qualitative Study [34]	• 94.6% of survey respondents agreed that teleneonatology was needed at their hospitals• 96.2% of respondents believed that teleneonatology consults were helpful.• 90.3% agreed that teleneonatology enhances communication between sites and 84.9% agreed that teleneonatology ensures standardization of care across sites
Mayo	Integrated, Colocated Specialist (ICS) model with neurologists embedded in primary care	Young, 2017 [35]	Pre-experimental - Pre-post design (with elements of a review)	• Reduced total face-to-face neurology visits per month by 25%, reduced referrals to tertiary neurology by 64% • 33% of curbside visits resulted in agreement with the Primary Care Physician and Provider (PCP) care plan without the need for additional diagnosis testing or face-to-face consults • Diagnostic testing or a face-to-face visit was estimated to have been avoided in 22% of patients if a curbside visit had been obtained earlier in the care planning process.
	Includes the integration of curbside, electronic and face to face consultations with the goal of enhancing care coordination and communication			
Mayo	“Mayo Expert Advisor (MEA)” Cardiovascular Risk Tool	Scheitel, 2017 [36]	Static comparison: with and without cardiovascular decision tool.	• Saved 3 minutes and 42 seconds in calculating the ASCVD score (*p* < 0.05) • Saved 3 minutes and 38 seconds in determining the recommendation (p < 0.05). • Improved accuracy from 60.61 to 100% for both the risk score calculation and guideline-consistent treatment recommendation.
	Informatics-based, EHR-integrated clinical decision support tool delivering patient-specific, automated cardiovascular risk scores and treatment recommendations

**Table 3:** Mechanism-outcome realist review findings, by health system case and intervention

Reported Intervention Outcome of Interest	Mechanism Findings
Geisinger	
**Intervention 1: ProvenHealthNavigator (PHN)** *Advanced patient-centered medical home (PCMH) model including embedded RN case managers specially trained in population health management and employed by the Geisinger Health Plan*.	
Significant reductions in both admissions (18% less) and readmissions (36% less) [17] with suggestion of dose-dependent response [21] in Medicaid populations as well as significant prevention of end stage chronic disease indicators for elderly patients [18].	Proactive identification of at-risk individuals by RN case managers using claims-based intelligence followed by review with PCP allowed for enhanced ability to proactively address acute exacerbations and chronic care needs, particularly in elderly populations [17, 18, 20, 21].
Significant improvements in patient perception of care: PHN patients twice as likely to report noticing differences in care, care coordination and service, and believe that the quality of care is difference and improved. Additionally, significantly 12.4% less like to report using the ED as a usual point of care [20].	RN case manager time was dedicated to care coordination – direct phone lines for patients, in-person development of care plans with highest-risk patients, and close follow up post discharge [17, 20].
**Intervention 2: Geisinger Monitoring Program (GMP)** *Post-discharge patients received automated phone calls that collected individualized information using EHR-integrated automated Interactive Voice Response (IVR) technology, with areas of concern or non-compliance requiring follow up pushed to the embedded PC RN case manager in real time* via *EHR. Four-week program; not intended to replace traditional contact.*	
Compared to case management alone without the IVR tool, there was a 44% intent-to-treat reduction in likelihood for readmissions, driven by second year of study (“incremental benefit”).	Efficient extension of case manager capacity; design based on failure of a manual readmissions prevention program of similar scale (~ 30 m in manual program vs 2-3 m in GMP).
Only 4% failed to participate in the full program compared to large drop-out rate seen in other telemonitoring program studies.	Attention to optimal balance of frequency of automated contact. Patients receive calls 1x per week and programming to retry calls at set intervals.
**Intervention 3: Comprehensive Care Clinic (CCC)** *Augmentation of PCMH model offering intensive primary care for Adolescents and Young Adults with Special Care and Health Needs (AYASCHN). For patients meeting complexity criteria, primary care provided by multidisciplinary care team including an internal medicine-pediatric physician, advanced practice practitioner, pharmacist, and embedded RN case manager.*	
Per member per month reduction of ~ 78% reduction for acute hospital admissions (*P* = 0.053) and ~ 60.3% in ED visits (*P* = 0.017)	Detailed patient/family self-management education surrounding exacerbations and how to respond provided, paired with availability of same day services, may have helped alleviate seeking emergency care unnecessarily
Per member per month total cost reduction of 28% ($3931 observed vs. $5451 expected; *P* = 0.028). Inpatient cost appears to drive the reduction as the most significant source (*P* = 0.028).	Care plans developed with family in joint appointments with RN case manager and dually trained internal medicine-pediatrics physician, as well as required laboratory, screening and other monitoring for medication optimization, may have helped to alleviate common gaps AYASCHN face when transitioning into regular adult primary care.
Kaiser Permanente	
**Intervention 1: “5-Element Transitional bundle”** *Multi-element standardized discharge protocol to reduce all-cause readmissions, designed using outcomes data and patient-identified post-discharge needs, with iterative improvements.*	
Significant decreases in readmissions rates up to 12.1% (P < 0.0001) and risk ratios from 0.72 to 0.66 (0.0001) moving from 75th to 90th percentile nationally.	Greater patient connection in-patient providers appears to be the major drivers of change, including the creation of a 24/7 “post-hospital hotline” with triage nurses able to page hospitalists as needed (required re-brokering of physician contracts to extend of hospitalist oversight for 48 hours post-discharge), and 48-hour follow up for all patients or 30-day case management for high risk patient by transition RNs.
HCAHPS discharge instruction scores moved from 50th percentile to 90th percentile nationally (*P* < 0.0001). Over an 8 year period mean time to first appointment post discharge reduced by 4.6 days (P < 0.0001)	Clear, discharge summaries with single post-hospital line listed to call for any reason and all follow up tests and appointments listed – key element in transitional bundle required ambulatory care appointments made prior to discharge made this feasible.
**Intervention 2: Nurse Knowledge Exchange** ***Plus*** **(NKE*Plus*)** *Set of minimum specifications and acronyms designed to improve nurse handoffs and patient engagement at shift change.*	
Mean HCAHPS score for 82 nursing units improved significantly by 3.3% (range 0.2-5.9% change per unit) over 4 years. Mean score for NKE*Plus* behaviors (based on four HCHAPS elements for RN-patient communication) for 60 units improved significantly by 4.7% (range 0.1-7.8% change per unit) over 4 years.	Nurses given protected time at shift change to conduct patient-engaged handoff at bedside via unit protocol changes (rounding on patient needs in the hour before shift change and non-nurse staff picking up extra responsibilities during shift change to minimize interruptions, as well as shift assignments limiting number of departing nurses incoming nurses heard from. Scale reflected at right supported by Innovation Consultancy which worked to ensure intervention was accepted, adapted, and sustained across many sites.
**Intervention 3: Clinical Pharmacy Call Center (CPCC)** *Phone-based telepharmacy program developed to address high demand to regional call center related to drug therapies. 20-24 FTE personnel serve 400,000 patients by working screening and reconciliation for new and/or recently discharged patients, fulfilling refill requests, and spreading information related to guidance or plan changes to patients. CPCC also helps develop and advise on clinical guidelines, acting as a support to clinical staff with drug-related questions.*	
Approximately ~ 10 K annual new patient transitions, 30-40% of which are estimated to be Medicare beneficiaries. Random chart review showed a cost avoidance of $324 per member per year, representing a cost avoidance benefit to KPCR members of $16.2 million dollars and an estimated $4.3 million in avoided primary clinic costs.	CPCC clinical pharmacists work alongside regional call center physicians to make medication and lab orders for new patients awaiting first PCP appointment. This frees up PCP time to focus on other care needs and reduces need for multiple initial appointments.
SNF patients transitioned to home via CPCC clinical pharmacists were 78% less likely to die (95% CI, 0.06–0.88), 29% less likely to need an ED visit (95% CI, 0.36–1.39) and 17% more likely to follow up with primary care (95% CI, 0.99–1.37) versus usual care, which meant transition care being provided by primary care clinics alone. CPCC detected at least one potential drug problem in 90% of discharge summaries [26].	Working with Kaiser’s Chronic Care Coordination (CCC) Department, CPCC provided medication review, reconciliation, and counselling for SNF patients post-discharge in their home; researchers comment this potentially lower-stress setting may have allowed for increased retention versus clinical setting. Additionally, pharmacist to specifically instruct SNF patients or caregivers to bring the medications physically in front of them, which may not be realistic in clinical settings. Effects mediated by CCC coordinators who receive CPCC notes, provide additional services, and make recommendations to primary care providers [26].
Cleveland Clinic	
**Intervention 1: Connected Care SNF** *Cleveland Clinic physicians and advanced practice professionals visited patients discharged to SNFs 4-5 times per week and w/i 48 hours provided a comprehensive assessment and record review*	
Participating SNFs saw significant 6.8% absolute reduction in all-cause 30 day admissions versus usual care. Particular benefit to high-risk and medical (versus surgical) patients.	Meaningful, frequent face-to-face physician engagement with SNF patients not typically receiving continuing medical care after discharge to SNF. Emphasis on goals of care and palliative care specialist as part of connected care team ensure appropriate expectations and prevention of inappropriate readmissions.
**Intervention 2: R.E.D.E. to Communicate: Foundations of Health Care Communication** *Relational skills course designed by and implemented in Cleveland Clinic. System-wide, physician targeted 8-hour block of training with small and large group skills practice.*	
For physicians who took part in the R.E.D.E. communication skills training, significant improvement to CGCAHPS measuring patient experience scores were higher than for controls (92.09 vs. 91.09, *p* < 0.03). Significant improvement in the post-course HCAHPS “Respect” domain means versus control groups (91.08 vs. 88.79 respectively, *p* = 0.02) [30].	Main mechanism appears to be improving physician self-efficacy and sense of worth. Significant improvements to empathy and burnout among physicians were seen alongside patient experience improvements, even after 3 months following the session, and pre-post surveys showing significant satisfaction with course [30]. R.E.D.E strategies being integrated directly into the medical interview likely reduced physician burden to find individual way to incorporate relational skills to patient interaction indirectly or as an additional point of care [29, 30].
**Intervention 3: Mobile Stroke Treatment Unit (MSTU)** *MSTU staffed with RN, paramedic, EMT and crossed trained EMT-CT technologist. Vehicle is outfitted with CT scanner with output to neuroradiologist, point-of-care lab equipment, and A/V equipment for real-time instruction from Cleveland Clinic vascular neurologist (VN)*	
MSTU program patients received thrombolysis significantly sooner than control patients in two main ways: 38.5 minutes sooner from the alarm time and 26.5 minutes sooner from door. 25% of patient who received thrombolysis did so within recommended 60 minutes of symptom onset.	Dramatically and significantly sooner diagnostic service (CT scan) drove faster treatment times. Patient arrives to hospital and MSTU within similar timeframe, but researchers comment hospital environment brings delays to care delivery. Coordination with city EMS agencies for simultaneous dispatch ensured mechanism achieves greatest potential.
Mayo Clinic	
**Intervention 1: Teleneonatology program** *Mayo Clinic provides telemedicine consults to six community hospitals (level I and level II nurseries) throughout the region using A/V live-feedback instruction for high risk deliveries and/or newborn resuscitation. Goals include improve outcomes for neonates and reduce the need to transfer infants up 40-120mi away depending on site.*	
Neonates receiving teleneonatal consults were significantly more likely to undergo measurement of temperature, blood glucose and blood gas, and significantly more likely to undergo all three. In matched pairs analyses by blinded expert panels, telemedicine neonates were significantly more like to have been impacted positively within 1 h of birth and/or to have been provided with higher quality resuscitation [33].	Mixed method study into barriers and facilitators [34] revealed that reliable and easy to use IT infrastructure was important for this time-sensitive, live instruction intensive telemedicine intervention. Mayo’s Center for Connected Care worked closely to analyze, troubleshoot, and enhance IT capabilities and improve processes – such as switching from wireless to wired connections and allowing neonatologist control of camera angles [32].
**Intervention 2: Integrated, Colocated Specialist (ICS) - Neurology** *Neurologist in co-located in primary care clinic at 0.6 FTE with partial allocation of 3 RNs and 3 MAs. The ICS-Neurology model goal was to increase collaboration with PCPs including physicians and APP. Curbside and electronic consultations were encouraged, and effect on face-to-face consultation with ICS-neurology, diagnostics and tertiary referral were measured.*	
Referrals for face-to-face neurology visits for patients reduced by 25%; Referrals to tertiary neurology reduced by 64%.	Collaboration was encouraged through alteration of the EHR such that the ordering of face-to-face neurology consultations were not permitted unless a curbside consultation (informal via phone or email) had been performed and recorded by PCP. As a result of this process feature, 33% of curbside consultations resulted in agreement with PCP plan and avoided the need for face-to-face consultation entirely.
Nearly one quarter of curbside consults resulted in escalation for the patient to be seen same day or sooner than next available appointment.	Encouragement of curbside consultation paired with the ICS-neurologist having approximately 50% unscheduled time meant that patients with urgent neurological symptoms identified via PCP- ICS Neurologist collaboration were able to access care sooner.
**Intervention 3: MayoExpertAdvisor (MEA) Cardiovascular Risk Tool** *EHR-integrated clinical decision support tool for cardiovascular risk assessment and improved cholesterol management. Study provides simulation analysis to see effects on accuracy and time management among specialists, PCP and APP in routine care settings.*	
Clinicians who did not use MEA spent an average of 5 minutes and 8 seconds to determine the ASCVD score and determine a recommendation for patient care. With MEA, the clinicians spent a total of 1 minute and 31 seconds to calculate the ASCVD score and determine the recommendation, a reduced of 3 minutes and 38 sections. Chart review at the primary care practice indicates this equates to a time savings of 3 hours and 45 minutes per day, a half day of clinician time.	MEA saves clinician time by consolidating relevant information into one place. With MEA, providers did not have to move through and between multiple tabs to find relevant information. Further, MEA is not a separate application but conveniently integrated in the EHR via Mayo-develop EHR viewer called “Synthesis” that retrieves and presents data to clinicians in a more intuitive, easy-to-navigate format.
Clinicians without MEA had a 60.61% accuracy of ASCVD risk score calculation and 60.61% accuracy in selecting the guideline recommended treatment and there were significant differences in time to complete both tasks between physicians, NPs, Pas, and other clinical types. With MEA, clinicians had 100% accuracy in both and the time difference between providers normalized.	Mayo has put considerable resources into their IT infrastructure towards care standardization, ultimately allowing for the technology to support this and other similar interventions. Natural language processing (NLP) allowed auto-pulling from EHR is promoting ASCVD risk score accuracy – researchers comment that the most frequent errors that clinicians made in were related to not identifying the most recent data and inputting age, gender and smoking status incorrectly. Further, Mayo’s extensive development of clinical decision support drawing on Mayo physician-guided knowledge base [32] eliminated variation in recommendation within and between provider groups.

### Institutional-level facilitators supporting Geisinger interventions

#### Elevation of care coordination work

As part of a strategic goal of innovation, Geisinger brought population health management directly into clinical care teams through the role of a case manager. This work was previously performed by external Geisinger Health Plan (GHP) offices [17, 18]. The case manager role became a critical to many of Geisinger’s PCC/CC interventions [17–23]. Clinical integration of population health management was supported through ensuring case managers had no competing care priorities [19] and creating information technology (IT) infrastructure for predictive risk modeling [17, 18], performance reports [17, 18], EHR-integrated longitudinal care planning [23], and provider alerts [20, 37].

#### Performance-based Incentivization

Goals of interventions were linked to performance categories like improvements in patient satisfaction and care quality [18, 37] that Geisinger providers were incentivized to strive towards; this incentivization accounted for up to 20% of total compensation per physician [37].

### Institutional-level facilitators supporting Kaiser Permanente interventions

#### Learning health system practices

Learning health system practices supported the evolution of each intervention. For example, design for one intervention was directed by bottom-up assessment of patient need, and post-implementation performance was used to make improvements [24]. In another invention, 2000 hours of Plan-Do-Study-Act (PDSA) cycles were utilized to optimize intervention performance in the piloting stage [25]. Lastly, another intervention centering around a pharmacist call center arose after regular program auditing found long response times at nurse call centers frequently utilized for medication questions [26].

#### Organizing bodies dedicated to implementation

The interventions took place on a regional scale for maximal impact; this could not have been done without organizing bodies to support implementation efforts. Examples include “breaking down silos between settings and stakeholder groups” [24] and achieving buy-in and sustainability in 15 units across 14 medical centers despite initial unpopularity [25].

#### Open EHR

Kaiser’s EHR provided universal, standardized access across providers and settings essential to intervention features. In two interventions, pharmacists [24, 26, 27] had full access to patient records to enhance their medication reconciliation, while the success and design of a third intervention, a handoff protocol, depended on the EHR’s universality such that “an RN on any unit in any medical center could float or transfer to another unit or medical center and be proficient” [25].

### Institutional-level facilitators supporting Cleveland Clinic interventions

#### Willingness to make structural change

Dramatic structural changes to accommodate patient needs guided two interventions [28, 31]. In both interventions, Cleveland Clinic multidisciplinary care teams were brought from Cleveland Clinic facilities to outside facilities to directly care for patients transitioning in and out of their institution’s care. Cleveland Clinic’s willingness to make these changes was considerable, demonstrated by working intensively with outside partners as well as significant resource investment. Similar willingness on the part of the organization to expend resources was observed through the incorporation of a mandated in-person, institution-wide relational skills course for physicians [29, 30], though no specific structural change accompanied this intervention.

#### Accountability practices

At Cleveland Clinic salary renewal is linked to performance, and extensive outcomes data is published annually, motivating provider commitment to improve the patient experience and/or outcomes [38]. The potential effect of this was particularly notable in one intervention, where Cleveland providers were held accountable to the success of the intervention through outcome-specific performance assessment [28]. Additionally, for a different intervention, researchers comment that success was in part due to their salary model, recognizing it would have otherwise been difficult to motivate staff participation [30].

### Institutional-level facilitators supporting Mayo Clinic interventions

#### Culture of collaboration

Mayo’s explicit care philosophy prioritizes “union of force” wherein “personnel work collaboratively in teams within and across all departments to meet the […] needs of patients” [39]. Per the care philosophy, collaboration is reinforced by a salary model. The provider-provider collaboration one intervention formalized was directly related to these care goals, with researchers commenting that such collaboration is likely more effective under a salary model versus a fee-for-service model with no reimbursement for such collaboration [35].

#### Advanced IT infrastructure

Using technology to free up provider time [35, 36] and to connect providers across settings [32–34] was essential to the interventions, and appears to be supported by considerable investment in an EHR-integrated knowledge management system (described by Shellum, et al.) [40] and the supportive work of the Center for Connected Care established in 2015, which has helped implement at least 54 telemedicine initiatives [41].

### Cross-case analysis

While each case presented unique institutional level-factors, there were factors in common which may be considered thematically as follows:

#### Institutional values for PCC/CC

Institutional values related to patient care explicitly or implicitly guided the design and mechanisms of nearly all interventions. Geisinger’s strategic goal of innovation, and Mayo’s care philosophy serve as particularly clearly stated and manifested value expressions.

#### Optimization of IT infrastructure to enhance performance and communication

IT infrastructure served to support care coordination work and its assessment; for Geisinger and Mayo in particular, a redesign of the IT infrastructure was the basis for increased efficiency and performance. However, the extent to which the technology achieved its goal was influenced by its effect on the quality of patient-provider or provider-provider communication. For example, in one of Geisinger’s interventions, automated patient calls were explicitly *not* intended to replace traditional patient contact; rather, they helped case managers know when to initiate a conversation [19].

#### Accountable reimbursement and incentivization structures

Key behaviors like physician collaboration and quality improvement activities were supported by reimbursement structures other than fee-for-service. This was an especially notable facilitator at Cleveland Clinic, where performance linked salary models served to motivate providers towards positive PCC/CC outcomes. Notably Geisinger and Kaiser both have integrated models – these not only support high-value behaviors at the provider level but also facilitate the ability to pilot PCC/CC interventions in the first place. Geisinger is uniquely positioned to leverage its health plan to develop the commercial market towards value-based care, since a third of its patients are both financially and clinically served by Geisinger entities [20].

#### Organizing bodies dedicated to implementation support

Cross-department or regional organizing bodies such as Mayo Clinic’s Center for Connected Care and various Kaiser departments were found to support intervention implementation. These organizations worked to break down silos between disparate clinical teams and fine-tune intervention improvement.

## Discussion

This research illustrates how four highly successful health systems have facilitated patient-centered care and care coordination and can be used to inform initiatives in other health systems across the U.S. seeking to improve PCC/CC. Four key areas of focus resulted from our case analyses; in brief: (1) *Institutional values for PCC/CC*, (2) *Optimization of IT infrastructure to enhance performance and communication*, (3) *Accountable reimbursement and incentivization structures*, and (4) *Organizing bodies dedicated to implementation support.*

Based on these findings, health systems seeking to improve PCC/CC should consider taking the following steps. First, **evaluate lines of communication** between patients and providers and between providers, identifying and eliminating tension points and/or bottlenecks in communication infrastructure to ensure technology-as-used facilitates efficient and effective exchange of information, provider collaboration, and patient engagement. Second, **evaluate the extent to which institutional prioritization of patient-centered care and care coordination is manifest and held accountable** in performance feedback and values shared among departments and settings. This should be considered alongside how reimbursement structures may play a role in provider incentivization to meet care quality and patient experience goals. Finally, this **evaluation work should be performed and/or supported by cross-department organizing bodies** specifically dedicated to PCC/CC implementation work. Without such an organizing body, PCC/CC efforts may remain siloed within departments and/or clinical teams and may not be sustained, improved upon, and disseminated throughout the institution after initial pilots. Dedicated organizing bodies may be especially important because there is limited peer-reviewed research evaluating broad health system dynamics such as communication and institutional prioritization; such bodies might allow health systems to evaluate their own institutions on a continual basis, develop institution-specific best practices, and remain dynamic and responsive to change as institutional needs, patient expectations and technology evolve.

The available literature examining institutional-level PCC/CC facilitators in US health systems supports some of the findings and recommendations in the present study. For example, a 2013 primary qualitative case study of one health organization in Washington State found that to act upon on their major motivators of institutional-level change, moving away from an “unsustainable” fee-for-service model and continuously learning from past performance would be essential [3]. This is similar to one of the present study’s key institutional-level facilitators: using learning health system practices to optimize PCC/CC change within the context of an integrated payment model. Interestingly, the need for health systems to learn from themselves support the idea that even if there were robust institutional-level research on how to facilitate PCC/CC, health system leaders would still be required to look internally to identify what will bring about more PCC/CC in their own contexts and is suggestive of the need for organizational bodies to regularly support such work. Two other primary qualitative studies of reputable PCC health care organizations echoed reimbursement, leadership incentivization, and learning health systems as key PCC drivers [8, 9]. Despite general agreement regarding the importance of these factors, a 2015 study into highly ranked HCAHPS hospitals found that while nearly all of them employed some form of data feedback to drive improvement, only 57% offered incentives for high performance [10].

Interestingly, none of these previous studies commented on lines of communication and their relationship to technology and IT infrastructure, a theme highlighted in the present study, suggesting there may be benefits in using an approach informed by realist methodology. The case study is valuable because it offers a narrative of how various institutional practices interplay to affect PCC/CC change. In the case of this review, using realist informed methodologies to examine the mechanisms supporting 12 PCC/CC interventions produced unique findings related to communication. Further, this study used multiple cases to compare such narratives to arrive at more generalized best practices.

This study has limitations. First, the study is focused solely on US health systems which operate in a unique context that may not apply to health systems in other countries. Another limitation is the top-down case selection by executive sponsors. This limitation is somewhat mitigated by the expertise of the executive sponsors selecting the cases, who chose health systems with widely acknowledged reputations for PCC/CC efforts. Future researchers may rectify this by systematically identifying cases through using publicly available survey and rankings information for organizations of interest. Further, there was no outreach to leaders of the case study institutions to verify or qualify our findings. All findings were based on retrospective analysis of existing literature. Future studies may consider follow-up with researchers and/or health system stakeholders to verify findings, and/or conduct primary qualitative research.

Future research into large-scale and/or organizational-level change to encourage PCC/CC should consider case series with realist methodology as a potentially effective method to identify best practices. Given that many of the interventions identified in this case series may require significant financial and labor investment, a critical focus of future PCC/CC intervention research should include cost-effectiveness analyses to help health systems decide whether to pursue study outcomes. Related areas of research that should be explored include whether contractual inclusion of protected time for QI and/or research activities for health care professionals encourages PCC/CC on the institutional level, as well as investigations of challenges faced by institutions seeking to implement such PCC/CC-related interventions.

## Conclusion

This study indicates that health systems seeking to promote PCC/CC should consider creation of institutional-level organizing bodies dedicated to such work. Key areas of focus for such bodies should include evaluating how IT infrastructure can be leveraged to improve communication, and the extent to which institutional prioritization of PCC/CC is manifest and held accountable in performance feedback, incentivization, and values shared among departments and settings.

## Supplementary Information

**Additional file 1.**

## Data Availability

All data generated or analysed during this study are included in this published article or its supplement.
