# Supplementary material for: How health systems facilitate patient-centered care and care coordination: a case series analysis to identify best practices
Source: BMC Health Serv Res. 2022 Nov 29;22:1448. doi: 10.1186/s12913-022-08623-w (PMC9710067; doi:10.1186/s12913-022-08623-w)
Supplement: Supplementary file 1 — Additional file 1. [file 12913_2022_8623_MOESM1_ESM.docx]

**Case Studies Supplement to “How health systems facilitate patient-centered care and care coordination: A case series analysis to identify best practices”**

This supplement contains the detailed case studies pertaining to this case series analysis. Each case study includes an institutional profile with information related to institutional trends in patient-centered care and care coordination using gray and peer review literature. Additionally, three interventions representative of these trends were selected for analysis. Information pertaining to these interventions was extracted from supporting peer review literature, including the background of the intervention (how did the intervention come into being?), the details of the intervention (how does the intervention work?), the intervention’s outcomes and impacts (including impacts not part of the a priori framework of the study) and, lastly, what facilitated the success of the interventions. These case studies informed the study’s mechanism-outcome and cross-case findings (Table 3 and Figure 2 respectively) arrived at through discussion with research team.

1. Geisinger Institutional Profile Page 2
   1. Intervention 1 - “Proven Health Navigator (PHN)” Page 4
   2. Intervention 2 - “Geisinger Monitoring Program (GMP)” Page 12
   3. Intervention 3 - “Comprehensive Care Clinic (CCC)” Page 16
2. Kaiser Institutional Profile Page 19
   1. Intervention 1 - “5-Element Transitional Bundle” Page 20
   2. Intervention 2 - “Nurse Knowledge Exchange Plus” (NKE*Plus*)” Page 23
   3. Intervention 3 - “Clinical Pharmacy Call Center (CCPC)” Page 26
3. Cleveland Clinic Institutional Profile Page 29
   1. Intervention 1 - “Connected Care SNF” Page 31
   2. Intervention 2 - “R.E.D.E. (Relationship: Establishment, Development and Engagement) to Communicate: Foundations of Health Care Communication” Page 34
   3. Intervention 3 - “Mobile Stroke Treatment Unit (MSTU)” Page 37
4. Mayo Clinic Institutional Profile Page 41
   1. Intervention 1 - Teleneonatology service Page 43
   2. Intervention 2 - Integrated, Colocated Specialist (ICS) model Page 47
   3. Intervention 3 - “Mayo Expert Advisor (MEA)” Page 51
5. Supplement References Page 55

Case Study 1. Geisinger

**Institutional Profile**

***Institutional Purpose*^1^** *–* “Everything we do is about caring — for our patients, our members, our Geisinger family of physicians and employees, and our communities.”

***Institutional Values*^1^** *–* “At Geisinger we value: Kindness – We strive to treat everyone as we would hope to be treated ourselves; Excellence – We treasure colleagues who humbly strive for excellence; Learning – We share our knowledge with the best and brightest to better prepare the caregivers of tomorrow; Innovation – We constantly seek new and better ways to care for our patients, our members, our communities and the nation.”

***Key Facts*** *–* Geisinger is an integrated, multi-state health, open system and nonprofit health plan that serves approximately 1.4M patients annually, including approximately 583,000 Geisinger health plan members.^1^ Geisinger is headquartered in Danville, Pennsylvania and has facilities throughout Pennsylvania, primarily rural central and northeast Pennsylvania, with affiliates in Delaware, Maine, and New Jersey.^2^ In total their service area covers approximately 45 counties with a population of more than 3 million.^3^ The organization includes 13 hospital campuses^4^ and more than 84 primary and specialty care sites.^5^ In 2017 they reported an operating revenue of $6.3 billion, with 30,609 employees,^1^ including 1,800 physicians^4^ and more than 4,500 nurses.^5^ Annually, these employees manage approximately 106,000 inpatient admissions and 6.4M clinic visits.^1^

***Characteristics^6^*** *–* Geisinger provides and facilitates care through a multitude of operating units: Geisinger Health Plan, each individual hospital, system-wide clinical service lines which make up the multidisciplinary group practice (“Geisinger Clinic”), Geisinger Community Health Services (GCMS), and central support functions which include innovation and quality teams. Each of the clinical service lines are co-led by an MD and an administrator, and the central support teams work closely with operational leaders to meet shared performance-incentive goals. GCMS was founded as a complement to the health plan and manages a portfolio of healthcare services including hospice, home infusion and social services. Each of these operating units is responsible for achieving their own financial and quality targets.

Geisinger has a uniquely open yet integrated system thanks to the interaction between their facilities, human capital and health plan. The main medical center operating in Danville is the only facility wholly staffed with Geisinger providers – all other hospitals are an open mix. The Geisinger group practices covers both GHP enrollees and non-GHP consumers, with two thirds of GHP revenue being brought in from non-GHP payers. Additionally, more than 60% of GHP enrollees are serviced by non-Geisinger providers through contracts with more than 15,000 independent physician and 80 community hospitals, and there is a subset of Geisinger providers who are active in non-Geisinger hospitals. This unique model has allowed Geisinger to pilot innovation programs with the third of their patients who are both financially and clinically served by Geisinger entities and then leverage the health plan to develop the commercial market towards value-based care. Major programs in patient navigation and care process standardization, detailed in the next section, have resulted from Geisinger’s institutional prioritization of innovation, a strategic goal set in 2005. In support of these programs, Geisinger developed its IT infrastructure to support predictive analytics and a range of telehealth services.^7^ Geisinger also participates in the Care Connectivity Consortium health information exchange program with other leading health systems and is leading the Keystone Health Information Exchange to share medical information with a wide range of facility types throughout Pennsylvania.^7^

***Trends in Patient Centered Care and Care Coordination*** *–* Nearly all of the peer-reviewed literature captured in our review of the health system’s patient-centered and care coordination intervention center around three major programs that have resulted from the Geisinger’s 2005 strategic innovation goal: 1. ProvenCare,^8,9^ 2. ProvenNavigator,^10,11,12,13,14,15^ and 3. The MyCode Community Health Initiative.^16,17^ ProvenCare is a collection of programs developed since 2006 for a variety of chronic and acute conditions. Each program contains a set of care processes, performance goals and financial incentives linked to meeting the service-line goals. The programs are dependent on the EHR for provider-facing tools, like alerts and reminders, and to facilitate tracking of performance metrics. The creation of the ProvenCare programs share a methodology that involves multidisciplinary groups coming together to identify best practices and decide how to integrate these practices into the Geisinger workflow. ProvenNavigator was developed in parallel, essentially taking the role of population health management out of a centralized CHP office and embedding it into each primary care site though an RN case manager.^6^ Most recently, MyCode was developed as a “patient centered precision health” initiative that brings the power of individualized care through genomics to an entire community free of charge. This is enabled through a research infrastructure in which study participants may opt-in to have a free genetic screening on the condition that their genetic information is stored in the MyCode biorepository and linked to their EHR for the dual purpose of discovery research and to improve their clinical care.^16^ Geisinger has built several integrated research and clinical care intuitions to support this initiative. Additionally, an investigation of Geisinger’s websites revealed programs relevant to patient-centered care and care coordination including: a “ProvenExperience” refund program for care that did not meet patient expectations;^18^ a “Fresh Food Farmacy” food-as-medicine program aimed to improve diabetes outcomes by providing meals for qualifying households with positive results;^19^ talent and hiring tools specifically geared towards cultivating and retaining customer-service oriented employees;^20^ a number of initiatives supported by the Steele Institute for Innovation including a behavioral insights team to inform care processes and a care delivery program for pregnant women suffering from opioid used disorder;^21^ community-based family and pantry services integrated into Geisinger facilities;^22^ and technology for patients to visualize wait times and reserve spots for urgent care facilities^23^

***Comment on Interventions Selected for Geisinger*** *–* Interventions selected include (1) a PCMH-embedded RN case manager and (2) a telemonitoring program, both using risk stratification, as well as (3) an intensive pediatric primary care model. These interventions were selected after review of 23 peer-review articles featuring studies taking place at Geisinger facilities that were published by or affiliated with Geisinger personnel.

**Intervention 1. Proven Health Navigator^11,12,14^ – Comprehensive Case Management as Part of PCMH Model**

***Intervention Background***

The US “health care industry is facing increasingly complex challenges such as new regulatory requirements, value-based purchasing, an aging population, increased complexity of care delivery, and heightened focus on consumer-directed care. Although industry responses have been multifaceted, there is a widespread agreement on the need to strengthen the primary care foundation of the health system by reorganizing the way in which primary care is delivered.”

Interest in the “medical home” model became popularized as a solution to a fragmented US health care delivery system in the 2000s. This model has been associated with higher quality care and improved patient experience. There is special potential of this model to improve care for chronic conditions and yield savings to the health care system through the prevention of acute care. In the mid-2000s Geisinger health system and Geisinger Health Plan developed a medical home model called “Proven Health Navigator.” This model was originally “introduced for Medicare Advantage enrollees in 11 practices owned by Geisinger Health System (GHS) in Pennsylvania”, through primary care practices. The PHN model builds upon the National Committee for Quality Assurance (NCQA) Physician Practice Connections and Patient-Centered Medical Home (PPC-PCMH) standards with a focus on enhancing the “self-management and team-based” approach through additional focus on disease and case management. Notably, PHN was designed prior to the release of the NCQA PCC-PCMH standards and met or exceeded all standards once they were available to compare. Researchers comment that where the standards exceed this is “because our goal was to impact the quality, patient experience, and efficiency of care across the full continuum of care, not just in the office of the primary care physician (PCP),” therefore “we believed that additional components and activities were necessary.”

Geisinger researchers comment that impact of advanced patient centered medical homes (PCMHs), such as PHN, with respect to quality and coordination of care were relatively limited into the early 2010s. Furthermore, the actual patient experience of such programs was also limited. It was only in October 2011 that “the Agency for Healthcare Research and Quality (AHRQ) released CAHPS (Consumer Assessment of Healthcare Providers and Systems) Patient-Centered Medical Home Item Set, which is a survey tool specifically designed to capture patient experience of care in clinics that have been transformed into PCMH.” Geisinger then explored both the impact in quality and in-patient experience to gauge the success of PHN in a series of literature published from 2010-2015, represented by the three studies discussed in this section.

“Designing this unique care model required an integrated approach that included a cooperative partnership between Geisinger’s Community Practice Service Line and the organization’s health insurance arm (Geisinger Health Plan, or GHP), with each entity focusing on its core area of strength— from population management to development and implementation of clinical best practices. This partnered approach built a new model founded on an alliance between an insurance company, patients and their families, primary care physicians (PCPs), and other health care partners. The pilot was conducted at 2 primary care practices in November of 2006.” “Two years later the Navigator was expanded to include the health system’s broader adult commercial population.” The PHN continued to expand and “by 2011, there were 43 PHN sites, which included 36 Geisinger-owned primary care practices, as well as 7 contracted primary care practices in GHP’s provider network”

***Intervention Details***

- Broad overview of system-level implementation: “The ProvenHealth Navigator was rolled out in phases over a seven-year period from late 2006 through mid-2013. Phase 1, involving three primary care clinics, started in November 2006. Phase 2, involving ten additional primary care sites, started a year later. By June 2013 there were eight phases, expanding to include a total of eighty-six Navigator sites located throughout central Pennsylvania.”
- “In October 2006 and January 2007, the PHN model was introduced into 2 pilot GHS practice sites selected because of their large GHP Medicare Advantage population and because their locations made them easily accessible for our PHN management team… During 2007 and January of 2008, the PHN model was expanded to include the Medicare Advantage members in 9 additional practices”
  - “Implementation was focused on the GHP Medicare Advantage population because the high prevalence of chronic illnesses and the resource use of this population provide the best opportunity to demonstrate and evaluate the impact of the interventions.”
- “Almost half of its membership receives primary care from Geisinger-owned primary care clinics, all of which had been converted to PHN sites by 2011. The other half of the membership receives primary care from GHP’s contracted network of PCPs.”
  - “In general, relative to the Geisinger-owned primary care clinics, there is wider variation among the contracted primary care sites in terms of physician leadership; systems of care for comprehensive chronic and preventive disease management; cultural evolution to physician-directed, team-delivered care; implementation of electronic health records; integration with other health care providers in the community; and other aspects of care management practices that contribute to care experiences”
- “Prior to implementation at each site, all practice staff were trained on the core components of the model”
- “The PHN model has 5 functional program components:”
  - (1) Patient-Centered Primary Care Team Practice defined by: “provider-led, team-delivered care;” “patient and family engagement;” “enhanced access and scope of services;” and “HIT optimized preventive and chronic care.”
  - (2) “Integrated Population Management”
    - “Central to the model is the transfer of population management capabilities, including nurse case managers, from the health plan [Geisinger Health Plan (GHP)] to the practice sites.”
      - “First, many of GHP’s population management activities were moved to the practice site. Geisinger Health Plan provided case managers for each practice at a ratio of 1 nurse for every 800 Medicare patients to serve as the hub for population-based activities.”
      - “The GHP-embedded case managers were integrated as part of the practice care team. They were provided with utilization and predictive modeling reports derived from GHP claims data. For the first time, these reports gave the practice teams a systematic way to identify relative risk for their GHP patients.”
      - “Within the PHN sites… nurse case managers are physically embedded within the practices to provide even more personalized and coordinated care by building long-term relationships with those at-risk patients.”
    - “Population segmentation and risk stratification”
    - “Case management for complex, comorbid conditions”
  - (3) Micro-delivery Systems, also called “Medical Neighborhood”
  - (4) Quality Outcomes Program – PHN dictates that “performance reports documenting the quality, utilization, and overall cost-of-care results were provided to the practice” and that “quality outcomes were aligned with preexisting preventive and chronic disease care quality initiatives.”
  - (5) “Value Reimbursement System”, also called “Value-based reimbursement model”
    - PHN added “added a shared savings incentive model to the GHP reimbursement arrangement.” These were based on “improvement in bundled metrics for [the preexisting preventive and chronic disease care quality initiatives] and other agreed-upon metrics.”
    - “Financially, while the Navigator sites continue to receive fee-for-service payments from GHP, the total reimbursement is linked to their performance via bonus payments and a shared savings program based on documented metrics of quality and utilization. These metrics include widely accepted measures such as the Healthcare Effectiveness Data and Information Set and the Consumer Assessment of Healthcare Providers and Systems”
- PHN “aims to provide complete patient-focused care and seeks ultimately to achieve the following goals:” (1) optimization of health outcomes of every patient; (2) delivery of evidence-based care in a patient-centered care model; (3) delivery of ‘‘value’’ in health care as evidenced by improved quality, patient experience, and efficiency; (4) improvement in the viability of primary care; and (5) not only bridging the gaps among different ‘‘silos’’ in the health care system, but seeking to optimize the flow of patients through all of the various silos.
- Tiered implementation and stratification of services based on patient risk
  - “Initially, the PHN teams focused on improving the management of the highest risk patients.”
  - “As progress was made, expanded strategies focused on members at moderate and low risk.”
    - “Patients with gaps in preventive or chronic care were identified by EHR registries and health plan claims tools. Health plan nurses with training in disease management targeted moderate-risk members with hypertension, coronary artery disease, and diabetes for self-management education; worked with providers to ensure appropriate screenings; and assisted in optimizing medications.”
    - “Site-based practice staff reached out to low-risk members to coordinate preventive care screenings such as mammograms, colorectal screening, and influenza vaccinations.”
- More details on the role of the GHP integrated care managers:
  - In the PCP setting, case managers “met with the highest risk patients to design patient-specific care plans.”
  - Case managers had dedicated phone lines in order for high risk patients to call them directly.
  - Case managers “provided close follow-up for patients transitioning from hospital to home. This activity focused on reaching out to the patient within 48 hours of discharge, medication reconciliation, appropriate resources and social supports in the home, and timely follow-up with the patient’s PCP.”
  - “The case managers also formed partnerships with preferred home health agencies and nursing homes. Outreach and education regarding the PHN strategy were provided to these agencies”
  - “These embedded case managers, for instance, receive lists of high-risk patients from GHP, and they review these lists together with the primary care provider at their respective sites. The case manager, therefore, takes the clinic’s knowledge of the patients and couples it with the claims-based intelligence (that is, predictive models and risk stratification software based on claims data) in order to target those most in need of intervention with the most intensive services”
- “Monthly team meetings that included PCPs, office staff, case managers, and GHP staff were held to evaluate results, discuss practice workflow and care access, and review hospital admissions for missed opportunities.”
- PHN “explicitly calls for the PCPs to develop systems of care for their patients when they are seen by other physicians or in other settings.”
  - “Under the Navigator model, each patient-centered medical home designs a care system that identifies acting physicians at other care sites and increases communication and coordination between them and the medical home.”
- “Pharmacy management initiatives were developed to improve generic utilization, assist members approaching the Medicare Part D coverage gap, and provide members with acute care protocols for treating exacerbations of chronic conditions.”
- “Additional financial support was provided by GHP to pay for new services in the PCP office.”
  - “An example is dedicated phone lines to allow high-risk patients to contact their case managers directly.”
- “Geisinger Health System has an electronic health record (EHR) implemented systemwide for all ambulatory and inpatient care. This EHR also is used by GHP case managers and patients. These EHR capabilities were operational in all participating practices for several years prior to the launch of the PHN. All Geisinger-owned primary care practices, including the PHN sites, participated in a preexisting, EHR-enabled quality initiative to improve preventive, diabetes, and coronary artery disease care.”
- The EHR plays a central role in the model’s care processes including: “tracking and registry capabilities for several chronic diseases are embedded” in to the EHR; “electronic prescribing as well as test and referral tracking also are available in the EHR”; and “advanced communication capabilities for patients and providers are available through the electronic portals portion of the EHR system.”

***Intervention Impacts and Outcomes***

From a study focus on for the Medicaid population at GHP sites:^14^

- “At baseline (2005), there were no statistically significant differences in sex or HCC scores of patients treated in the 11 PHN sites compared with the propensity score–matched non-GHS practices (Table 3). Patients treated in the intervention sites were approximately 6 months younger on average than those treated in comparison sites (P <.001), but because all results were regression adjusted, any potential bias associated with this age difference should have been eliminated. Average monthly admissions and readmissions per 1000 patients also were similar between the 2 groups (P = .24 and P =.74, respectively). Average spending per member per month was approximately 4% higher in the intervention cohort than in the comparison cohort (P = .04).”
- Based on actual versus expected analysis:
  - The PHN model was associated with a total cumulative reduction of 56 admissions per 1000 members per year (18%; 95% CI, −30% to −5%; P<0.01).
  - The PHN model also was associated with a cumulative effect of 21 fewer readmissions per 1000 members per year (−36%; 95% CI, −55% to −3%; P = .02).
- “The regression model estimated that the PHN model reduced cumulative total spending by 7%, but this difference did not reach significance (95% CI, −18% to 5%; P = .21).”
- “Results were qualitatively similar if all non-GHS clinic sites, rather than the propensity-matched comparison group, were used as a control cohort (data not shown).”
- “… based on its own actuarial analysis, GHP found that the PHN practices did generate savings and triggered incentive payments under the quality-based shared savings incentive system” (but not on per member per month spending formally in the study).
- “There are concerns that improved care coordination may increase the cost of prescription drugs, thereby decreasing or eliminating medical services savings. However, a separate analysis of the changes in drug expense over time for both groups of practices demonstrated no differential impact or erosion of savings in the PHN sites.”

From a study focused on survey evaluation of patient experience:^12^

- “To evaluate the impact of PHN on patient experience of care, the authors conducted a survey of patients whose primary care clinics had been transformed to ‘‘PHN sites’’ and were under case management at the time of the survey. A comparable survey of patients from non-PHN sites also was conducted for comparison.”
  - “To measure patient experience of care, an original survey instrument was developed. Ideally, an existing, validated survey instrument (such as CAHPS PCMH items, which had not been released by the time of this study) would have been preferred and used… The survey focused on collecting information that was not already available from GHP’s member profile database.”
  - “Based on the survey data, patient experience was assessed in terms of the following 4 domains of patient care experience: perceived changes in care delivery, usual source of care, access to care, and PCP performance.”
  - Inclusion criteria emphasized case-managed patients likely to interact frequently (and therefore adequately for the study) with the health system primarily defined by a set of chronic conditions that would predict frequent contact.
- “Patients in PHN sites were significantly more likely to report positive changes in their care experience and quality.”
  - “PHN respondents were roughly twice as likely as non-PHN respondents to have noticed differences in their care, care coordination, and service. They also were more likely to report that the quality of care at their primary clinic site is different and has improved.”
- PHN Patients “were more likely to cite the physician’s office as their usual source of care rather than the emergency room (83% vs. 68% for physician’s office; 11% vs. 23% for emergency room).”
- “There was no significant difference between PHN and non-PHN patients in their perceptions of access to care or primary care physician performance in terms of patient-centered care (e.g., listening, explaining, involving patients in decision making).”
  - Researchers note a “lack of a significant difference in reported access to care and patient perception of PCP performance. Specifically, the PHN and non-PHN respondents had similar assessments of their PCPs’ interaction with them, including listening to their concerns, explaining issues, and involving patients in the decision-making process, among others. Also, there were no significant differences in terms of respondents’ reported ability to access their primary care clinics and specialists, or the availability of test results within a 1-week period.”
- From a study focus on cost and utilization analysis:^11^
  - “We estimated cost savings associated with Geisinger Health System’s patient-centered medical home clinics by examining longitudinal clinic-level claims data from elderly Medicare patients attending the clinics over a ninety-month period (2006 through the first half of 2013). We also used these data to deconstruct savings into its main components (inpatient, outpatient, professional, and prescription drugs). During this period, total costs associated with patient-centered medical home exposure declined by approximately 7.9 percent.”
    - “There was, on average, $53 savings in the per member per month total cost of care per site (in regression-adjusted 2006 dollars). This translates to about 7.9 percent total cost savings, on average, across the ninety-month period.”
  - “…the largest source of this savings was acute inpatient care ($34, or 19 percent savings per member per month), which accounts for about 64 percent of the total estimated savings.”
    - “Other cost components also show some cost savings, but these estimates are not statistically significant.”
  - Does dependent effect on admissions: “longer exposure was also associated with lower acute inpatient admission rates.”
    - “The exhibits suggest that longer Navigator exposure is associated with a greater magnitude of cost savings, and this pattern is consistent with what we observed in terms of the association between acute inpatient admission rates and Navigator exposure.”
  - “The results of this study suggest that patient-centered medical homes can lead to sustainable, long-term improvements in patient health outcomes and the cost of care.”
  - “Moreover, as Exhibit 2 indicates, there is no evidence of “cost shifting”— that is, the cost savings in one area of care (in this case, acute inpatient care) did not lead to increased costs in other areas of care. Savings were observed in all four cost components but were statistically significant only for acute inpatient costs.”
  - “Obviously, such cost savings will not be sustained indefinitely. At some point, an incremental Navigator exposure will start to yield smaller returns (that is, the law of diminishing marginal returns) and eventually yield no additional savings. Our data show, however, that any diminishing return to additional Navigator exposures still had not been observed almost eight years since the initial Navigator conversion. This finding has an important implication for the sustainability of PCMH models in achieving lasting cost savings in larger contexts.”

***Intervention Facilitators***

- “We hypothesize that the comparative success of the PHN model was partly due to its ability to leverage existing physician–patient and inter-provider relationships to fundamentally change the way care is delivered rather than work outside the system to improve care.”
  - Background for this comment: “Introduction of a medical home care delivery model was associated with a significant reduction in hospital admissions and readmissions for a population of Medicare Advantage enrollees. Our findings present a contrast to the recently published results of the Medicare Health Support demonstration, a set of parallel, randomized controlled trials of traditional disease management delivered by third parties to disease-specific populations. Despite targeting sicker individuals, participating programs had little effect on healthcare utilization or spending.”
- “Our findings, coupled with qualitative observations… highlight the importance of placing nurse case managers directly into the practices and arming them with data and analytical capabilities.”
- “In addition, proactive identification of at-risk individuals provides an opportunity to use patient-specific action plans to implement timely interventions for acute exacerbations of chronic illnesses.”
- PHN “differs from many other medical home efforts that may not include robust case management programs, attention to care delivered outside of the PCP office, shared savings reimbursement, or direct health plan support.”
- “PHN model is situated in an integrated payer–provider environment (i.e., the payer and provider are part of the same corporate entity) with long-standing use of an ambulatory EHR, in a Medicare population with high baseline spending and relatively little patient turnover. These factors almost surely contributed to PHN’s success and may therefore limit generalizability to other settings.”
  - “The PHN model, however, has subsequently been introduced into non-GHS practices. Moreover, implementation experience to date suggests that the key components of the PHN model are on-site case management, the use of population data, and the shared savings incentives, all of which could be implemented outside of an integrated delivery model.”
- “Another possible explanation for the lack of significant differences between PHN and non-PHN groups in terms of access to care and PCP performance is that there already may be an existing trusted relationship between patients and their PCPs that persisted post PHN intervention. It may be that when patients and their providers already have an ongoing long-term relationship, a PCMH transformation such as PHN strengthens and formalizes this relationship, which may not be manifested in terms of any detectable differences in the standard measures of access and PCP performance, as demonstrated in this study. If this explanation is correct, it argues for transforming existing practices into PCMHs (as was the case for PHN) to leverage the existing patient provider relationships rather than moving patients from non-PCMH practices to new PCMH practices where no such relationship exists.”
- Regarding tiered implementation:
  - “One crucial advantage of this “phased” Navigator rollout was that it allowed for variation in the length of Navigator exposure across the sites. That is, while some sites remained non-Navigator (that is, Navigator exposure of zero), selected others became Navigator sites at different times, allowing for internal comparisons across the primary care clinics that eventually became Navigator sites.”
- Researchers identify “three main reasons” for the “success” of the program in terms of its cost savings and avoidance of admissions:
  - “First, it is truly a data-driven payer provider partnership that goes beyond simply enhancing information technology infrastructure at practice sites and seeks to translate practice-specific data into meaningful care plans by clinical experts.”
    - “For example, GHP hires, trains, and manages the embedded case managers, partly because practices often lack resources to support such capabilities.”
    - “This is in contrast with other clinic-based case management models in which additional case management duties are simply added on top of the existing workload of nurses who often lack training and resources.”
  - “Second, it is led by systemwide programmatic leadership that focuses on the entire care process instead of a single point in the process.”
    - “… strategies have been implemented within the Navigator to redesign the workflow that would support a comprehensive and coordinated approach to managing such patients in the clinic setting.”
  - “Third, it seeks to extend value for patients and medical professionals beyond traditional primary care settings.”
    - “Another example is optimizing treatment settings for patients with certain conditions (for example, heart failure, pneumonia, and atrial fibrillation) who are often treated in inpatient settings but can also be effectively and safely treated in outpatient clinics. Although not an explicitly stated feature of the patient-centered medical home in general, this is consistent with the medical home’s overall aim to improve health care value by revitalizing primary care”
- “GHP membership accounts for only a subset of the total patient population treated by the primary care practices included in this study. This also implies that the generalizability of our findings is unclear. We note, however, that the magnitude of the cost savings reported here is similar to the estimated PCMH cost savings reported in a study by Michael Paustian and colleagues (7.7 percent lower per member per month adult cost), which suggests that our findings are not unique and are potentially replicable. On the other hand, another study by Robert Reid and colleagues reported lower savings of approximately $10 or 2 percent per person per month, even though it included only twenty-one months of post-PCMH implementation data. This suggests that there is likely to be significant variability in the patient-centered medical home’s ability to achieve cost savings depending on geographical and institutional contexts.”

**Intervention 2. Telemonitoring programs using Interactive Voice Response (IVR) to reduce readmissions^24^**

***Intervention Background***

Readmissions within a 30-day period of discharge contribute to a large number of Medicare hospitalization. General sources thought to contribute to high readmissions include lack of communication with patient and between providers after discharge and failure to see patient in outpatient setting after discharge. The 2010 ACA directed CMS to track these types of hospitalizations and implement payment penalties based on low performance. Numerous interventions to improve readmission rates were developed in response. One intervention Geisinger Health System chose to implement to reduce readmissions is an interactive voice response (IVR) protocol to facilitate transitions in care after discharge. This IVR protocol, in which patient answers to automated questions on phone calls, was layered on top of a pre-existing comprehensive case management model, the “ProvenHealth Navigator” launched in 2006, six years prior to the study period. (This comprehensive case management model is explored in detail in intervention 1, above).

Of note, prior to this IVR protocol, the Geisinger Health Plan (GHP) had previously developed a monitoring program involving manual calls to the patient by Health Plan clerical staff incorporating a series of 8 or 9 questions depending on the patient’s reason for hospitalization, with certain answers triggering a flag for concern to be communicated to nurse case managers for follow-up. The goal of this program was early identification of post-discharge complications and timely interventions to avoid emergency department visits and hospitalizations. This program was discontinued due to barriers described as “the manual work required to support the program and notify nurses of patient issues.” In 2009, these same case managers were given access to a new tool, the IVR protocol based “Geisinger Monitoring Program” (GMP), described as “a telemonitoring support system designed for post-discharge patients.” Researchers remark that GMP offered case managers a “scalable solution for automated tracking of patient compliance to the program.”

***Intervention Details***

- “Enrollment in GMP was managed by a case manager who made an initial outbound call to each potentially eligible patient within 24 to 48 hours of a hospital discharge.”
  - Eligibility assessment was inclusive. Case manager called (1) All discharged patients receiving primary care from a medical home clinic site, regardless of diagnosis, and (2) All discharged patients with diagnoses of heart failure, pneumonia, and/or chronic obstructive pulmonary disease.
  - Patients were enrolled if they were “clinically appropriate for the IVR program based primarily on case complexity, including a predictive modeling risk score and general readmission risk.”
    - “The predictive modeling score, similar to the Hierarchical Condition Category risk score but calculated using externally developed software, factored in demographics, clinical condition, pharmaceutical use, and service location to calculate a risk level on a scale of 1 to 5 for each patient. A specific risk score was not required for GMP enrollment, as patients with lower risk scores could still be at risk for readmission and be appropriately managed with telemonitoring.”
  - Patient were excluded from GMP is they had hearing impairment, cognitive impairment, or were unable to receive planned phone calls.
- Once enrolled in GMP, patients received an IVR call once per week for 30 days for a total of 4 IVR calls. Patients, even if they were high risk, were discharged from the telemonitoring program after the fourth week.
  - “Case managers could make additional calls or arrange to see the patient in the clinic, depending on need.”
  - “To support compliance or participation in the program, the IVR system was programmed to automatically retry calls at set intervals if there was no response to the scheduled call.”
- The IVR calls used “branching logic” which tailored questions asked to the patient based on his/her current and previous responses.
- The IVR templates were designed to alert the case manager via the electronic health record system—**in real time**—of any areas that need further follow-up based on the patient’s responses.
  - “Case management follow-up in response to an alert consisted of a review of the questions that prompted the alert, contact, and coordination with the primary care providers as indicated for follow-up appointments, and contact with the patient for medication management, care plan changes or reinforcement as necessary.”
  - “A daily report for review by Case Managers profiled patients who did not answer the IVR call.”
- The IVR program was not intended to replace traditional contact. GMP provided an additional service to extend the reach of the case manager to more patients in the post discharge period.
- The separate IVR telemonitoring program for health failure included a weight monitoring component.
  - “Patients with admissions for heart failure were managed through a separate telemonitoring program that included a weight monitoring component”

***Intervention Outcomes and Impacts***

- “Pre-post parallel quasi-experimental design” was used to evaluate effects on 30-day readmission rates; “A total of 3295 subjects (875 GMP, 2420 controls) were used in the final analysis”.
  - “To adjust for potential selection bias by disease severity, [the researchers] calculated a propensity score for GMP enrollment”
  - “The GMP group had a slightly lower mean risk score and lower prevalence of diabetes and hypertension, but a higher overall rate of readmissions in the baseline period than the control group. After adjusting for propensity score, the differences between cohorts in all of these variables were reduced to acceptable levels (P > 0.05, or standardized difference < 0.10).”
  - Of the 875 patients in the GMP cohort, only 34 (4%) failed to participate in the program for all 4 weeks. Four (0.5%) dropped out voluntarily; the others were disenrolled for other reasons.
- In all, “absent a change in the case management model, patients who were discharged from the hospital were approximately 44% less likely to have a 30-day readmission if they were case managed and participated in the GMP telemonitoring program than if they were case managed only. The robustness of these results was tested by applying a series of regression models using different propensity scoring methods and using different approaches to handling dropouts, none of which substantially changed the findings.”
- More details on effectiveness outcomes include:
  - “Patients in the GMP and control groups did not have significantly different readmission rates in 2007 (16.1% vs. 18.9%, P = 0.43) or 2008 (20.5% vs. 22.9%, P = 0.38), but they did differ significantly in 2009 (15.7 vs. 20%, P < 0.0001).” Ultimately the total difference in readmission rates for the study period was found to be significant, (16.5 vs 20.5%, P < 0.0001).
    - Researchers comment that this “substantial incremental benefit of the GMP program” is an important contribution for consideration by other researcher and health systems.
  - “Within the GMP cohort, admissions during GMP enrollment had a much lower readmission rate than admissions before or after GMP enrollment (10.1 vs. 27.1% and 18.8%, respectively, P < 0.0001).”
  - Within-patient effect of GMP enrollment on the probability of a hospital admission being followed by a readmission with 30 days showed a significant “44% lower likelihood of readmissions for GMP patients than would be expected without the intervention.”
  - “For the intent-to-treat analysis (i.e., treating post-dropout admissions as if the patient were still enrolled in GMP), the estimates of GMP effect were slightly smaller (35% to 40% reductions in readmissions). When those post-dropout admissions were censored, the estimates of GMP effect were slightly higher (50% to 56% reduction in readmissions).”
  - The comparison of results from 3 sensitivity analyses indicated that GMP did not “permanently maintain the same level of reduced risk after leaving the program”
- “In contrast to the earlier manual program that required clerical staff to spend approximately 30 minutes to fully complete a call, the IVR call took approximately 2 to 3 minutes to complete.”

***Intervention Facilitators***

- A previous study looking at a similar telemonitoring program showed large drop-out rates which concerned the GMP researchers. The dropout rate seen for GMP compared to the previous “may suggest that there is an optimal balance among frequency of contact to evaluate patient status, patient burden, and patient engagement.”; Further the researchers “believe that our automated GMP program effectively addressed issues of participation and compliance, a potentially important difference that may have reduced the risk of readmission.”
- Researchers comment that a limitation of the study is that “The IVR technology was added on top of a very robust case management program that had already demonstrated impact on readmissions”, implying that this pre-existing robust case management program may be helping to facilitate the success of the telemonitoring program.
- Researchers comment that “The fact that our study environment was already very accustomed to implementing novel case manager-based programs like GMP may have also contributed to the program’s success and could therefore limit generalizability to other settings with less experience. Implementation experience, however, suggests that the key components of the program could be implemented outside of an integrated delivery model.”
- Researchers comment that “the role of the highly experienced case manager in selecting appropriate patients for the IVR intervention could be an important confounding factor”, implying that the high experience level of these case managers may be helping to facilitate the success of the telemonitoring program.
- Researchers comment that a “a defined staff dedicated to the safe and effective transition of their discharged patient population” is a requirement for implementation of this program; continuing “At the core, a robust program of clinical staff dedicated to the follow-up of patients transitioning to the next site of care and the ability to intervene efficiently on identified patient issues in a timely manner is needed”
- More generally, the automated nature of the IVR protocol inherent to the telemonitoring program was key to the major goal of the intervention to expand the case managers capacity, or to reach and monitor more people after discharge. This automation was not present in the manual program designed to have similar impacts on readmissions, which researchers’ comment was a historical failure for Geisinger. Without the automated aspect provided through the IVR protocol the manual labor required to do this kind of work would have been a barrier.
  - The automated alert system and automatically generated noncompliance review via EHR in coordination with the IVR data further alleviated manpower barriers that may have arisen in connection with an advanced post-discharge program of this scale.

**Intervention 3. Intensive Primary Care with Complex Care Management Model for Adolescents and Young Adults****^25^**

***Intervention Background***

Critical care gaps for youth with specialty care needs are often experienced at the transition between pediatric and adult care. This is because typical adult primary care settings often lack the required resources to support care for complex patients. Adolescents and young adults with special care and health needs (AYASCHN) face risk of worsening health outcomes when transitioning to adult care and may lead to costly care down the line. Some gaps include fragmented, uncoordinated care and planning, crisis-driven management with tendency for overmedicalization, inadequate support for family members responsible for youth, poor communication and lack of education for receiving providers. A complex care model for AYASCHN was developed and implemented for Geisinger to address these gaps and was considered to be an augmentation of the PCMH model. This interventional model came to be called the “Comprehensive Care Clinic” (CCC), described as “intensive primary care.” The CCC’s general goals include (1) enhanced ability to identify high risk patients, (2) comprehensive health assessments with problem solving to reduce need for expensive services and improve care, (3) working closely with the youth’s support system, and (4) responding to changes in condition with efficiency (reducing unnecessary services). The CCC was implemented via The Geisinger Health Plan, a full-service regional plan in its third year of operation at the time of the study. GHP is Medicaid managed plan and those exposed to the intervention were Medicaid recipients, generally representative of the AYASCHN population. This intervention was assessed in terms of impact on cost and care utilization. The study supporting this assessment of the intervention was “conducted as a part of Geisinger Health System’s quality improvement initiative in partnership with GHP for case management and analytic support.”

***Intervention Details***

- A dedicated multidisciplinary care team, including an internal medicine/pediatrics physician, an advanced practitioner, a registered nurse case manager, and a pharmacist, develops care plans to coordinate interactions among the team members as well as with the patient and family caregiver and other health care providers located outside the clinic.
- The care plan is embedded within each patient’s electronic health record and maintained by the team members with input from the patient, family, and all medical care providers.
- To identify the AYASCHN patient population eligible for care in CCC, the following criteria:
  - Reside within Geisinger’s service area to ensure reasonable access and be older than the age of 15; AND of the following: typically use 20 or more medications; rely heavily on specialty care (3+ specialties, 3+ times in the past 2 years; 2+ genetic medicine visits); be technology-dependent (e.g., ventilator, gastronomy tube, wheelchair bound); have certain diagnoses (e.g., cystic fibrosis, spina bifida, cerebral palsy, congenital heart defect, transplant, chromosomal or congenital abnormalities); have a history of frequent emergency department (ED) visits and inpatient admissions; be referred to complex care management via their specialists, primary care physicians, or self-identified by the patient or caregiver.
  - All referrals are reviewed by CCC staff to ensure appropriateness of the referral.
- Multipart comprehensive assessment strategy includes:
  - Extended primary care visit with physician (around 60 minutes) every 3 to 6 months.
  - “Medication reconciliation is performed by the team pharmacist as well as the nurse case manager to identify possible adverse effects, medication interactions, required laboratory and other screening and monitoring tests, and to assess the effectiveness of the current medication regimen.”
  - Comprehensive case management is provided by a nurse case manager who is embedded in the internal medicine clinic and sees the patient and family caregiver together with the physician to help develop the care plan.
- Training and education of the patient and family caregivers are provided, focusing on self-management skills to enable them to recognize signs and symptoms of acute exacerbations indicative of a worsening condition and to follow instructions on how to respond.
- Integrated social services within the CCC team. Substantial time dedicated outside of each visit to coordinate, plan, follow, and document all relevant patient care and social services.
- Same-day access to CCC for emergent care needs is available.

***Intervention Impacts and Outcomes***

- “A review of the list of patients enrolled in CCC as of April of 2015 yielded 83 unique patients who also were members of the GHP Medicaid plan.”
  - “The most common primary diagnoses among these patients were autism spectrum disorder and spina bifida, and 38.6% of them were technology-dependent.”
  - “CCC enrollment (represented by 658 member-month observations) had coincided with member-month observations that were characterized by patients who were older, more likely to be enrolled in GHP’s case management program, and had higher risk scores relative to the member-months when the patients had not yet enrolled in CCC (represented by 767 member-month observations). Consequently, CCC enrollment appears to be associated with higher cost and care utilization, although the differences are not statistically significant.”
- “CCC enrollment was associated with approximately a 78% reduction in acute hospital admissions (P = 0.053) and approximately 60.3% in ED visits (P = 0.017) per member per month.”
- “CCC enrollment was associated with an approximately 28% reduction in PMPM total cost of care ($3931 observed vs. $5451 expected; P = 0.028). The most significant source of the total cost reduction appears to be inpatient cost (P = 0.028).”
- Covariate regression models were used to strengthen the validity of the data for the following covariates: seasonality, enrollment in other care management programs, location of PCP, and length of time enrolled in CCC during the study period. Confounding factors, including selection bias for those with worse health in the CCC group and dual eligibility for Pennsylvania State’s waiver program which help to alleviate costs to GHP, both would work to lessen significance and were also alleviated through use of covariate regression models.
  - “Many of the eligible AYASCHN patients are transitioned to CCC at end-stage disease […] requiring prolonged hospitalizations and change in therapies […] Therefore, it is expected that a simple comparison over time between the periods before and after CCC enrollment would show a pattern suggesting that CCC enrollment seems to be associated with higher cost and utilization[ …] Yet, the reported results obtained after controlling for the confounders [imply] that CCC is likely to have alleviated the increasing disease burden over time. This also implies that the reported results may be subject to a downward bias that underestimated the true CCC impact.”
- “Although the magnitude of the estimated impacts seems large, it may simply reflect the extent of the problems and challenges inherent in caring for this population. At the same time, these results also imply that the significant challenges of managing and coordinating care for a Medicaid AYASCHN population can effectively be translated into patient care improvements and medical care cost reductions that can be realized rather than just hypothesized.”

***Intervention Facilitators***

- Researchers comment that the exact mechanisms supporting the success of the intervention remain unclear “it is not clear from the available data whether the observed reductions in cost and care utilization under CCC were attributable to enhanced caregiver engagement, improved care coordination across providers, or better medication management via pharmacist services. Further studies are necessary to provide more insights on this issue” and generally do explicitly comment on facilitators.
- Dually trained internal medicine-pediatrics physician may have helped to alleviate common gap in lack of education on pediatric issues AYASCHN face when transitioning into regular adult primary care.
- Protected provider-patient time through regular, extended visits paired with focus on patient/family self-management education may have helped to facilitate shared understanding of patient needs and goals and how to achieve them.
- Availability of same day services may have helped alleviate seeking emergency care unnecessarily.
- Care plan in EHR that specify areas of responsibility for care team – dual presence of case manager and physician during appointment time may have helped to contribute to the success of these care plans.

Case Study 2. Kaiser Permanante

**Institutional Profile**

***Institutional Mission*^26^** *–* “Kaiser Permanente exists to provide high-quality, affordable health services and to improve the health of our members and the community we serve”

***Institutional Vision*^26^** *–* “We are trusted partners in total health, collaborating with people to help them thrive and creating communities that are among the healthiest in the nation.”

***Key Facts*^26,27^** *–* Kaiser Permanente is an integrated, multi-state, closed health system and nonprofit health plan that serves 12.2 million plan members. Kaiser is headquartered in Oakland, California and operates 39 hospitals and 706 medical offices spread primarily throughout the Western United States with additional service areas of Hawaii, Georgia and the greater Washington, D.C. and Baltimore area. In 2018, they reported an operating revenue of $79.7 billion and 218,297 employees including approximately 23,000 physicians and 60,000 nurses.

***Characteristics*** *-* Kaiser Permanente provides care through three entities: Kaiser Foundation Health Plan, Inc., the Kaiser Foundation Hospitals and the Permanente Medical Groups. Kaiser’s care delivery is defined by a capitated payment structure, opposed to fee-for-service, that holds Kaiser fiscally and clinically responsible the health status and outcomes of the populations they serve.^28^ The health plan and medical groups are aligned to a global budget through a risk sharing arrangement which motivates the health management teams to optimize utilization.^29^ As part of this integrated delivery model, Kaiser focuses on preventative and chronic disease care, consolidates as many services as possible in one location,^30^ provides comprehensive medical record access for every Kaiser provider, facilitates clearly defined care pathways with role definition and clinical decision support, offers financial incentives to high performing teams, and carefully tracks a variety of metrics to develop human capital, budgets, and algorithms.^29^ As early as 2007 Kaiser began to model itself as a “learning health system” which capitalizes on information technology to drive improvements in the quality of care.^31^

***Trends in Patient-Centered Care and Care Coordination*** *-* In our review of Kaiser’s peer-reviewed patient-centered care and care coordination interventions, we found the majority of their work has centered on streamlining care processes, primarily in peri-surgical and hospital care.^32-6^ These care process interventions are conceptualized of as “bundles” and involve a set of care elements being applied to each patient relevant to the intervention usually with risk stratification to tailor the elements by patient need. There were a few other recurring intervention types: provider professional development through coaching and communication training,^37-40^ improved screening processes,^41-2^ optimizing EHR use and EHR-integrated services^43-9^ like clinical decision support and automatic patient-facing reminders or calls using speech recognition, mindfulness-based stress reduction services,^50^ and teleservices including telemonitoring^51-2^ and telestroke care.^53-4^ Additionally, an investigation of Kaiser’s websites revealed programs relevant to patient-centered care and care coordination in accordance with the peer-review findings. These include support groups, shared medical appointments (SMAs) and coaching programs available in person, via phone, or virtually for a large variety of issues like smoking cessation, gun violence, diabetes, pregnancy, and general wellness, and more. Notably, in 2019 they opened a new telepsychiatry program and an 18-bed medical-psychiatric unit as part of a 7-year, therapists’ union-led effort to address the mental health crisis in Oakland’s Alameda and surrounding counties; this expansion of mental health services is integrated with the local health departments and represents a $700 million investment.^55^

***Comment on Kaiser Interventions Selected*** *-* Interventions selected include (1) a post-discharge protocol, (2) a nurse hand off protocol, and (3) a telepharmacy service line. These interventions were all implemented in different regions of the Kaiser health system. These interventions were selected after review of 48 peer-review articles featuring studies taking place at Kaiser facilities that were published by or affiliated with Kaiser Permanente personnel.

**Intervention 1. Kaiser Northwest’s Five-element Hospital-to-Home Transitional Bundle^56^**

***Intervention Background***

Readmissions are used a quality marker to access health system performance. The Centers for Medicare and Medicaid Services now require public reporting on this metric with financial penalties in place for excessive readmissions. A 2012 meta-analysis estimated an average of 23.1% of readmissions were avoidable. In 2009 Kaiser Permanente Northwest (KPNW) developed a unique “transitional care bundle” that sought to address all-cause readmissions and included patient and caregiver input. KPNW serves 500,000 members via 900 physicians and 9,000 staff at 27 outpatient clinics, 2 medical centers with contracts with several community hospitals. During the first phase of the project a variety of methods were used to understand KPNW’s own challenges with readmissions. These methods include patient and provider interviews; video ethnology; chart review with follow up interviews with patients, caregivers and the care teams involved; patient satisfaction scores; and a review of administrative data. This phase of the project identified 5 tangible patient needs which are described using patient’s words. Next Kaiser Permanente’s Care Management Institute selected KPNW physician and health plan leaders to convene stakeholders and develop potential strategies to address these 5 needs. Over 6 months, over 50 Plan-Do-Study-Act were completed and their results reviewed in weekly meetings with stakeholders to produce 5 “transitional bundle” elements that directly correlate with each of the 5 patient needs, detailed the chart below.

| ***Patient Need*** | ***Transitional Bundle Element*** |
| --- | --- |
| “I will have what I need when I return home” | Risk stratification into high, medium or low risk for readmission. |
| “I know when I should call and what number to use” | Specialized “post-hospital hotline” for patients |
| “My regular doctor will know what happened to me in the hospital” | Standardized same-day discharge summaries and instructions |
| “I will see my doctor soon after hospitalization, and I know someone will check on me when I am home” | Timely post-hospital follow-up |
| “I understand my medications, how to take them, and why I need them” | Medication reconciliation |

All patients admitted and discharged from the participating KPNW 269-bed hospital received the same “bundle” of interventions which was delivered by nurses, pharmacists, and hospitalists. The intervention was introduced in a staggered way to the internal medicine service first and then to specialty services in the following year, with the research team tracking outcomes on a rolling basis as implemented.

***Intervention Details***

- For patients in high or medium risk groups, additional follow up and medication reconciliation was provided.
- Criteria for high risk stratification was flexible – any nurse, pharmacist or physician could identify a high risk patient.
- Any patient who met specific criteria was automatically classified as high risk. These criteria were 1. Any diagnosis of heart failure, whether or not it was related to the admission, OR 2. An admission within the previous 30 days
- Specialized “post-hospital hotline” provided a single point of contact between discharge and the first follow up appointment
- Calls could be transferred to priority nurse advice line and triaged 24/7.
- Advice nurses handled the calls they could and paged an on-call hospitalist or specialist for those they could not. This was facilitated through a special agreement brokered by the hospitalists and PCPs leaders that extended hospitalist oversight and responsibility for 48 hours after discharge before transferring to primary care.
- Standardized patient discharge instructions included discharge diagnosis (in layman’s terms), diet, activity, ‘‘call if/return if’’ instructions specifying signs and symptoms warranting a phone call to a physician or nurse, the hotline phone number, follow up tests and appointments, and any additional instructions.
- All ambulatory care appointments were made before discharge. Follow up appointments occurred within 5 days for high risk patients and within 10 days for all others.
- Follow up appointments were listed on the patient’s discharge summary.
- Transition registered nurses (RNs) followed up with patients by phone within 48 hours and provided case management services to high risk patients for 30 days.
- All patients received medication review and reconciliation at admission and an accurate medication list with instructions in lay language on discharge.
- Transition care pharmacists prioritized high risk patients for medication reconciliation at discharge, based on age, medications, and chronic conditions. 2 FTE transition pharmacists were hired to implement this intervention.
- After discharge, transition RNs reviewed medications during 1 or more follow up phone calls. In some cases, determined in partnership with a transition RN, pharmacists provided outreach calls.

***Intervention Outcomes and Impacts***

- An average of approximately 18,500 discharged were included annually, with the mean age being 54.9. In April 2014, at the end of the data period presented, 50% of all patients discharged were at high risk for readmission – 68% of these patients had an appointment within 5 days and 56% had pharmacist medication reconciliation.
- Kaiser’s measure of readmission rates using the number of index discharges with readmission within 30 days overall index discharges decreased significantly from ~12% in 2008 to ~10% in 2014 (*P*<0.0001)
- Combined risk-adjusted ratios of observed to expected 30-day readmissions for Medicare and commercial plan holders decreased significantly from 0.71 to 0.66 (*P*<0.0001) which reflected a shift from the 75^th^ to the 90^th^ percentile nationally
- HCAHPS discharge instruction composite scores increased significantly from 80% to 90% (P<0.0001) which reflected a shift from below the 50^th^ percentile to the 90^th^ percentile nationally.
- In the first year the HCAHPS care transition measurement was used, 2013, the KPNW hospital earned a rating of 53%, just under the 90^th^ percentile nationally.
- Between 2008 and 2014 average time between discharge and first follow up appointment for high risk patients on the internal medicine service decreased from 9.9 to 5.3 days (*P*<0.0001)
- Errors on the discharge medication list decreased from a baseline of 57% to 21% in April 2014 (P < .0001). Most errors are detected and corrected before discharge by the pharmacist performing medication reconciliation.
- Similar results were found at other Kaiser Permanente Regions that adopted the bundle.

***Intervention Facilitators***

- Bundle was developed with input and oversight from patients and caregivers, including a member of the health plan present for all stakeholder meetings.
- A comprehensive electronic health record “KP Connect” is integrated across all KPNW settings and all providers have access to the same records.
- KPNW identified care transitions as a high priority quality improvement opportunity.
- Investment of resources into data collection towards intervention design and continuous improvement.
  - Qualitative and quantitative methods were used in the design stage to identify issues in transitional care in terms of patient need and health system gaps as revealed in the EHR and administrative data.
  - Throughout project’s implementation there was continuous tracking of outcome and quality measures. Department-level readmission data helped to garner department chiefs’ ongoing attention and support.
- Pre-existence of the Kaiser Permanente Care Management Institute (CMI), which supported the engagement of health system stakeholders, the “breaking down” of “silos between settings and stakeholder groups,” and oversaw the pilot design and implementation.
- Incremental implementation, Plan-Do-Study-Act cycles, and weekly meetings with stakeholder groups facilitated meaningful, ongoing development of the intervention.
- Pre-existence of a priority nurse advice line to re-direct callers from “post-hospital hotline” as needed.
- Institutional flexibility and willingness to work cross departmentally to achieve goals.
- Sustainability mechanisms in place in the post-implementation phase: transition care leaders continued to report data on transitions care outcomes to hospital leaders on an ongoing basis; recognition of the need to include the transitions care bundle in orientation to mitigate sustainability threats from staff turnover.

**Intervention 2. Kaiser Southern California’s Nurse Knowledge Exchange Plus (NKE*Plus*) Handoff Tool^57^**

***Intervention Background***

Recognizing the Joint Commission National Patient Safety Goal related to standardizing handoffs to reduce medical error, effective from 2006 until 2009, as well as the movement to bring handoff communication to the bedside, Kaiser designed and implemented an intervention called Nurse Knowledge Exchange*Plus* (NKE*Plus*)*.* This intervention was originally designed and implemented between 2004 and 2006. In 2009 Kaiser’s own Innovation Consultancy collaborated with frontline staff to address inconsistencies and variations in the handoff procedure. By spending more than 200 hours observing, shadowing and interviewing frontline staff and 2,000 hours of piloting among 150 participants across disciplines, the Consultancy made improvements to the handoff protocol. Because Kaiser finds it difficult to spread evidence-based best practices beyond pilot sites, they used a human-centered implementation (HCI) approach which allows each unit to customize the minimum specifications of the intervention and have ownership over the resultant practices. This strategy was used to implement NKE*Plus* in 125 nursing units at 14 Kaiser Permanente Southern California medical centers.

***Intervention Details***

Element 1: NKE*Plus* minimum specifications, unique adoption and additions per unit

- Team rounding in the last hour before shift changes to address patient needs, minimizing potential for interruptions to handoffs.
- While nurses departing prepared for handoff, charge nurse made pre-shift patient assignments intended to limit the number of departing nurses each oncoming nurse would have to hear from. Eliminates inefficient assignment “brokering” by oncoming staff.
- Unit support for uninterrupted bedside reporting:
  - Charge nurses, unit secretaries, and nursing assistances expected to minimize disruptions during hand-off time.
  - Unit secretaries answer telephones and call lights, with responses delegated to NAs or charge nurses as needed, and having departing charge nurses handle any unanticipated patient needs or admissions that overlap with shift change.
- At bedside: standardized report and safety check for each hand off.
  - Standardized reporting format (using acronym “KP SMILE”: Know the patient, Professional exchange, Snapshot of symptoms, Medication, Intake and output, Labs, Education for patient)
  - Standardized safety check format (using acronym “HEAL/S”: High alert meds, Equipment, Alarms, Lines, Skin or Sensitive issues)
- At bedside: nurses conducting hand off collaborate with patient to update in-room care boards and patient goals.

Element 2: Human-centered Implementation (HCI)

- Pilot units selected had strong leadership, high level of variation in reporting or high number errors arising from low patient and family engagement, readiness for change assessed through number of requests for support and absence of competing priorities, and available staff members who would serve as champions to model improvement and assist with rapid cycle improvement.
- Pre-meeting with unit leadership to discuss roles and responsibilities for the unit regarding pilot actors and recommend champions.
- Two-day champion orientation. Prior to orientation champions asked to conduct informal conversations with coworker to access readiness for change and current challenges related to shift change.
  - Essential aspects of the orientation were to provide information about the intervention, to develop leadership skills and approaches to potential resistance, and to engender commitment to improvement challenges the unit faced through sharing personal experiences.
- Champions per unit designed NKE*Plus* for their unit and tested over a variable number of weeks, gathering feedback through formalized and informal methods. One method was having a storyboard depicting the intervention with room to comments written on the board.
- Regional quality staff supported implementation efforts via
  - Monitoring, ongoing coaching, and monthly web and tele- conferences to share effective strategies.
  - Annual site visits with Chief Nurse Executives that included assessments and action plan development related to strengths, weaknesses, opportunities, and threats to the sustainability of the pilot.
  - Ongoing summits with regional leaders and NKE*Plus* representatives - unit leaders and champions -from each medical center to share successful practices
  - Encouraging each unit to make video recordings of their NKE*Plus* practices and produce educational videos about NKE*Plus*. Regional quality staff offered editing services for these videos.
  - Each medical center had quarterly meetings that included reports on NKE*Plus*-specific measures.
- Metrics using HCAHPS elements were developed to assess progress and consistency of intervention. This was called “NKE nursing behavior bundle” based on the following four HCAHPS elements: (1) the departing RN introduced the patient to the oncoming nurse; (2) the care board was updated; (3) the RN reviewed the patient’s care in a way that he or she could understand; (4) the RN asked the patient for input regarding his or her care.
- For particularly resistant departments, special attention and resources were invested to garner buy in. A special web conference with a parallel unit within a high reputable hospital was organized for best practice sharing – all KP leads for NKE*Plus* participated in the 90-minute event. Additionally, specially solicited patient and caretaker feedback and specially arranged role-play activities with patients and caretakers were organized with a pre-existing patient/caretaker Advisory Council. Input and video recording of these role play activities and patient/caretaker perspectives were incorporated into future orientation materials for units of this kind.

***Intervention Outcomes and Impacts***

- By the end of 2012, 100% of the 64 medical/surgical units and 47 (77.0%) of the 61 specialty units in Kaiser Permanente Southern California medical centers had implemented NKE*Plus*. By May 2013, all specialty units but one had implemented it.
- The mean regional HCAHPS scores for nurse communication improved from 73.8% in 2010 to 77.4% in the first quarter of 2014.
- The mean HCAHPS score for 82 nursing units across medical centers with comparable data improved from 73.1% (standard deviation [SD], 3.5) in 2010 to 76.4% (SD, 4.9) in the first quarter of 2014 (p < .001). Aggregated scores for nursing units within medical centers improved by 0.2 to 5.9 percentage points, and HCAHPS scores for nursing units at six medical centers improved by one percentile rank.
- The mean score for the region on the NKE nursing behavior bundle improved from 65.9% in 2010 to 71.3% in the first quarter of 2014. The mean score for 60 nursing units across medical centers improved from 65.7% (SD, 4.6) in 2010 to 70.4% (SD, 5.0) in the first quarter of 2014 (p < .001). Aggregated scores for nursing units within medical centers improved by 0.1 to 7.8 percentage points.

***Intervention Facilitators***

- Pre-existence of Kaiser Permanente’s Innovation Consultancy which since 2003 has used human centered design methods and principles to implement new ways to improve the care experiences of patients and the work experience of care providers.
  - The Consultancy team was significantly involved in the design and implementation and partnered with regional departments including quality and safety, nursing, and care services leads.
- The Consultancy and regional partners worked together in the implementation process to ensure collaborative, meaningful, and “customized” adoption of the intervention in autonomous nursing units.
  - Some medical centers or unit leaders felt pressure to cut short the customization phase and authors report that the length of time that medical centers and nursing units allowed for staff-led design was directly related to the robustness of implementation.
- The pre-existence of a comprehensive EHR integrated across all care setting with all-provider access allowed for a key component of the intervention, an acronym to guide hand offs, to be based on a shared, familiar tool already in use.
- Flexibility built into the intervention design through use of “minimum specifications” that were built upon for unit-specific challenges through use of rapid improvement cycles and conveniently placed feedback solicitation materials.
- Incremental implementation, initial pilot units selected based on potential for success.
- Frontline staff “NKE*Plus* champions” and patient and caretaker advisory councils helped garner staff buy-in.
- Extensive support to frontline and regional staff through professional development to help them lead implementation and sustainability practices, and through channels to help them share skepticisms, successes, strategies, and challenges.
- Patient-centered and bottom-up produced NKE*Plus* education materials were used to motivate support for the intervention and incorporated into orientation for new employees, new nursing graduates and traveler nurses.

**Intervention 3. Kaiser Colorado’s telepharmacy service to reduce patient burden and improve outcomes around transitions-of-care^58,59^**

***Intervention Background***

Kaiser Permanente Colorado Region (KPCR) created a clinical pharmacy call center (CPCC) in 1996 after an audit showed that the third most common call to the regional call center were calls about drug therapies. The regional call center is the central contact for KPCR members with questions related to their health care needs. KPCR employs 4,000 staff members through the Kaiser Foundation Health Plan and contracts with 800 physicians through the Colorado Permanente Medical group. Six hundred of these staff work for the pharmacy department which offers clinical pharmacy services to all of KPCR through primary care, specialty care, drug information, home IV pharmacy and oncology pharmacy services. Additionally, the pharmacy department runs centralized services including an anticoagulation service, cardiac risk service, clinical pharmacy international travel clinic and the CPCC. The stated goal of the CPCC is to assist the medical group in providing pharmaceutical care to the approximately 400,000 members KPCR serves. Prior to the development of the telepharmacy service, the regional call center directed drug therapy related questions to their nurses, who then needed to request help causing long response times. As part of the initial telepharmacy program, a full-time clinical pharmacist replaced a full-time nurse to assist the nurses at the regional call center. This program has now grown to 24 FTE personnel who take calls redirected from the nurses by work closely with the clinical pharmacy service lines and 18 KPCR medical offices to execute pharmaceutical care. This telepharmacy service was developed through collaboration by the pharmacy department, the medical group, and the Kaiser Foundation Health Plan. The CPCC works with KPCR’s Continuing Care Department and Chronic Care Coordination Department to improve transitions from SNF to home and hospital to home.

***Intervention Details***

- 24 FTE personnel including a clinical pharmacy chief, a clinical pharmacy supervisor, 17 clinical pharmacists, and 7 pharmacy technicians
  - Technicians support the pharmacists by answering calls from members with nonclinical questions.
- The CPCC staff is available for calls 7:30am to 6pm M-F and Saturdays 8am to 12pm.
- Staff is housed in the regional call center and works closely with the regional call center physicians, nurses and service associates. The CPCC pharmacists collaborate with the regional call center physicians about medication changes and are able to seek additional medical advice as needed.
  - When a KPCR member calls the regional call center, they can expect a service associate to answer the phone, help them book appointment, and send messages to medical office. Additionally, they may be redirected to a nurse or physician to triage acute illnesses, or to the CPCC for questions related to drug therapies.
  - In 2000, the CPCC collaborated with the physicians at the call center to develop evidence-based guidelines for seasonal allergic rhinitis that included tele-triage, patient education and treatment protocols.
    - Any member calling the regional call center to schedule an appointment for allergic rhinitis were redirected to the CPCC for evaluation in accordance with these guidelines.
- Call types include:
  - New member screening and medication reconciliation for those transitioning into KPCR with history of long-term drug therapy.
    - These calls are typically 20 minutes long and involved a detailed patient medical history and social risk interview.
    - After this call the CPCC staff reaches out to former pharmacies for medication reconciliation.
    - CPCC may order laboratory testing prior to the new member’s first primary care appointment.
    - CPCC may issue one-time prescriptions with authorization from call center physicians prior to the new member’s first primary care appointment.
  - Members recently discharged from non-KPCR hospitals
  - Notifying members about changes in Medicare benefits
  - General questions about new drugs or drug-related news releases, with call center staff having ability to investigate medical records to see if the news is relevant to the member.
  - Members and medical personnel with medication questions
  - Member prescription refill requests
- CPCC staff has access to the entire medical record and may send progress notes to primary care providers, or other pharmacy services like the anticoagulation service, to alert them of recommendations.
- CPCC staff is able to send their prescriptions directly to the Kaiser pharmacies to assist members in obtaining their medications in a timely manner.

***Intervention Outcomes and Impact***

- The CPCC answers approximately 1,200 telephone calls per day.
- As of 2005, through 9 years of operation, the CPCC helped transition 50,000 new members.
  - Approximately 30% of these calls are Medicare beneficiaries who are prime benefactors of the screening and medication reconciliation services.
- Random chart review of these new member screenings showed a cost avoidance of $324 per member per year thanks to medication reconciliation, representing a cost avoidance benefit to KPCR members of $16.2 million dollars over the period of 2000-2005.
- Savings to KPCR amounted to an estimated $4.3 million in avoided clinic costs.
- In a case–control study, a subgroup of 115 patients triaged and treated for allergic rhinitis by CPCC pharmacists between March 1, 2000, and September 30, 2001, was compared with a group of patients who received standard care during a clinic visit by a physician. Patients triaged by CPCC pharmacists were more likely to receive a nasal corticosteroid prescription than control patients (90.7% versus 77.8%, respectively) (p = 0.009).
- In a quasi-experimental study of patients with discharge summaries received by CPCC within 48 hours of SNF discharge versus those whose discharges were not received and directed to primary care for first follow up (Usual Care Group):
  - Review of medication-reconciliation records for intervention patients indicated that >90% of all discharge summaries contained at least one potential drug-related problem including duplicative drugs, omitted therapy, and medication contraindications.
  - After SNF discharge, patients who were transitioned by CPCC clinical pharmacists were: 1) 78% less likely to die; 2) 29% less likely to need an ED visit: and 3) 17% more likely to follow-up with primary care clinicians than were patients in the Usual Care group

***Intervention Facilitators***

- Pre-existence of a regional call center to house, support and triage telepharmacy services.
- Pre-existence of a comprehensive electronic medical record that pharmacists and all providers have full access to.
- Pre-existence of Kaiser’s vertical integration allows the CPCC to interact with Kaiser owned pharmacies.
- Pre-existence of a Continuing Care Department that produces a discharge summary for members discharged from SNF and LTC facilities and forwards it to CPCC on the same day of discharge. Components of the summary had input from CPCC.
- Pre-existence of a Chronic Care Coordination Department with at least one staff member per outpatient clinic to receive CPCC chart notes after transitions of care related medication reconciliation.
- Bidirectional information exchange between medical offices, pharmacy services and CPCC is encouraged through EHR in-basket communication addressed to the clinics, coordination teams, and CPCC as a whole rather than individuals.
- Members of the CPCC team have substantial training in polypharmacy detection and management
- The CPCC and the regional call center associates, nurses and physicians work in close, daily proximity to each other with clear responsibilities identified for the key call center roles.
- The CPCC has established protocols for communication, documentation and interactions with patients and caregivers.
- “After hours” and “weekend hours” built into the program’s operation.
- An audit identified a problem in the call center leading to the development of what became a robust program. Intervention to address the problem started small and grew from there.
- KPCR pharmacy department values high-quality and cost-effective pharmaceutical care, therefore regional drug therapies initiatives are common and the CPCC is encouraged to be involved in special projects related to drug therapy and transitions of care improvement.

Case Study 3. Cleveland Clinic

**Institutional Profile**

***Institutional Mission^60^*** *–* “To provide better care of the sick, investigation in their problems and further education of those who serve.”

***Institutional Vision*^60^** *–* “Our vision for Cleveland Clinic is to be the best place for care anywhere and the best place to work in healthcare.”

***Institutional Values^60^*** *–* Quality & Safety, Empathy, Inclusion, Integrity, Teamwork, and Innovation.

***Key Facts*** *–* Cleveland Clinic is a nonprofit, multi-specialty academic medical center and health system that serves 2.4M patients annually.^61^ Headquartered in Cleveland, Ohio, Cleveland Clinic provides care primarily to 21 counties in northeast Ohio covering a regional population of approximately 4.4M.^62^ Cleveland Clinic operates a total of 18 hospitals, including 11 regional hospitals in Northeast Ohio, and 210 outpatient locations. Cleveland Clinic has several smaller service areas outside of Ohio: five hospitals in Southeast Florida, specialized facilities in Las Vegas, Nevada and Toronto, Canada, and a quaternary care hospital in Abu Dhabi. In 2021, Cleveland Clinic plans for a hospital in London. Cleveland Clinic brings in a $10.6B operating revenue and employees 67,554 employees, called “caregivers,” including 4,520 physicians and scientists, and 14,458 nurses. 51,731 of these employees are based in Ohio.^61^ In 2017, Cleveland Clinic Medical Center had 39,126 inpatient discharges from zip codes in the Northeast Ohio region, which was approximately 75% of its total discharge count.^62^ On the whole the Cleveland Clinic organization saw approximately 309,000 admissions in 2018 and 10M outpatient visits.^61^

***Characteristics*** *–* Cleveland Clinic’s operational units all report up through governance structure of three boards: The Board of Directors is the primary governing body, the Board of Trustees act in an advisory role to the Directors, and the Board of Governors, made up of primarily physicians, oversees medical and surgical activities. These Boards govern the executive leadership which operates from the Cleveland Clinic Medical Center and in turn governs leadership at the individual institutes and facilities.^63^ In 2009 Cleveland Clinic redesigned their care delivery model to be centered around patient need by rather than profession-oriented organization.^64^ This was part of a leadership change that came with a new strategy priority of patient-centered care after low patient satisfaction scores were released that put them at the 55^th^ percentile. Another change that was made was referring to all employees including facilities staff as “caretakers” after realizing that patients interacting with the health system spent the least amount of time with physician versus all other employees. Part of the caretaker initiative was an all-employee mandated half day training in which all levels of staff shared experiences of caring for patients and were taught relationship building and customer service skills; over the course of the year it took for everyone to participate, the program cost $11 million. This is part of a parallel, formalized effort to engage employees to problem solve workflows and improve patient care, part of their continuous improvement model.^65-6^ The now-established Department of Continuous Improvement’s stated goal is to build the capacity of all caregivers to improve care processes through training and materials for staff of all levels.^67^ While the impact of these efforts have not been directly quantified, Cleveland Clinic’s patient satisfaction scores overall and in various key domains dramatically improved in the years following.^68^ Cleveland Clinic sees itself as a model for the future of health care, and champions integrated and innovative healthcare delivery systems that have regional location linked through multi-model transport and information technology. Cleveland Clinic’s key learnings of the healthcare system of the future include: (1) efficiency in workflow improve access, (2) accountable care, such as that offered through ACOs, engenders patient-centered care, (3) transparent self-evaluation is essential to quality assurance, and (4) staff structure and policies directly impacts whether patient need is prioritized. Of interest to these latter two points, Cleveland Clinic has all physicians on salary with one-year contracts with renewal subject to performance review,^63^ and is the first major medical center to publish annual outcomes and volume information for its medical specialties.^67^

***Trends in Patient Centered Care and Care Coordination*** *–* In our review of Cleveland Clinic’s peer-reviewed patient-centered care and care coordination peer review articles, we found a range of patient-centered and care coordination intervention types. These include professional development trainings,^69-70^ improving quality of ethics consultations,^71^ shared medical appointments,^72^ shared decision making aids,^73^ multidisciplinary models for chronic or longer-term conditions,^74-6^ post-discharge programs to reduce readmissions,^77^ and machine learning to facilitate efficient and accurate care.^78^ Nearly all of these intervention types were reflected in the programs found through investigating the institution’s websites. These include a variety of shared medical appointments and programs through their functional and integrative medicine departments, and an e-ethics consultation service.^79^ Additionally, Cleveland Clinic has a number of departments intended to directly coordinate transitions of care and enhance the patient-centered experience, including health centers aimed at improving minority health.^80^ Most relevant to care coordination is the Center for Connected Care, which provides hospice, a variety of home care services, and a “connected care” service line for SNF and LTACH which allows patients to take their Cleveland Clinic care with them to local outside facilities. Through this program, Cleveland Clinic providers visit daily to direct care and continue to record treatments and progress in the Cleveland Clinic EHR.^81^ Evidence supporting the Center for Connected Care service line are explored in the selected interventions. In 2017, Cleveland Clinic instituted the Cleveland Clinic Care Community to act a population health unit. This unit operates primarily through approximately 50 embedded primary care nurse coordinators who use algorithms to identify patient risk. This program is reportedly responsible for a 47% decrease in hospital readmission rate.^82^ Additionally, we found that Cleveland Clinic has several departments designated under the special expertise category of “Patient Experience”^83^ all of which support the patient experience; these departments include the Center for Bioethics, Center for Spiritual Care, Cleveland Clinic Experience Partners (a client-based consulting service),^84^ Department of Continuous Improvement, the Office of Patient Experience, and the Quality and Patient Safety Institute. The latter three have in common a focus on the use of data and multidepartment collaboration to enhance patient experience.^85-7^

***Comment on Cleveland Clinic Interventions Selected*** - Interventions selected include (1) a continuing care model for SNF patients, (2) a novel communication and relational skills training for physicians, and (3) a rapid response transporting team for stroke modeled after and partnering with EMS. These interventions were selected after review of 31 peer-review articles featuring studies taking place at Cleveland Clinic facilities that were published by or affiliated with Cleveland Clinic personnel.

**Intervention 1. “Connected Care” Transitions-of-Care Model to Improve 30-Day Readmission Rates from Skilled Nursing Facilities^88^**

***Intervention Background***

Of hospitalized U.S. Medicare beneficiaries, approximately 20% are discharged to skilled nursing facilities (SNFs) to receive post-acute care. Hospital readmittance among these patients is high with 23.5% returning to the hospital within 30 days. Reducing readmissions is important for hospitals because hospitals are penalized for higher than expected readmissions rates under the Hospital Readmissions Reduction Program. At the same, SNFs now receive incentive payment based on their readmissions as part of the Skilled Nursing Facility Value-Based Purchasing program. For these reasons, hospital-SNF partnership models are an attractive target for readmission reduction efforts. In general, patients discharged to SNFs have complex medical conditions, lower functional status, and a higher risk for mortality than patients who are sent home. As of article publication, regulations at the federal level only require patients discharged to skilled nursing facilities be seen within a 30-day period and only once every 30 days for the first 90 days post-initial assessment. To address this issue, the Cleveland Clinic developed a program, “Connected Care SNF,” in which hospital-employed physicians and advanced practice professionals visit patients in select SNFs 4 to 5 times per week to both reduce preventable readmissions and to identify which patients benefit most from the more targeted, patient-centered Connected Care model. This intervention was implemented at a total of 7 SNFs within a 25-mile radius of the Cleveland Clinic main campus hospital.

***Intervention Details***

- The Connected Care program was implemented in 7 of 110 skilled nursing facilities (SNFs) identified within a 25-mile radius of the Cleveland Clinic Hospital System. The remaining 103 SNFs practiced “usual-care” and served as intervention impact controls.
- The Connected Care team consisted of two geriatricians (one—a palliative medicine specialist), one internist, one family physician, and five advanced practice professionals (i.e. nurse practitioners and physician assistants).
- The Connected Care team was available on site during working hours and provided telephone coverage during nights and weekends.
- Essential Connected Care team activities include:
  - Access to hospital electronic medical records (EMRs)and reviewed patient information prior to admission to SNFs.
    - Connected Care “physicians were alerted [of patient’s transitioning to SNF] prior to hospital discharge.”
  - “…an initial comprehensive visit generally on the day of admission to the SNF and always [occurred] within 48 hours.”
  - “Round daily during weekday and within 48 hours visit for weekend admissions.”
  - Discussion of “goals-of-care” with patient upon admission to SNF.
  - Communication with the discharging physician and specialists familiar with patients as needed.
  - Connected Care team reviewed medications and worked closely with SNF staff members to deliver medications.
    - At the SNFs, emphasis was placed on timely medication administration and physician communication.
- Connected care team visited SNF patients 4-5 times per week.
- Connected Care providers were evaluated on “30-day readmission performance rather than productivity.”
- The Connect Care team and SNF staff met monthly with multidisciplinary teams to review 30-day readmission outcomes and other quality measures for continuous quality improvement.
  - These meetings were led by the Connected Care team.
  - “There were monthly meetings at each participating Connected Care SNF. Physicians reviewed monthly 30-day readmissions and performed root-cause analysis. When they discovered challenges to timely medication and treatment delivery during daily rounds, they provided in-services to SNF nurses.”
- “Patients at Connected Care SNFs who had their own physicians, including most long-stay and some short-stay residents, did not receive the Connected Care intervention. They constituted less than 10% of the patients discharged from Cleveland Clinic main campus”

***Intervention Outcomes and Impacts***

- During the intervention phase from 2013 to 2014, adjusted 30-day readmission rates declined at intervention SNFs (28.1% to 21.7%, P <0.001), while there was a slight increase in 30-day readmission rates at control SNFs (27.1% to 28.5%, P < 0.001). This trend may point to an imminent need to reassess both “usual-care” practices at the intersection of high-risk patient discharge to SNFs and the frequency with which these patients interface with clinical staff to review treatment plans.
- Absolute reductions ranged from 4.6% for patients at low risk for readmission to 9.1% for patients at high risk for readmission with medical patients benefiting more than surgical patients. These results highlight the effectiveness of the Connected Care program across HOSPITAL score groups (i.e. low, intermediate, and high-risk patients). With this, it may be possible to reduce hospital readmissions beyond the initial scope of the intervention.

***Intervention Facilitators***

- Connected Care providers facilitated smooth care transitions from the hospital to intervention SNFs via recurrent physician-lead reassessments
  - “Although prior to admission, they were considered hospital level of care and received a physician visit daily, on transfer to the SNF, relatively little medical care is available… The Connected Care program physicians provided a smooth transition of care from hospital to SNF as well as frequent reassessment.”
  - Similarly, researchers comment that Connected Care providers provided “prompt medication reconciliation and periodic reassessments of a patient’s medical condition.”
  - The success of the Connected Care program may have much to do with its acknowledgement of the potential need to recalibrate patient-specific treatment plans with some regularity. As a result, would-be severe medical cases are caught before they progress to the point of requiring a hospital-level care.
- Prior to hospital discharge, physicians were alerted and performed an initial comprehensive visit generally on the date of SNF admission and always within 48 hours. The initial handoff was considered an imperative check point for care for this TOC model. There was acknowledgement that during the transition of care medication regimen errors or inaccurate assessments may occur. By performing “prompt medication reconciliation” Connected Care providers “recreated a vital element of successful outpatient readmissions prevention programs.”
- Acknowledgement that realistic patient goals are critical to reduce readmissions for poor prognosis or terminal patients
  - **“**In addition, Connected Care providers discussed goals of care—something that is often overlooked on admission to a SNF. This is particularly important because patients with chronic illnesses who are discharged to SNF often have poor prognoses. For example, Medicare patients with heart failure who are discharged to SNFs have 1-year mortality in excess of 50%. By implementing a plan of care consistent with patient and family goals, inappropriate readmissions for terminal patients may be avoided.”
  - Addition of palliative care specialist may have helped to make this goal of care discussion more meaningful for performance
- The Connected Care program SNFs promoted a culture of continuous improvement and were intentional about making course corrections at every level (i.e. medication administration, patient-physician communication, “goals-of care” identification, and treatment plan development) of care in order to reduce preventable readmissions. This is seen in the monthly meetings with root cause analysis and close communication about medication administration.

**Intervention 2. The REDE Model of Healthcare Communication: Optimizing Relationship as a Therapeutic Agent^70,89^**

***Intervention Background***

According to Windover et al,^89^ effective communication is the foundation for any relationship and does not preclude the healthcare setting. The delivery of high quality care requires this relationship to be “strong and meaningful” and for all clinicians to view patient relationship building as not only part of their role, but a necessary component of effective care delivery. To reinforce the premise that genuine relationships are a vital therapeutic agent, the REDE conceptual framework was conceptualized to teach and evaluating relationship-centered communication. This model is transformative because it challenges health providers to explore their own biases about patients and their role as clinicians; appreciates the skills “seasoned” practitioners already possess by providing a common langue that allows them to reflect and refine their pre-existing expertise; and characterizes communication skills as tools in a toolbox to be applied, as needed. Physician communication is a key component of the patient experience. According to Boissy et al 2016,^70^ it is the only metric that relates to physician care provision on Hospital Consumer Assessment of Healthcare Providers and Systems (HCAHPS) and Clinician and Group Consumer Assessment of Healthcare Providers and Systems (CGCAHPS) patient satisfaction scores. Provided these scores help shape reimbursement under value-based purchasing, physicians and hospitals are incentivized to improve them. To this end, the practical application of REDE model theory is examined as part of a Cleveland Clinic observational study that explores the impact of relationship-focused physician communication skills training on both patient satisfaction and physician experience.

***Intervention Details***

- The REDE model harnesses the power of relationships by configuring rich, empirically validated communication skills data into three primary phases of Relationship: Establishment, Development, and Engagement. Throughout each phase, the importance of dialogue is emphasized to ensure patients are engaged and involved in the learning process as well as their own treatment plans.
- The Cleveland Clinic Center for Excellence in Healthcare Communication developed an 8-hour experiential communication skills training called, “R.E.D.E (pronounced “ready”) to Communicate: Foundations of Healthcare Communication (FHC)”.
- Study participants in ancillary survey included attending physicians at the Cleveland Clinic who were mandated to attend R.E.D.E communication skills training for a 9-month period (August 1, 2013 – April 30, 2014) during regular working hours.
- FHC is based on the R.E.D.E. model, a conceptual framework for teaching and evaluating relationship-centered healthcare communication.
  - Each course was co-facilitated by two practicing clinicians trained in relationship-centered communication, adult learning theory, performance assessment, and group facilitation.
  - Groups contained no more than 12 participants who completed or participated in a series of interactive didactic presentations, live or video-based skill demonstrations, and small group skills practice sessions aligned with the three primary phases of the R.E.D.E. relationship model: Establishment, Development, and Engagement.
  - In Phase 1, the relationship between provider and patient is **established** through the adoption of the following practices:
    - *Opening an emotion bank account*, a concept originated by psychologist and author John Gottman, PhD, whereby a mental system for tracking the frequency with which we emotionally connect with other people is established.
    - *Conveying value and respect at the onset* of the relationship by building trust thereby initiating the development of emotion bank accounts with patients and their families.
    - *Setting the agenda collaboratively*, which research shows improves visit efficiency, diagnostic accuracy, and patient satisfaction.
    - *Purposefully introducing the computer* via electronic health records and using them to enhance, rather than detract, from patient care.
    - *Demonstrating empathy by using statements of empathy and taking care to not only recognize emotional cues, but to response to them with intentionality.*
  - In Phase 2, the provider-patient relationship is **developed** and fostered through:
    - *Reflective listening*, shown to enhance the therapeutic nature of a relationship, increase openness and the disclosure of feelings, and improve information recall.
    - *Elicit the patient narrative* by seeking to understand the patient’s perspective on his or her symptoms in lieu of obtaining the history of present illness (HPI) via a serious of closed-ended questions.
    - *Elicit the patient’s perspective* through curiosity coupled with a willing to learn, which is key to getting to know the patient and their illness.
  - In Phase 3, the provider **engages** patient relationships, which the researchers believe aligns with the education and treatment portion of the patient-provider interaction. In particular, the final phase points to the importance of:
    - *Sharing diagnosis* and ensuring the patient fully understands the information conveyed to him or her through context framing and by engaging in dialogue to answer clarifying questions that may arise.
    - *Developing a plan collaboratively* to support patient comprehension, decision making, and consideration of potential barriers to treatment. Research has shown that when the patient is included in the planning process, treatment adherence and behavior change is more likely.
    - *Providing closure* by respectfully reviewing and conveying appreciation for the time spent with the patient.
  - A final phase (Phase 3, detailed more above) of the FHC course integrated each phase of the R.E.D.E. model, focusing on communication challenges experienced in participants’ clinical practices.
- Physicians were asked to complete pre- and posts-course surveys on the date of training as well as 3 months post-completion of the FHC course. Surveys included demographic information, a communication skills self-assessment, knowledge and attitudes, the Jefferson Scale of Empathy (JSE), the Maslach Burnout Inventory (MBI), and post-course satisfaction.
- Physician information was also collected from a database maintained by researchers’ office of professional staff affairs. These data included gender, race/ethnicity, years in practice, specialty/subspecialty, and setting.

***Intervention Outcomes and Impacts***

- For physicians who took part in the R.E.D.E. communication skills training, adjusted overall CGCAHPS scores were higher than for controls (92.09 vs. 91.09, p <0.03). CGAHPS scores measure outpatient experience and may point to the R.E.D.E. communication skills training having a direct impact on outpatient experience.
- Significant improvement in the post-course HCAHPS Respect domain adjusted mean was seen in intervention versus control groups (91.08 vs. 88.79 respectively, p=0.02). The HCAHPS survey measures patients’ perspectives of hospital care. Improved scores indicate improved patient satisfaction with respect to scare in the hospital setting.
- Non-statistically significant improvements were also witnessed for adjusted HCAHPS communication score (83.95 vs. 82.73, p=0.22).
- Physicians reported high score satisfaction and showed significant improvement in empathy (116.4 ± 12.7 vs. 124 ± 11.9, p < 0.001). burnout, including all measures of emotional exhaustion, depersonalization, and personal accomplishment. The latter two measures were sustained for a period of at least 3 months.

***Intervention Facilitators***

- The REDE model builds on a significant research base including placebo, therapeutic alliance, communication skills and patient-centeredness that recognizes the healing potential of the healthcare relationship for not only patients but also providers.
- The REDE model helps frame the specific communication strategies that optimize their effect(s) on processes, outcomes of care and the patient-provider relationship itself.
- The REDE model also encapsulates evidence-based communication practices and our experience with seasoned clinicians, mostly staff physicians, within a large hospital system.
- “To date, more complex interventions and/or courses aimed at specific conditions have shown the greatest likelihood of improving patient experience. To our knowledge, this is the first study of a communication skills training intervention implemented for all physicians in a large multispecialty setting, and which uses CMS’s measures of patient experience.”
- The intervention incorporated conscientious steps to improve physician satisfaction and to convey to healthcare providers that they are valued and respected. This strategy in turn proved vital to improving patient-centered care.
- The same relationship building process imparted to participating physicians for use with patients was dually used to engage them. With this, physicians who attended skills training reported significant improvements in empathy and burnout.
- Limitations of the R.E.D.E. communication skills training intervention as expressed by researchers are detailed below:
  - Due to the observational nature of the study, other causes for improvement in CGCAHPS, HCAHPS, and other metrics (i.e., empathy and burnout) could not be ruled out. Researchers attempted to control for secular trends in patient experience by including a control group and adjusting for confounders.
  - Some non-anonymous self-reporting was included in the study model, which may have contributed to some physician participants exhibiting a social desirability bias in their responses. Researchers believe this possibility to be unlikely, as participants demonstrated no hesitance to express their skepticism about the FHC course.
  - Generalizability of the study may be an issue, as not all organizations can mandate training. However, researchers are keen to express that this study has important implications nonetheless, as it can serve as a model for other entities considering similar communication skills driven initiatives.

**Intervention 3. Mobile Stroke Treatment Unit (MSTU) with Telemedicine Capabilities^90^**

***Intervention Background***

Timely thrombolysis, a procedure used to break up abnormal blood clots that restrict blood flow in veins and arteries, for acute ischemic stroke is associated with a greater chance for improved outcomes. Patients treated within 0 to 1.5 hours following symptom onset have the best outcome with a number (of blood clots) needed to treat (NNT) of 4.5. NNT nearly doubles every ninety (90) minutes post-symptom onset. As the number of blood clots requiring treatment increases, the chance of a favorable outcome decreases. The time-sensitive nature of stroke treatment has fostered new approaches to shorten time to treatment. Public stroke education campaigns, prenotification by emergency medical services (EMS) to receiving hospitals, and point-of-care laboratory testing have resulted in notable reductions in the onset-to-treatment time. However, these strategies have not addressed thrombolysis in the pre-hospital setting. To this end, the Cleveland Clinic has implemented a telemedicine-enabled mobile stroke treatment unit (MSTU) to reduce the time to evaluation and treatment of thrombolysis patients—a model that is contrasted with use of a traditional ambulance in a U.S. urban environment.

***Intervention Details***

- The Cleveland Clinic mobile stroke treatment unit (MSTU) program was planned and executed in partnership with the City of Cleveland, Cleveland EMS and partner hospitals in Cleveland, Ohio. It became operational in July 2014 and was active 12 hours per day from 8am to 8pm, 7 days a week.
- MSTU home base was located approximately 4 miles east of central downtown Cleveland.
  - Transfer sites for the MSTU program included 3 comprehensive stroke center hospitals and 11 primary stroke centers or stroke-certified hospitals.
  - The Cleveland EMS dispatch center simultaneously deployed both EMS and MSTU crews for every potential stroke call. EMS generally arrives first: “The MSTU paramedics go to the scene and receive handoff from the Cleveland EMS paramedics.”
- The MSTU medical team included:
  - A vascular neurologist who examines the patient via telemedicine and instructs the MSTU crew.
  - A registered nurse who assists the physician in performing the neurologic examination
  - A MSTU paramedic who goes to the scene for patient transfer from Cleveland EMS paramedics; an emergency medical technician (EMT).
  - ACT technologist cross-trained as an EMT.
- The following equipment and technology was used by the MSTU team:
  - A mobile CT scanner (Ceretom, NeuroLogica Corporation, Danvers, MA),
    - CT images were transmitted to an offsite neuroradiologist for interpretation.
  - Telemedicine capabilities included:
    - Network (long-term evolution; Verizon Wireless, Basking Ridge, NJ) to transfer radiologic and telemedicine images.
    - Monitor to display radiologic and telemedicine images.
    - Remote control operated, high-resolution medical-grade digital camera (Rp-Xpress; InTouch Health, Goleta, CA).
    - Portable point-of-care laboratory equipment to allow clinical staff to achieve real-time, lab-quality diagnostic results.
- The EMS dispatch center used the standardized national medical priority dispatch code, “28-C”, to broadly indicate an in-progress stroke or ischemic attack.
- City EMS performed the initial patient evaluation, which included the Cincinnati Prehospital Stroke Scale.
  - If stroke was suspected, City EMS would transition care to the MSTU team.
  - “For patients who are deemed not to have had a stroke by the Cleveland EMS, the MSTU activation is cancelled.”
- MSTU patients receive immediate assessment, vital sign monitoring, a head CT, laboratory studies and intravenous access (IV) to collect necessary blood samples to run point-of-care testing by MSTU clinical staff. The CT technologist then registers the patient into the hospital electronic medical record (EMR). This EMR can be viewed from any hospitals within the Cleveland Clinic system. A NIH Stroke Scale (NIHSS) is then performed by the vascular neurologist via telemedicine.

***Intervention Outcomes and Impacts***

- Of the first 100 MSTU patients, 16 received thrombolysis.
  - Among patients who received a preliminary diagnosis of probable stroke, the thrombolysis rate was found to be 48% (16/33).
  - “Sixteen patients evaluated on MSTU received thrombolysis, 25% of whom received it within 60 minutes of symptom onset.”
- Patient and stroke severity characteristics were similar between 100 MSTU and 53 ED control patients (initial NIH Stroke Scale score 6 vs 7, p = 0.679).
- “The MSTU took a median time of 12 minutes (IQR 8–14) to reach its destination from the dispatch call time (alarm).”
- “Patients entered the MSTU door in a median time of 20 minutes (IQR 16–24) from the alarm time.”
- “The median MSTU duration of use for each case was 86 minutes (IQR 78–94).”
- There was a significant reduction in median alarm-to-CT scan completion times (33 minutes for MSTU patients versus 94 minutes for the controls, p < 0.0001).
- MSTU program patients received thrombolysis significantly sooner than control patients according to three metrics
  - 38.5 minutes sooner from the alarm time (median 55.5 [IQR 24-47] minutes compared to 94 [IQR 78-104] minutes, p <0.0001).
    - Researchers point to a telemedicine-enabled, dedicated stroke ambulance equipped with a CT scanner as the main driver of a near 40-minute faster delivery of thrombolysis when compared to conventional stroke care delivery.
    - This finding is supported by the first pilot randomized controlled trial (RCT) conducted on 100 patients by researchers from University of Saarland, Germany, which achieved a 34-minute reduction in alarm-to-thrombolysis with ambulance-based thrombolysis.
  - 26.5 minutes sooner from door (median 31.5 minutes MSTU vs 58 minutes controls, p = 0.0012),
  - 25.5 minutes sooner from symptom onset median 97 [IQR 61–144] minutes compared to 122.5 [110–176] minutes, p = 0.0485)

***Intervention Facilitators***

- The Cleveland EMS dispatch center simultaneously dispatched both an EMS and MSTU crew for every potential stroke call, which may have helped to reduce the response time from alarm-to-thrombolysis.
- The MSTU program possessed a fast-wireless network with reliable coverage, which likely helped to sustain the telemedicine component of the intervention and improve its time-to-treatment outcomes.
  - “In cities possessing a fast-wireless network with reliable coverage over the MSTU deployment area, this result can be achieved by solely using teleradiology and telemedicine.”
- “A key difference in our MSTU model [versus others developed elsewhere] is its sole use of telemedicine and teleradiology, which may be a more practical strategy economically, while enabling a physician to cover multiple tasks potentially, multiple MSTU units in the future.”
  - The cost-effectiveness of the mobile stroke unit is supported by Dietrich et al. 2014, which found prehospital treatment of acute stroke to be highly cost effective across a wide range of scenarios. The study points to the optimization of its cost-effectiveness through the reduction of MSTU staff size.
  - “Our approach is to create a cost-effective model by using telemedicine and teleradiology on multiple MSTUs to broaden the reach of a single stroke neurologist and neuroradiologist.”
- The local EMS stroke training protocol was changed to note that after obtaining a brief history and assessing for immediate life threats, care for positive cases on the Cincinnati stroke scale were to be handed over to MSTU clinical staff. This procedural modification facilitated immediate transfer of high risk patients to the MSTU program to reduce alarm-to-treatment times for thrombolysis.
- Researchers argue that prehospital administration of IV tPA, the only approved treatment for patients with acute ischemic stroke, is beneficial because patients receive thrombolysis more quickly, which may translate into improved outcomes.
- The application of the MSTU program in other areas could be an issue, as the model is dependent upon fast and reliable wireless networks for timely transmission of CT images to an offsite neuroradiologist for interpretation and to provide an overall, high quality telemedicine encounter. Furthermore, geographic layouts vary by region and could serve to hinder the effectiveness of the MSTU program in areas dealing with land specific barriers.
  - “The feasibility of MSTU in other areas also remains a question, as it is dependent on reliable and fast wireless networks to allow for fast transmission of CT images and provide a high-quality telemedicine encounter”
- Radical step of bringing care to the patient represented the greatest facilitator of positive outcomes – it was not necessarily that patients interfaced with providers sooner, but that delays that come from the hospital care processes were cut out, allowing faster time to treatment.
  - “From the onset of symptoms, patients entered the MSTU door at a similar time compared to when control patients entered the ED door (52.5 minutes vs 64 minutes, p 5 0.3)”
  - “Following arrival at the hospital, there are more delays and in actual clinical practice only 11.3% of patients are able to receive IV thrombolysis within 1.5 hours of symptom onset [Saver 2015]. With the utilization of the MSTU, we were able to demonstrate a significant reduction of 25.5 minutes in onset-to-treatment time compared to conventional stroke care deliver”
  - “Each link in the prehospital stroke rescue chain matters, and mobile stroke units appear to have the greatest potential in accelerating the process to IV thrombolysis and delivery to definitive care”

Case Study 4. Mayo clinic

**Institutional Profile**

***Institutional Mission^91,92^*** **–** “To inspire hope and contribute to health and well-being by providing the best care to every patient through integrated clinical practice, education and research.”

***Institutional Vision*^91^** *–***-** “Mayo Clinic will provide an unparalleled experience as the most trusted partner for health care.”

***Institutional Primary Value Statement*^91^** – “The needs of the patient come first.”

***Institutional Values^91^*** *–* Respect, Integrity, Compassion, Healing, Teamwork, Innovation, Excellence

***Key Facts*** *–* The Mayo Clinic is highly regarded as the world’s oldest integrated multispecialty not-for-profit medical group and has evolved into one of the world’s leading nonprofit medical organization with academic, research and clinical practice through an open, integrated model at regional, national and international levels to benefit all types of patient care.^92^ Headquartered in Rochester, Minnesota at their flagship campus, they also have large campuses in Arizona and Florida. In total Mayo operates at least 22 hospitals and 76 outpatient facilities^93^ and serves 1.2M patients annually.^94^ In addition, Mayo Clinic owns a regional health network called the “Mayo Clinic Health System” which covers 15 counties across Minnesota, Wisconsin, and Iowa that served 110,431 patients in 2017. In this network, Mayo directly operates 10 outpatient clinics and 2 hospitals while partnering with 18 other hospitals and 65 clinics.^95^ In 2019, they reported over $12.6 billion in revenue and employ approximately 65,000 employees, of which 4,800 are physicians and scientists and 1,770 are residents.^94^ Their flagship campus accounts for more than half of these employees, staffing 36,330 with 2,543 physicians.^94^ Inpatient admissions can be estimated at approximately 123,000 or more based on available figures,^93^ and, in 2019, 130,000 people were reported to undergo surgery.^94^

Of note, the Mayo Clinic considers its three campuses “destination medical centers”, reporting patients arriving from 138 countries with 33% of patients having complex or highly complex medical conditions. They identify four areas of highly specialized and sought-after care: cancer care with 120,000 patients annually; adult and pediatric cardiovascular care with 100,000 patients annually; adult and pediatric neurological care with 100,000 patients annually; and, lastly, transplantation services with more than 150 surgeons and physicians dedicated to these patients. Mayo Clinic reports 88% of patients seeking a second opinion received a refined or new diagnosis.^94^

***Characteristics*** *–* Mayo Clinic’s care delivery model is based on an open, integrated health system with three campuses as well as two health networks: The Mayo Clinic Health System and The Mayo Clinic Care Network. A physician-led management structure helps develop a culture at Mayo that filters all decision making, at all levels of the organization, through the lens of best serving the patient.^92^ Mayo Clinic does not offer in-house insurance with the exception of a set of employee policies administered by Minneapolis-based insurer, Medica, and a number of commercial and individual accountable care organization (ACO) products for Southeastern Minnesota and Southwestern Wisconsin.^96^ Our peer-reviewed literature search garnered an article published by Mayo affiliates dedicated to explaining their care philosophy in detail. One of the key tenets regards the idea of “union of force”, which is an elaboration on “teamwork”: “Mayo Clinic personnel work collaboratively in teams within and across all departments to meet the […] needs of patients. All Mayo personnel are expected to contribute to a learning organization environment that is committed to providing the best outcomes, service, and value in the delivery of health care.” ^97^ Stemming from this core value, Mayo Clinic owes its success to a number of key attributes. A multidisciplinary practice that is coupled with salaried health professional staff naturally make for patient-based care and provider accountability. Supportive organizational structure and integrated EHR and other technologies frees up provider time and energy, allowing greater focus on patients.^92^ Major decisions are made at a committee level, which although taking more time than a top-down approach, facilitates buy-in at every level. To deliver seamless care at and between institutions, Mayo adopted a universal EHR, accessible by clinicians at any site, as well as Clinic-wide telephonic paging.^98^

Mayo has also made a push for the use of technology to improve patient care specifically with regard to knowledge management. Importantly, the knowledge management system was conceived as a result of asking the question “What does Mayo know that it knows?” which began the process of formalizing Mayo expertise through Knowledge Committees that develop topics of interest and continuously review to ensure all information is accurate and recent. After IT development to support feasibility and clinical integration within their EHR, their advanced knowledge management system stored essential information developed by the knowledge committees as “kernels” from which different interfaces can pull information. The system currently holds information for over 1,500 topics. The interfaces include provider facing clinical process flow charts, clinical decision support tools, and “AskMayoExpert” information articles. All of these interfaces are available to Mayo and Mayo-affiliated physicians directly through the EHR to limited managing between different applications. Patient- and family- facing materials also stem from the storage kernel with education materials for conditions.^99-100^ Mayo has also made it a priority to be at the forefront of new telemedicine trends and the delivery of virtual services, even establishing a Center for Connected Care which manages 54 telemedicine initiatives and develops strategy for their implementation.^101^ Together, their EHR-integrated knowledge management system and telemedicine infrastructure allow them to develop the Mayo Care Network, which is both a national and global service providing access to Mayo expertise and related clinical guidelines through the dual action of EHR clinical decision support tools and telemedicine consultation.^102^

***Trends in Patient-Centered Care and Care Coordination*** *–* In our review of Mayo Clinic’s peer-reviewed patient-centered care and care coordination peer review articles, which was sizable, there were three overall themes: physical colocation of specialists to routine care teams,^103-5^ assessment of the impact of e-consultation and e-visits on patient access or experience,^106-109^ and care standardization supported by knowledge management informatics^110-111^ or EHR-based support/reinforcement.^112^ Assessments of technology and telehealth interventions included smaller scale and larger scale efforts. Smaller scale efforts included problem-specific patient-facing apps,^113-4^ scheduling algorithms,^115^ and waiting room pagers.^116^ Larger scale efforts involved assessments of telespecialty lines, particularly telestroke,^117^ and evaluation of telemedical equipment to support service line goals.^118-20^ Lastly, Mayo put at least a decade of effort into researching Shared Decision Aids (SDAs), or Decision Aids (DAs), on the premise that this would facilitate knowledge transfer to patients, improve patient engagement as a part of their own medical team and reduce decision conflict. The research showed some positive benefit, as in the PCI Choice DA whose RCT showed increase in patient knowledge but no reduction in decisional conflict.^121^ Additionally, there was one recent RCT with stronger evidence to support adoption of an antidepressant DA.^122^ However, on the whole the body of evidence was considered largely inadequate by Mayo personnel,^123^ and in utilization analyses was found to be unpopular with physicians because they were considered burdensome and unhelpful.^124^ In the end, the research and subanalyses suggest rather that patient engagement and reduction of decisional conflict would be facilitated by improving the relational quality and physician-patient joint identification of care goals, values and lifestyle considerations.^123^ This has led Mayo to shift gears in the types of patient engagement aids to develop and research, as we see recently in 2019 with the development of a “conversation aid” called ICAN.^125-6^ While promising, the first randomized control trial has not yet been published. The 2019 pilot demonstrates feasibility but was not found to improve 6-month medical adherence nor increase the length of patient-provider visits in a small group of patients.^126^

***Comment on Mayo Interventions Selected*** *–* Interventions selected include (1) a teleneonatology service line, (2) a neurologist embedded in a primary care clinic, and (3) a “Mayo Expert Advisor” clinical decision support tool. These interventions were selected after review of 92 peer-review articles featuring studies taking place at Mayo Clinic facilities that were published by or affiliated with Mayo Clinic personnel.

**Intervention 1. Teleneonatology for Newborn Resuscitation^127-9^**

***Intervention Background***

Telemedicine initiatives can address many issues in health care quality and delivery, not least of which is rural-urban disparity in quality. Researchers decided to explore the application of telemedicine to neonatology based on promising iterations at other institutions that suggest improvement to disparities in neonatal care. From March 26, 2013 to December 1, 2015, the Division of Neonatal Medicine at the Mayo Clinic Health System, with support of the Center for Connected Care, whose guiding directive is to improve telemedicine infrastructure, provided video telemedicine consults for newborn resuscitations to six different Mayo Health system community sites, ranging from 40–120 miles from the main campus in Rochester, MN. As part of an effort to improve performance and enhance the implementation of the intervention, provider opinions and process measures on a number of factors were collected, such as the technical quality of the video conferencing software, connection reliability, the quality of teamwork between the referring and consulting providers, and whether or not the quality of care for the infants was improved. Quality measures were also evaluated for patients who were later admitted to the Mayo Clinic Hospital NICU by an impartial expert panel and compared between patients who previously received teleneonatology consults and those who did not.

***Intervention Details***

- Synchronous video telemedicine consults for newborn resuscitations between main campus and six Mayo Health system community sites 40-120mi away (community sites were a mix of level I and level II nurseries, with teleneonatologist affiliated with a level IV center with approximately 350 admissions annually).
- In sum, the neonatologist was able to assist with patient-family communication, advanced resuscitation, respiratory management, coaching local team through CPR, and conducting neurologic assessments.
- Provider in community hospital identifies case in need of intervention, calls a triage line and neonatologist is paged.
  - “The Mayo Clinic Division of Neonatal Medicine did not mandate specific activation criteria. The local care teams were advised to use teleneonatology when they believed it was clinically indicated and were encouraged to activate the service early in the clinical course.”
  - Triage line is run by Mayo’s institutional admission and transfer center (ATC).
- Community provider and neonatologist discuss case on the phone prior to initiating video.
- The neonatologist was provided visualization of the patient via the video link and then conferred with the referring provider to come to an appropriate plan.
- Video was provided to community providers through HIPAA compliant software on a consumer-grade wireless tablet device; consulting neonatologists used a variety of wireless and wired options and were able to provide consultations on the Mayo campus and from home.
- “During these consultations, the neonatologist actively guided the multiple, critical steps involved resuscitating and stabilizing infants. The consulting neonatologist was also able to communicate with the parents about the status of their infant before transportation. In cases of severe respiratory distress and respiratory failure, the consulting neonatologist assisted the local provider with advanced respiratory management.”
- “For infants who required CPR, the neonatologist coached the local team to provide effective respirations and coordinated chest compressions. Importantly, the neonatologist was also able to perform a neurologic assessment of the newborn to determine whether the patient qualified for therapeutic hypothermia. For infants born with a previously undiagnosed congenital anomaly, the neonatologist was able to diagnose the defect, guide short-term management, and notify the correct pediatric surgical subspecialists.”
- “Before implementation, the neonatologists (J.L.F. and C.E.C.) made one or two site visits to provide an overview of teleneonatology and introduce the local providers to telemedicine technology.”
- As part of process measures to assess quality and use of the intervention, log of all telemedicine consults was maintained, collecting patient disposition, referring site, local provider, and consulting neonatologist. Video consult duration was also collected.
- As part of quality improvement, surveys were sent to local and neonatologist providers after each consult.

***Intervention Outcomes and Impacts***

- 84 consults were conducted within 6 study sites in the study period. Top reasons for consult are: (1) prematurity (n=32), defined as gestational age <37 weeks; (2) respiratory distress (n=15); need/anticipated need for advanced resuscitation (n=14); congenital anomaly (n=8).
- After consultation, 27 infants (32.1%) were able to remain at the community hospital, and 56 (66.7%) were ultimately moved to a NICU. One patient died. “If the remote neonatologist had not been able to visually assess the newborn and provide remote-guided care, many of these infants may have otherwise been transferred to the NICU unnecessarily.”
- In 93.3% of surveyed cases (14/15), local providers felt that patient care and outcomes were improved, with 93.8% of local providers willing to use telemedicine again and would recommend it to others.
- In a case control study, 47 neonates were classed into the teleneonatology group and were matched to controls that did not receive telemedicine services (i.e. teleneonatology consult); if local providers communicated with the neonatologist it was by telephone only. The telemedicine group was significantly more likely than their matched control to undergo measurement of temperature (79% vs 55%, P=.02), blood glucose (94% vs 81%, P=.03), and blood gas (49% vs 28%, P=.008).
- Patients in the teleneonatology group were also significantly more likely to have all three values measured (temperature, glucose, blood gas) during the resuscitation (43% vs 21%, P=.008).
- Median resuscitation quality of those in telemedicine group rated out of 10 is a 7. Median resuscitation quality of those in matched control is a 4. Resuscitation quality was evaluated by a two-person expert panel on a 1-10 descriptive scale.
- When analyzing 35 matched pairs that had a consult within one hour of birth, the positive impact of teleneonatology was greater (median rating 8 vs 4, median difference 2, P=.003).
- Subgroup analysis demonstrated teleneonatology significantly improved the resuscitation of preterm neonates (median rating 8 vs 4, median difference 1.5, P=.004).
- Median wait time for connection to neonatologist was 13.1 min.
- Overall median consult call duration was 14.5 min; median consult call duration was 3 min for neonates not requiring transfer while the median consult call duration was 30 min for neonates requiring transfer.
- “With the process and technology used in this study, the mean turnaround time from telephone call to video connection was approximately 9 minutes, with clinically significant undesirable variation. Because these are emergency consults, a consistent turnaround time of less than 5 minutes is required. Process improvements are currently under way to address this issue, for example, call triage algorithms and electronic forms to facilitate text paging.”
- “Because of the poor reliability and audio-video quality of the technology used in this study, Mayo Clinic has transitioned the emergency telemedicine platform for newborn resuscitations to a wired solution. Local teams now use a wired telemedicine cart that is equipped with a high-definition camera with pan/tilt/zoom capabilities that can be remotely controlled by the neonatologist.”

***Intervention Facilitators***

- Telemedicine initiatives are supported by a centralized Mayo Center for Connected Care, whose guiding directive is to improve telemedicine infrastructure.
- Supportive research from inception including analysis of technical roadblocks like ease of use, quality of virtual communication, and network reliability to inform rapid process improvements and an explicit evaluation of facilitators and barriers in a post-implementation mixed-methods study.
- “The value created by the video telemedicine consult is contingent on a local team that is Neonatal Resuscitation Program trained and has the necessary skills and equipment needed for newborn resuscitation. Implementation of the telemedicine program has been complemented by outreach education to the health system sites.”
- A highly reliable, secure, and supported technology infrastructure that provides high-quality audio and video should be considered for any emergency video telemedicine consultations.
- Mixed-methods findings detailed potential facilitators for future follow up/implementation of telemedicine project based on teleneonatology findings:
  - Reliable and simple IT infrastructure to reduce workload burden and to remove “background fear” that technology issues would make communication unreliable (drops in connectivity, device failure, etc.).
  - Education for staff on the value and purpose of teleneonatology prior to implementation, as well as ongoing education about the service.
  - “Continuous assurance to local physicians that competencies are not being assessed.”
  - Involving site personnel during the implementation process (i.e., include key local stakeholders in meetings, workflow design, and training and education.
  - Relationship-building between hub and spoke site personnel may encourage successful implementation.
  - All parties know indications for activation of intervention in emergency setting.
    - Modeling an emergency response (e.g., an immediate connection with the telemedicine consultant)
    - Co-develop (local providers and consultants) clear guidelines for use of the new telemedicine service (i.e., indications for service activation and timing of the call and before vs. after delivery).
    - Education on anticipation of a neonatal emergency and recommendations that the technology be available at the bedside if any clinical concerns arise.
    - Designing clear clinical workflows with designated persons to call the consult and to retrieve the telemedicine technology.

**Intervention 2. Integrated, Co-located Neurology in a Primary Care Medical Home^105^**

***Intervention Background***

Innovative care delivery models are required to reduce health care utilization and costs while still maintaining excellent quality and safety of care. One such model is colocation of specialists in primary care patient homes (PCMHs), coupled with the usage of electronic and/or curbside consultations. Traditionally, the usage of electronic or curbside consultations in the inpatient setting were viewed with some skepticism in terms of their safety and efficacy when compared to face to face consultations because of the lack of ability of the consulting specialist to verify patient presentation. However, they remain valuable and efficient tools to improve patient centered care, though they require more validation.

Researchers at the Mayo Clinic explored the integration of collocated neurologists (called ICS neurologists, for *integrated, collocated* neurologists) with curbside and electronic consultations, seeking to understand the effects on health care resource use of testing and referrals to face-to-face neurologist care, as well as any adverse outcomes of electronic and curbside consultations as compared to the typical face-to-face consultation. Secondary goals were to enhance the care coordination between primary care and neurology providers, increase responsiveness of neurology consultations if required, and maintaining continuity of care within the PCMH. “Prior to model implementation, neurology consultation and longitudinal care was provided by the Mayo Clinic tertiary referral neurology practice through referrals placed in the EHR computerized physician order entry system.”

The colocation model was implemented at Mayo Clinic Employee and Community Health, a vertical, multisite PCMH serving approximately 140,000 patients residing predominantly in Rochester, MN and seven surrounding counties. Half of the patient base are Mayo Clinic employees and dependents, and as such are insured through Mayo Clinic. On October 1, 2014, Employee Community Health debuted the collocated neurologist service, and from December 1, 2014 to March 13, 2015, consecutive patients were included in the analysis about interaction with a primary care clinician and collocated neurologist. A retrospective chart review was later conducted to “determine the number of diagnostic tests and face‐to‐face visits that were still avoided. The chart review also determined if adverse outcomes were associated with non‐face‐to‐face consultation and test avoidance.”

***Intervention Details***

- “Promotion of ICS neurologists within the practice was conducted via physician‐led staff meetings, emails, electronic newsletter announcements, and a display in every examination room of […] contact information, including photograph and pager number.”
- “Direct PCP referrals to the tertiary neurology practice for face‐to‐face visits were not permitted without prior collaborative consultation” with the collocated neurologist, facilitated by the alteration of the EHR.
- 0.6 FTE collocated general neurologist at the main practice site of PCMH, with partial support of 3 FTE RNs and 3 FTE clinical assistants.
- “During each one‐half day, 2 to 3, 45‐minute, new patient face‐to‐face and 1, 30‐minute follow‐up face‐to‐face appointments were available for prescheduling.”
- “… unscheduled time was used to provide curbside, electronic, and same day face-to-face consults, as well as time for follow-up on EHR-generated patient messages, test results, and communication with referring PCPs.”
- Three types of consultations were provided:
  - **Curbside consult:** “Informal consultations defined as a phone or electronic communication within the electronic medical record or via email between the neurologist and PCP. These were provided by a single neurologist, were available to PCPs before ordering a face‐to‐face consultation, and included an opportunity to review of the EHR at the discretion of the specialist. Curbside consultations were documented in the EHR at the discretion of the referring provider. Curbside consultations were available when the neurologist was not physically present in the primary care practice. A curbside consultation was not required prior to ordering other types of consultations.”
  - **E‐consult: “**Initiated by an electronic order by the referring provider resulting in an EHR review and documentation of clinical note by the specialist that included the disclaimer: ‘This patient was not personally interviewed or examined. The history and examination findings are based on the clinical documentation provided and/or discussion with a physician or provider who had personally interviewed and examined the patient.’”
  - **Face‐to‐face consult**: “Involved a complete neurological history and examination documented in standard fashion in the EHR conducted by the co-located neurologist.”
- Beginning December 1, 2014 and ending March 13, 2015, prospective data was on consecutive consultation referrals to the collocated neurologist were collected:
  - Provider type
  - Consult type (curbside, e-consult, face-to-face)
  - “Face‐to‐face consult and diagnostic testing considered by the neurologist or PCP but not ordered at time of initial consultation (any type) were also recorded.”
  - “The number of face‐to‐face visits that could have been completed via curbside or e‐consults over the data collection period was annualized as was as the potential impact of an earlier face‐to‐face visit on diagnostic test use.
  - “Adverse events were defined as a delayed testing, diagnosis, or intervention for a life‐threatening or disabling condition related to curbside, e‐consult, or specialist recommendation deferring testing or face‐to‐face neurologic consultation.”
  - “Medication management errors leading to harm such as a fall, seizure, emergency department presentation, or other injury were also included.”
- “A follow‐up, retrospective chart review was performed after at least 4 months after last consultation to determine the number of diagnostic tests and face‐to‐face visits that were still avoided.”
  - Chart review also determined if adverse outcomes were associated with non-face-to-face consultation and test avoidance.”

***Intervention Outcomes and Impacts***

- Consult referring parties:
  - “Consults were primarily initiated by physicians (64%), followed by NP and PA providers (23%), family and internal medicine resident trainees (9%), and other specialists (4%).”
- Neurologist visit types:
  - “Of the 182 face‐to‐face visits, 57 were performed after a curbside consult, and 11 were performed after an e‐consult at the discretion of the [collocated] neurologist. Five patients who did not show for a scheduled face‐to‐face visit were converted into e‐consults.”
  - “Among curbside consults, 59 (33%) resulted in agreement with the PCP outlined care plan without the need for additional diagnosis testing or face‐to‐face consult. In approximately one‐quarter of curbside consults, the ICS neurologists recommended that a patient be seen in face‐to‐face consultation within the same half day of the primary care evaluation (18, 10%) or rescheduled to be seen sooner than the next routinely available appointment (25, 14%). The neurologist converted 12 (7%) curbsides to an e‐consult to serve as a reminder of complex recommendations for all members of the care team. Two (1%) patients were triaged by the ICS neurologist directly to an e‐consult and 4 patients (2%) to a tertiary neurology face‐to‐face consult.”
- “We observed that a model of co-located, integrated neurology in a PCMH resulted in avoidance of diagnostic tests at a median 6‐month follow‐up and decreased PCP referrals for face‐to‐face consults to both integrated and tertiary neurology.”
  - “Retrospectively, diagnostic testing or a face‐to‐face visit may have been avoided in 40 (22%) patients if a curbside was obtained earlier in the care planning process.”
  - Similarly, an earlier face‐to‐face consult was estimated to have avoided testing in 31 (17%) patients including brain magnetic resonance imaging (MRI) (n = 29), electromyogram (n = 10), lumbar MRI (n = 7), noninvasive angiography of head or neck (n = 5), spinal MRI (n = 4), and autonomic testing (n = 4).”
  - The number of referrals for face‐to‐face consults to both ICS‐Neurology and the tertiary neurology practice declined by an average of 25% compared to the 3 months prior to model implementation. The number of referrals for face‐to‐face consults to the tertiary neurology declined by 64%.”
- “Overall, the integrated neurology model appears to foster efficient, collaborative care that leverages closer relationships with PCPs and understanding of the specialty care needs of the community‐based population within the PCMH.”
  - PCPs may have felt “more comfortable reaching out to develop care plans with neurology earlier than in a traditional model of care.”
  - “There may also be a benefit of early face‐to‐face consultation measured by avoidance of unnecessary diagnostic testing if a specialist is able to provide a timely and accurate clinical diagnosis.”
  - Integrated neurology may also facilitate effective knowledge exchange about neurologic disease evaluation and management and aid in developing a refined approach to testing and management over time, leading to higher value care delivered overall.”
- No adverse outcomes were observed associated with the use of curbside consultations, but authors caution that risk of curbside consultations and relying on secondhand patient histories might be underestimated
  - The authors note that in their study that risk was likely mitigated by the availability and use of a shared EHR.

***Intervention Facilitators***

- Since the patient base was largely insured by Mayo, the implementation of curbside or e-visit consultations was viable because of financial incentives to lower care costs while still improving care quality in subspecialty referral.
  - “We assumed that use [of diagnostic tests and face-to-face consultations] in a fee-for-service–driven model using subspecialists would have been higher and that face‐to‐face consultation would be inherently lower risk to patients than delivering care with electronic or curbside consultations.”
  - “The generalizability of this model is reduced because there is limited or no reimbursement for curbside and electronic consultations in the current health care marketplace.”
- PCP and neurologist were both salaried, potentially encouraging unscheduled time and non-billable labor.
- Shared, robust and adaptable EHR system at the implementation site (and the Mayo Health system as a whole) allowed effective capture and use of patient data: as the researchers point out, “Mayo Clinic is a vertically integrated, tertiary referral, multispecialty group practice with a shared electronic health record (EHR) that allows tracking of laboratory results, diagnosis, and hospitalizations as well as provider and patient communication.”
- Use of clinical history of referring provider alongside integrated EHR empowered specialists to reach out to patients if they needed more information, increasing accuracy: “Unlike previous studies on curbside consultations, we used an integrated EHR in conjunction with the clinical history as presented by the referring provider. This model also allowed for flexibility to call patients to obtain more clinical history, decide if a face‐to‐face neurologic examination was needed, and expedite diagnostic testing and treatment. These differences likely allowed us to increase accuracy of information beyond that of previous investigations.”
- Co-location of the specialist within the primary care practice may have helped facilitate PCP comfort with earlier neurology intervention: “Through co‐location, PCPs may feel more comfortable reaching out to develop care plans with neurology earlier than in a traditional model of care.”
- Requirement that PCP collaboratively consulted with ICS neurologist prior to referral to the tertiary neurology practice was facilitated by “alteration of the EHR ordering system screen that routed all PCP referrals [such that] any face‐to‐face neurology consults [would be routed] to ICS‐Neurology” first.

**Intervention 3. Clinical Decision Support Tool to Improve Efficiency and Accuracy of Recommendations for Cholesterol Management^110^**

***Intervention Background***

Best practice protocol in prevention and treatment of atherosclerotic cardiovascular disease (ASCVD) involves calculation of a cardiovascular risk score, usually on online calculators, that require physician input of data such “age, sex, race, blood pressure, hypertension history, smoking status, and cholesterol level.” However, current guideline compliance is suboptimal for a number of reasons. Literature has suggested that there is inadequate time in a visit to gather the data required for risk calculation. Additionally, the unwieldiness of EHR systems impose high time and click burdens on physicians because they contain “an overwhelming amount of information…not necessarily optimized for clinician workflow.” This also increases the likelihood of clinical errors.

Researchers at Mayo Clinic developed a clinical decision support tool, MayoExpertAdvisor (MEA), to help automate the process of calculating risk of and providing guideline-based recommended treatments for ASCVD. The novel components of MEA were that it used “natural language processing (NLP)” and it was “the first to present treatment recommendations along supporting data and links to educational resources and decision aids.”

The impact of the tool on efficiency and accuracy of risk calculation and recommendation of treatments was evaluated via simulation of patient treatment using real patient data; invited physicians used retrospective patient data to calculate risk scores and recommend treatment, with or without MEA support. Clinician satisfaction with the tool was later gauged via survey.

***Intervention Details***

- Clinicians were invited to participate from Employee and Community Health and Cardiology, a patient centered medical home in Mayo Clinic, Rochester, MN.
  - “Thirty-three clinicians of the eligible 262 including 13 primary care internal medicine physicians, 4 family medicine physicians, 10 internal medicine residents, and 6 cardiology nurse practitioners participated in the study.”
- Real patient information was used to simulate MEA use in clinical practice. Patients were selected from the Employee and Community health pool who received primary care at Mayo Clinic, Rochester, MN.
  - “We randomly selected patients who were not on the recommended dose of statin, determined by manual review. We looked at two groups of patients. The first group included 409 patients between the ages of 40 and 75 with an LDL greater than 190mg/dL and no history of ASCVD. The second group included 3880 diabetic patients between the ages of 40 and 75 with an LDL over 70 mg/dL or Non-HDL over 100 and no history of ASCVD. We selected four patients randomly from each group, and these 8 patients were used for the test scenarios in the study.”
- “The clinicians were asked to calculate the ASCVD risk score and recommend treatment for each patient based on EHR review with or without input from MEA. The patient group for which MEA input was given was switched every other clinician so that time differences could not be attributed to different patient characteristics.”
- Clinical operationalization of the MEA is as follows:
  - “MEA provides care recommendations that are established by care process models (CPMs) in Ask-MayoExpert, an internal resource designed for point of care knowledge delivery. The knowledge of the CPMs is based on the most recent guidelines and input from subject matter experts. The CPMs are presented as flow-charts so that clinicians may proceed through each decision point until they arrive at the appropriate care recommendation. Embedded within the CPMs are risk score calculators and links to relevant patient education materials.”
  - “The system delivers individualized care recommendations based on patient-specific data from the EHR and the knowledge from the CPMs. In addition to the recommended clinical action, the user interface also delivers automated risk score calculations and access to relevant patient education materials and shared decision-making tools.”
  - “MEA is integrated into the locally produced EHR in order to limit the number of applications that clinicians need to open during patient encounters.”
- MEA technical architecture:
  - “The clinical data needed to automate the care processes come from diverse sources including multiple EHR and internal specialty specific applications. Both structured data and unstructured data concepts are extracted from the clinical notes via natural language processing (NLP).”
  - “A web service for the ACC/AHA ASCVD risk calculator is prefilled with data from the EHR to determine the risk for a given patient. The user interface provides a risk score when MEA is accessed.”
  - “In order to collect and deliver the data in one service layer, a unified data platform (UDP) has been developed at Mayo Clinic. The UDP serves as a composite data source that is needed to execute the rules for the MEA initiative.”
  - “The identification of data needed at each node in the CPMs and the clinical logic portrayed therein was translated into programmable logic utilizing a collaborative effort between clinicians, informaticians, and technologists. Using C#.NET, this logic was encoded into a rules engine.”
- Intervention assessed by:
  - Time, number of clicks, and number of keystrokes collected as efficiency metrics.
  - Clinician ASVCD risk score calculations were compared to scores pre-calculated by MEA and compared as to whether care recommendation matched suggested hyperlipidemia care in the CPM.
  - A survey regarding ASVCD risk score calculator and MEA tool satisfaction was administered after the study was completed.

***Intervention Outcomes and Impacts***

- Statistically significant time savings: “The clinicians saved 3 minutes and 42 seconds in calculating the ASCVD score and a total of 3 minutes and 38 seconds in determining the recommendation.”
  - More details: “Without any assistance from MEA, the clinicians spent an average of 4 minutes and 21 seconds to calculate the ASCVD score and a total of 5 minutes and 8 seconds to additionally determine the care they would recommend for the patient. Qualitatively, some clinicians took a lot of time scrolling through previous notes to find parameters such as blood pressure rather than looking in parts of the EHR with discrete data, such as looking in the vitals section. With MEA, the clinicians spent 39 seconds to calculate the ASCVD score and a total of 1 minute and 31 seconds to calculate the ASCVD score and determine a recommendation for patient care.”
- “On average, there are 62 patients not on optimal cholesterol treatment seen in the Mayo Clinic primary care practice each day. If time savings is estimated only based on patients who need treatment, MEA would save the practice 3 hours and 45 minutes each day – approximately one-half day of clinician time.”
- Usage of MEA normalized time to complete tasks for all types of providers. Without MEA usage, there was a significant difference in time to complete tasks (calculation and recommendation) between NPs, PAs, and other clinician types. There was also a significant difference in time to complete tasks between residents and internal medicine physicians.
- Providers were more efficient with 94 fewer clicks and 23 fewer keystrokes with MEA, and improved accuracy from the baseline of 60.61% for both the risk score calculation and treatment recommendation.”
- Improved accuracy:
  - “The clinicians were found to have a 60.61% accuracy of ASCVD risk score calculation and 60.61% accuracy in selecting the guideline recommended statin treatment without MEA. The clinicians had a 100% accuracy of ASCVD risk score calculation and 100% accuracy in selecting the guideline recommended statin treatment with the use of MEA.”
  - There was no statistically significant difference between provider types.
  - “The most frequent errors that clinicians made in calculating the ASCVD risk score were not using the most recent blood pressure (41.7%) and incorrectly determining whether the patient was being treated for hypertension (33.3%). Clinicians also inputted the wrong gender, age, and smoking status.”
- Provider satisfaction survey results regarding MEA
  - “Of the clinicians surveyed, 51.5% indicated they calculate ASCVD risk most of the time or always, and 27.3% indicated that a high 30-year risk score may affect initiating treatment. Fewer than half of the clinicians used the risk calculator to encourage patients to quit smoking (45.4%) and lower blood pressure or cholesterol (46.8%).”
  - “Only 15% of the clinicians in this study indicated that they always will calculate a patient’s ASCVD risk score and this suggests that technology can have an integral role in ensuring consistent and accurate delivery of individualized recommendations.”

***Intervention Facilitators***

- Preexisting Mayo knowledge management system with extensive planning and IT infrastructure development.
- Institutional interest and financial investment in NLP capabilities which facilitated accuracy through auto population of most recent patient information for risk calculation.
- All the information being conveniently in one place facilitated the success of this intervention.
  - Convenient placement of the MEA directly into the EHR reduced the provider needing to move between separate application windows, potentially contributing to time saving.
    - “Mayo Clinic‘s EHR is in a general electric (GE) centricity environment. To improve the user interface Mayo Clinic has developed a viewer called Synthesis that used GE web services to retrieve data and present it to clinicians in a more intuitive format. MEA has been interfaced with the Synthesis thus enabling easy navigation for the clinicians to the risk score and any recommendations regarding cholesterol management.”
    - Further, having all relevant information pooled in one place through MEA additionally contributed: “Without any assistance from MEA, clinicians have to go through the EHR clicking on multiple tabs in order to find relevant data.”
- Automated data population helped improve accuracy: “With this knowledge delivery solution, data can be extracted automatically from the EHR and can populate the ACC risk score calculator to help clinicians deliver individualized treatment recommendations for patients with elevated cardiovascular risk.”

References

1. Geisinger 2017 Annual Report: Changing the face of healthcare. (2017). Retrieved June 18, 2020 from https://www.geisinger.org/-/media/OneGeisinger/pdfs/ghs/about-geisinger/news-and-media/annual-reports/87179-Geisinger-AR_2017_final5_spreads.pdf?la=en

2. Our service area. (n.d.) Geisinger. Retrieved June 18, 2020 from https://www.geisinger.org/health-plan/about/service-areas

3. For media: Fast facts. (n.d) Geisinger. Retrieved June 18, 2020 from https://www.geisinger.org/about-geisinger/news-and-media/for-media.

4. Community Health Needs Assessment. (2018). Geisinger. Retrieved June 18, 2020 from https://www.geisinger.org/about-geisinger/in-our-community/chna

5. Transforming Healthcare Through Continuous Innovation: 2014 System Report. (2014). Geisinger. Retrieved June 18, 2020 from https://www.geisinger.org/-/media/OneGeisinger/pdfs/ghs/about-geisinger/news-and-media/annual-reports/77219-1-2014SystemReport-Rev15-spreads.pdf?la=en

6. Paulus RA, Davis K, Steele GD. Continuous innovation in health care: implications of the Geisinger experience. Health Aff (Millwood). 2008 Sep-Oct;27(5):1235-45. doi: 10.1377/hlthaff.27.5.1235. PMID: 18780906

7. Transforming Healthcare Through Innovation: 2013 System Report. (2013). Geisinger. Retrieved June 18, 2020 from https://www.geisinger.org/-/media/OneGeisinger/pdfs/ghs/about-geisinger/news-and-media/annual-reports/system-report-111713.pdf?la=en

8. Berry, S.A., Doll, M.C., McKinley, K.E., Casale, A.S., & Bothe, A. (2009). ProvenCare: quality improvement model for designing highly reliable care in cardiac surgery. Qual Saf Health Care 2009;18:360–368. doi:10.1136/qshc.2007.025056 PMID: 19812098

9. Weber V, Bloom F, Pierdon S, Wood C. Employing the electronic health record to improve diabetes care: a multifaceted intervention in an integrated delivery system. J Gen Inter Med. 2008;23(4):379–8. doi: 10.1007/s11606-007-0439-2. PMID: 18373133.

10. Steele, G.D., Haynes, J.A., Davis, D.E., Tomcavage, J., Stewart, W.F., Graf, T.R., Paulus, R.A., Weikel, K.,& Shikles, J. (2010). How Geisinger's advanced medical home model argues the case for rapid-cycle innovation. Health Aff (Millwood). 2010 Nov;29(11):2047-53. doi: 10.1377/hlthaff.2010.0840. PMID: 21041747.

11. Maeng DD, Khan N, Tomcavage J, Graf TR, Davis DE, Steele GD. Reduced acute inpatient care was largest savings component of Geisinger Health System's patient-centered medical home. Health Aff (Millwood). 2015 Apr;34(4):636-44. doi:10.1377/hlthaff.2014.0855. PMID: 25847647.

12. Maeng DD, Davis DE, Tomcavage J, Graf TR, Procopio KM. Improving patient experience by transforming primary care: evidence from Geisinger's patient-centered medical homes. Popul Health Manag. 2013 Jun;16(3):157-63. doi: 10.1089/pop.2012.0048. Epub 2013 Feb 13. PubMed PMID: 23405878.

13. Maeng DD, Graf TR, Davis DE, Tomcavage J, Bloom FJ Jr. Can a patient-centered medical home lead to better patient outcomes? The quality implications of Geisinger's ProvenHealth Navigator. Am J Med Qual. 2012 May-Jun;27(3):210-6. doi: 10.1177/1062860611417421. Epub 2011 Aug 18. PubMed PMID: 21852292.

14. Gilfillan RJ, Tomcavage J, Rosenthal MB, Davis DE, Graham J, Roy JA, Pierdon SB, Bloom FJ Jr, Graf TR, Goldman R, Weikel KM, Hamory BH, Paulus RA, Steele GD Jr. Value and the medical home: effects of transformed primary care. Am J Manag Care. 2010 Aug;16(8):607-14. PubMed PMID: 20712394.

15. Tomcavage J, Littlewood D, Salek D, Sciandra J. Advancing the role of nursing in the medical home model. Nurs Adm Q. 2012 Jul-Sep;36(3):194-202. doi:10.1097/NAQ.0b013e3182588b6a. PubMed PMID: 22677959.

16. Williams, M.S., Buchanan, A.H., Davis, F.D., Faucett, W.A., Hallquist, M.L.G., Leader, J.B., Martin, C.L., McCormick, C.Z., Meyer, M.N., Murray, M.F., Rahm, A.K., Schwartz, M.L.B., Sturm, A.C., Wagner, J.K., Williams, J.L., Willard, H.F., & Ledbetter, D.H. (2018) Patient-Centered Precision Health In A Learning Health Care System: Geisinger's Genomic Medicine Experience. Health Aff (Millwood). 2018 May;37(5):757-764. doi: 10.1377/hlthaff.2017.1557. PMID: 29733722.

17. Bailey-Davis L, Kling SMR, Cochran WJ, Hassink S, Hess L, Franceschelli Hosterman, J, Lutcher S, Marini M, Mowery J, Paul IM, & Savage JS. Integrating and coordinating care between the Women, Infants, and Children Program and pediatricians to improve patient-centered preventive care for healthy growth. Transl Behav Med. 2018 Nov 21;8(6):944-952. doi: 10.1093/tbm/ibx046. PubMed PMID: 29370433.

18. Vartoella, L. Steal this Idea: Geisinger CEO David Feinberg explains how giving back money is his system's most valuable expense. Becker’s Hospital Review. April 10, 2018. Retrieved June 18, 2020 from https://www.beckershospitalreview.com/hospital-management-administration/steal-this-idea-geisinger-ceo-david-feinberg-explains-how-giving-away-money-is-his-system-s-most-valuable-expense.html

19. Fresh Food Farmacy. (n.d.). Geisinger. Retrieved June 18, 2020 from https://www.geisinger.org/freshfoodfarmacy

20. Health and Wellness Impacts. (n.d.) Geisinger. Retrieved June 18, 2020 from https://www.geisinger.org/innovation-steele-institute/our-pillars/health

21. During your child’s stay: A guide for patients and the families by patients and their families. (n.d.) Geisinger Janet Weis Children’s Hospital. Retrieved June 18, 2020 from https://www.geisinger.org/-/media/OneGeisinger/pdfs/ghs/patient-care/find-a-location/jwch/janet-weis-childrens-hospital-handbook.pdf.

22. Convenient Care Wait Times. (n.d.) Geisinger. Retrieved June 18, 2020 from https://www.geisinger.org/patient-care/find-a-location/urgent-care-wait-times?utm_source=Locations%20Page&utm_medium=Web&utm_campaign=Urgent%20Care%20CTA

23. Geisinger Community Medical Center: A guide for patients and their families. (n.d.) Geisinger. Retrieved June 18, 2020 from https://www.geisinger.org/-/media/OneGeisinger/pdfs/ghs/patient-care/find-a-location/gcmc/geisinger-community-medical-center-handbook.pdf?la=en

24. Graham, J., Tomcavage, J., Salek, D., Sciandra, J., Davis, D.E., & Stewart, W.F. (2012). Postdischarge monitoring using interactive voice response system reduces 30-day readmission rates in a case-managed Medicare population. Med Care. 2012 Jan;50(1):50-7. doi: 10.1097/MLR.0b013e318229433e. PMID: 21822152

25. Maeng, D.D., Snyder, S.R., Davis, T.W., Tomcavage, J.F. (2017). Impact of a Complex Care Management Model on Cost and Utilization Among Adolescents and Young Adults with Special Care and Health Needs. Popul Health Manag. 2017 Dec;20(6):435-441. doi: 10.1089/pop.2016.0167. Epub 2017 Mar 24. PMID: 28338416.

26. About: Fast facts. (n.d). Kaiser Permanente. Retrieved June 18, 2020 from https://about.kaiserpermanente.org/who-we-are/fast-facts

27. 2018 Annual Report. (2018). Kaiser Permanente. Retrieved June 18, 2020 from https://healthy.kaiserpermanente.org/static/health/annual_reports/kp_annualreport_2018/

28. Maeda JLK, Lee KM, & Horberg M. (2014). Comparative Health Systems Research among Kaiser Permanente and Other Integrated Delivery Systems: A Systematic Literature Review. Perm J. 2014 Summer; 18(3): 66–77. Prepublished online 2014 Jun 9. doi: 10.7812/TPP/13-159. PMID: 24937150

29. Pines J, Selevan J, McStay F, George M, & McClellan M; Center for Health Policy at Brookings. (2015). Kaiser Permanente – California: A Model for Integrated Care for the Ill and Injured. Published May 4, 2015. Retrieved June 18, 2020 from https://www.brookings.edu/wp-content/uploads/2016/07/KaiserFormatted_150504RH-with-image.pdf.

30. Institute of Medicine. (2011). The Future of Nursing: Leading Change, Advancing Health. Washington, DC: The National Academies Press. https://doi.org/10.17226/12956.

31. Liu VX, Morehouse JW, Baker JM, Greene JD, Kipnis P, & Escobar GJ. (2017). Data That Drive: Closing the Loop in the Learning Hospital System. J Hosp Med. 2016 Nov; 11(Suppl 1): S11–S17. doi: 10.1002/jhm.2651. PMID: 27805797.

32. Liu VX, Rosas E, Hwang JC, Cain E, Foss-Durant A, Clopp M, Huang M, Mustille A, Reyes VM, Paulson SS, Caughey M, & Parodi S. The Kaiser Permanente Northern California Enhanced Recovery After Surgery Program: Design, Development, and Implementation. Perm J. 2017;21:17-003. doi: 10.7812/TPP/17-003. PubMed PMID: 28746028; PubMed Central PMCID: PMC5528846.

33. Qiu C, Rinehart J, Nguyen VT, Cannesson M, Morkos A, LaPlace D, Trivedi NS, Mercado PD, & Kain ZN. An Ambulatory Surgery Perioperative Surgical Home in Kaiser Permanente Settings: Practice and Outcomes. Anesth Analg. 2017 Mar;124(3):768-774. doi: 10.1213/ANE.0000000000001717. PubMed PMID: 28027086.

34. Liu V, Herbert D, Foss-Durant A, Marelich GP, Patel A, Whippy A, Turk BJ, Ragins AI, Kipnis P, & Escobar GJ. Evaluation Following Staggered Implementation of the "Rethinking Critical Care" ICU Care Bundle in a Multicenter Community Setting. Crit Care Med. 2016 Mar;44(3):460-7. doi: 10.1097/CCM.0000000000001462. PubMed PMID: 26540402; PubMed Central PMCID: PMC4764399.

35. Tuso P, Watson HL, Garofalo-Wright L, Lindsay G, Jackson A, Taitano M, Koyama S, & Kanter M. Complex case conferences associated with reduced hospital admissions for high-risk patients with multiple comorbidities. Perm J. 2014 Winter;18(1):38-42. doi: 10.7812/TPP/13-062. PubMed PMID: 24626071; PubMed Central PMCID: PMC3951029.

36. Liu VX, Rosas E, Hwang J, et al. Enhanced recovery after surgery program implementation in 2 surgical populations in an integrated health care delivery system. JAMA Surg. 2017 May 10:e171032. DOI: https://doi.org/10.1001/jamasurg.2017.1032.

37. Reed ME, Huang J, Brand R, Ballard D, Yamin C, Hsu J, & Grant R. Communicating Through a Patient Portal to Engage Family Care Partners. JAMA Intern Med. 2018 Jan 1;178(1):142-144. doi: 10.1001/jamainternmed.2017.6325. PubMed PMID: 29159402; PubMed Central PMCID: PMC5833508.

38. Tuzzio L, Ludman EJ, Chang E, Palazzo L, Abbott T, Wagner EH, & Reid RJ. Design and Implementation of a Physician Coaching Pilot to Promote Value-Based Referrals to Specialty Care. Perm J. 2017;21:16-066. doi: 10.7812/TPP/16-066. PubMed PMID: 28368789; PubMed Central PMCID: PMC5378485.

39. Stein T, Frankel RM, & Krupat E. Enhancing clinician communication skills in a large healthcare organization: a longitudinal case study. Patient Educ Couns. 2005 Jul;58(1):4-12. PubMed PMID: 15950831.

40. Berry ABL, Lim C, Hartzler AL, Hirsch T, Ludman E, Wagner EH, & Ralston JD. Eliciting Values of Patients with Multiple Chronic Conditions: Evaluation of a Patient-centered Framework. AMIA Annu Symp Proc. 2018 Apr 16;2017:430-439.eCollection 2017. PubMed PMID: 29854107; PubMed Central PMCID: PMC5977727.

41. Glass JE, Bobb JF, Lee AK, Richards JE, Lapham GT, Ludman E, Achtmeyer C, Caldeiro RM, Parrish R, Williams EC, Lozano P, & Bradley KA. Study protocol: a cluster-randomized trial implementing Sustained Patient-centered Alcohol-related Care (SPARC trial). Implement Sci. 2018 Aug 6;13(1):108. doi: 10.1186/s13012-018-0795-9. PubMed PMID: 30081930; PubMed Central PMCID: PMC6080376.

42. Green BB, Anderson ML, Wang CY, Vernon, SW, Chubak J, Meenan RT, & Fuller S. Results of nurse navigator follow-up after positive colorectal cancer screening test: a randomized trial. J Am Board Fam Med. 2014 Nov-Dec;27(6):789-95. doi: 10.3122/jabfm.2014.06.140125. PubMed PMID: 25381076; PubMed Central PMCID: PMC4278960.

43. Sperl-Hillen JM, Rossom RC, Kharbanda EO, Gold R, Geissal ED, Elliott TE, Desai JR, Rindal DB, Saman DM, Waring SC, Margolis KL, & O'Connor, PJ. Priorities Wizard: Multisite Web-Based Primary Care Clinical Decision Support Improved Chronic Care Outcomes with High Use Rates and High Clinician Satisfaction Rates. EGEMS (Wash DC). 2019 Apr 3;7(1):9. doi: 10.5334/egems.284. Review. PubMed PMID: 30972358; PubMed Central PMCID: PMC6450247.

44. Lieu TA, Herrinton LJ, Buzkov DE, Liu L, Lyons D, Neugebauer R, Needham T, Ng D, Prausnitz S, Stewart K, Van Den Eeden, SK, & Baer DM. Developing a Prognostic Information System for Personalized Care in Real Time. EGEMS (Wash DC). 2019 Mar 25;7(1):2. doi: 10.5334/egems.266. PubMed PMID: 30937324; PubMed Central PMCID: PMC6437692.

45. O'Leary K, Tanghe D, Pratt W, & Ralston J. Collaborative Health Reminders and Notifications: Insights from Prototypes. AMIA Annu Symp Proc. 2018 Dec 5;2018:837-846. eCollection 2018. PubMed PMID: 30815126; PubMed Central PMCID: PMC6371389.

46. Graetz I, Reed M, Shortell SM, Rundall TG, Bellows J, & Hsu J. The next step towards making use meaningful: electronic information exchange and care coordination across clinicians and delivery sites. Med Care. 2014 Dec;52(12):1037-41. doi: 10.1097/MLR.0000000000000245. PubMed PMID: 25304020; PubMed Central PMCID: PMC5131789.

47. MacPhail LH, Neuwirth EB, & Bellows J. Coordination of diabetes care in four delivery models using an electronic health record. Med Care. 2009 Sep;47(9):993-9. doi: 10.1097/MLR.0b013e31819e1ffe. PubMed PMID: 19648836.

48. Graetz I, Reed M, Rundall T, Bellows J, Brand R, & Hsu J. Care coordination and electronic health records: connecting clinicians. AMIA Annu Symp Proc. 2009 Nov 14;2009:208-12. PubMed PMID: 20351851; PubMed Central PMCID: PMC2815429.

49. Bender BG, Cvietusa PJ, Goodrich GK, et al. Pragmatic trial of health care technologies to improve adherence to pediatric asthma treatment: a randomized clinical trial. JAMA Pediatr. 2015;169(4):317-323. doi:10.1001/jamapediatrics.2014.3280

50. Dimidjian S, Beck A, Felder JN, Boggs JM, Gallop R, Segal ZV. Web-based Mindfulness-based Cognitive Therapy for reducing residual depressive symptoms: An open trial and quasi-experimental comparison to propensity score matched controls. Behav Res Ther. 2014;63:83-89.

51. Pressman AR, Kinoshita L, Kirk S, Barbosa GM, Chou C, & Minkoff J. A novel telemonitoring device for improving diabetes control: protocol and results from a randomized clinical trial. Telemed J E Health. 2014;20(2):109-114. doi:10.1089/tmj.2013.0157

52. Rosner BI, Gottlieb M, & Anderson WN. Effectiveness of an Automated Digital Remote Guidance and Telemonitoring Platform on Costs, Readmissions, and Complications After Hip and Knee Arthroplasties. J Arthroplasty. 2018;33(4):988-996.e4. doi:10.1016/j.arth.2017.11.036

53. Sauser-Zachrison K, Shen E, Sangha, N, et al. Safe and Effective Implementation of Telestroke in a US Community Hospital Setting. Perm J. 2016;20(4):15-217. doi:10.7812/TPP/15-217

54. Nguyen-Huynh MN, Klingman JG, Avins AL, et al. Novel Telestroke Program Improves Thrombolysis for Acute Stroke Across 21 Hospitals of an Integrated Healthcare System. Stroke. 2018;49(1):133-139. doi:10.1161/STROKEAHA.117.018413

55. Kaiser Permanente. July 3 2019. Expanding mental health care services in California. Retrieved June 18 2020 from https://about.kaiserpermanente.org/our-story/news/announcements/expanding-mental-health-care-services-in-california31

56. Rice, Y.B., Barnes, C.A., Rastogi, R., Hillstrom. T.J., & Steinkeler, C.N. (2016). Tackling 30-Day, All-Cause Readmissions with a Patient-Centered Transitional Care Bundle. Popul Health Manag. 2016 Feb;19(1):56-62. doi: 10.1089/pop.2014.0163. PubMed PMID: 25919315. Epub 2015 Apr 28.

57. Lin, M., Heisler, S., Fahey, L.., McGinnis, J., & Whiffen, T.L. (2015). Nurse Knowledge Exchange Plus: Human-Centered Implementation for Spread and Sustainability. Jt Comm J Qual Patient Saf. 2015 Jul;41(7):303-12. PubMed PMID: 26108123

58. McGaw, J., Conner, D.A., Delate, T.M., Chester, E.A., & Barnes, C.A. (2007). A multidisciplinary approach to transition care: a patient safety innovation study. Perm J. 2007 Fall;11(4):4-9. PMID: 21412475.

59. Stubbings, T., Miller, C., Humphries, T.L., Nelson, K.M., & Helling, D.K. (2005). Telepharmacy in a health maintenance organization. Am J Health Syst Pharm. 2005 Feb 15;62(4):406-10. doi: 10.1093/ajhp/62.4.0406 PMID: 15745894

60. Community Health Needs Assessment 2019 (Cleveland Clinic Main Campus). (2019) Cleveland Clinic Retrieved June 18, 2020 from https://my.clevelandclinic.org/-/scassets/files/org/about/community-reports/chna/2019/2019-cleveland-clinic-main-campus-chna.ashx?la=en

61. State of the Clinic 2019. (2019). Cleveland Clinic. Retrieved June 18, 2020 from https://my.clevelandclinic.org/-/scassets/files/org/about/who-we-are/state-of-the-clinic.ashx?la=en

62. Governance & Leadership. (n.d.) Cleveland Clinic. Retrieved June 18, 2020 from https://my.clevelandclinic.org/about/overview/leadership

63. Cosgrove, D.M. (2011, March). A Healthcare Model for the 21st Century: Patient-Centered, Integrated Delivery Systems. Group Practice Journal. Retrieved June 18, 2020 from https://my.clevelandclinic.org/-/scassets/files/org/about/model-healthcare/amga-mar-2011.ashx?la=en

64. Cleveland Clinic Improvement Model (CCIM). (2018). Cleveland Clinic. Retrieved June 18, 2020 from https://my.clevelandclinic.org/-/scassets/files/org/clinical-transformation/cc-improvement-model.ashx?la=en

65. Yerian, Lisa. (2018, May 10) How to create a culture of continuous improvement: Proven success through 4 integral systems. Cleveland Clinic. Retrieved February 12, 2020 from https://consultqd.clevelandclinic.org/how-to-create-a-culture-of-continuous-improvement/

66. Cleveland Clinic. (2018). Cleveland Clinic Improvement Model (CCIM): Harnessing the power of every one to achieve our goals. Retrieved February 12, 2020 from https://my.clevelandclinic.org/-/scassets/files/org/clinical-transformation/cc-improvement-model.ashx?la=en

67. Our Model of Healthcare. (n.d.) Cleveland Clinic. Retrieved June 18, 2020 from https://my.clevelandclinic.org/about/overview/our-model-healthcare

68. Merlino, J.I., & Raman, A. (2003 May). Healthcare’s Service Fanatics. Harvard Business Review, 108-116. Retrieved February 11, 2020 from https://my.clevelandclinic.org/ccf/media/Files/Patient-Experience/cehc-overview.pdf

69. Lee WW, Alkureishi ML, Isaacson JH, Mayer M, Frankel RM, London DA, Wroblewski KE, Arora VM. Impact of a brief faculty training to improve patient-centered communication while using electronic health records. Patient Educ Couns. 2018 Dec;101(12):2156-2161. doi: 10.1016/j.pec.2018.06.020. Epub 2018 Jul 3. PubMed PMID: 30007764.

70. Boissy, A., Windover, A.K., Bokar, D., Karafa, M., Neuendorf, K., Frankel, R.M., Merlino, J., & Rothberg, M.B. (2016) Communication Skills Training for Physicians Improves Patient Satisfaction. J Gen Intern Med. 2016 Jul;31(7):755-61. doi: 10.1007/s11606-016-3597-2. Epub 2016 Feb 26. PMID: 26921153

71. Flicker LS, Rose SL, Eves MM, Flamm AL, Sanghani R, Smith ML. Developing and testing a checklist to enhance quality in clinical ethics consultation. J Clin Ethics. 2014 Winter;25(4):281-90. PubMed PMID: 25517564; PubMed Central PMCID: PMC4552192.

72. Schneeberger D, Golubíc M, Moore HCF, et al. Lifestyle Medicine-Focused Shared Medical Appointments to Improve Risk Factors for Chronic Diseases and Quality of Life in Breast Cancer Survivors. J Altern Complement Med. 2019;25(1):40-47. doi:10.1089/acm.2018.0154

73. Kostick KM, Bruce CR, Minard CG, Volk RJ, Civitello A, Krim SR, Horstmanshof D, Thohan V, Loebe M, Hanna M, Bruckner BA, Blumenthal Barby JS, Estep JD. A Multisite Randomized Controlled Trial of a Patient-Centered Ventricular Assist Device Decision Aid (VADDA Trial). J Card Fail. 2018 Oct;24(10):661-671. doi: 10.1016/j.cardfail.2018.08.008. Epub 2018 Sep 7. PubMed PMID: 30195826.

74. Kozak VN, Khorana AA, Amarnath S, Glass KE, Kalady MF. Multidisciplinary Clinics for Colorectal Cancer Care Reduces Treatment Time. Clin Colorectal Cancer. 2017 Dec;16(4):366-371. doi: 10.1016/j.clcc.2017.03.020. Epub 2017 Apr 19. PubMed PMID: 28527628.

75. Russo AN, Sathiyamoorthy G, Lau C, Saygin D, Han X, Wang XF, Rice R, Aboussouan LS, Stoller JK, Hatipoğlu U. Impact of a Post-Discharge Integrated Disease Management Program on COPD Hospital Readmissions. Respir Care. 2017 Nov;62(11):1396-1402. doi: 10.4187/respcare.05547. Epub 2017 Aug 1. PubMed PMID: 28765496.

76. Appachi S, Banas A, Feinberg L, Henry D, Kenny D, Kraynack N, Rosneck A, Carl J, Krakovitz P. Association of Enrollment in an Aerodigestive Clinic With Reduced Hospital Stay for Children With Special Health Care Needs. JAMA Otolaryngol Head Neck Surg. 2017 Nov 1;143(11):1117-1121. doi: 10.1001/jamaoto.2017.1743. PubMed PMID: 28983551; PubMed Central PMCID: PMC5710347.

77. London DA, Vilensky S, O'Rourke C, Schill M, Woicehovich L, Froimson MI. Discharge Disposition After Joint Replacement and the Potential for Cost Savings: Effect of Hospital Policies and Surgeons. J Arthroplasty. 2016;31(4):743-748. doi:10.1016/j.arth.2015.10.014

78. Devarakonda MV, Mehta N, Tsou CH, Liang JJ, Nowacki AS, Jelovsek JE. Automated problem list generation and physicians perspective from a pilot study. Int J Med Inform. 2017 Sep;105:121-129. doi: 10.1016/j.ijmedinf.2017.05.015. Epub 2017 Jun 4. PubMed PMID: 28750905.

79. Ethics Consultation Service. (n.d) Cleveland Clinic, Center for Bioethics. Retrieved June 18, 2020 from https://my.clevelandclinic.org/departments/patient-experience/depts/bioethics/bioethics/clinical-ethics#ethics-consultation-service-tab

80. Minority Men’s Health. (n.d.) Cleveland Clinic. Retrieved June 18, 2020 from https://my.clevelandclinic.org/departments/urology-kidney/depts/minority-mens-health-center

81. Center for Connected Care. (n.d.) Cleveland Clinic. Retrieved June 18, 2020 from https://my.clevelandclinic.org/departments/connected-care

82. ConsultQD. (2018, September 28) Care coordinators help high-risk patients: Focus on overall health as part of primary care practices. Cleveland Clinic. Retrieved February 12, 2020 from https://consultqd.clevelandclinic.org/care-coordinators-help-high-risk-patients/

83. Cleveland Clinic. (2020, February 11). Institutes and Departments. https://my.clevelandclinic.org/departments

84. Cleveland Clinic. (2020, February 11). Experience Partners. https://my.clevelandclinic.org/departments/patient-experience/depts/experience-partners

85. Office of Patient Experience. (n.d.) Cleveland Clinic. Retrieved June 18, 2020 from https://my.clevelandclinic.org/departments/patient-experience/depts/office-patient-experience83

86. Quality & Patient Safety Institute. (n.d.) Cleveland Clinic. Retrieved June 18, 2020 from https://my.clevelandclinic.org/departments/patient-experience/depts/quality-patient-safety

87. Continuous Improvement. (n.d.) Cleveland Clinic. Retrieved June 18, 2020 from https://my.clevelandclinic.org/departments/patient-experience/depts/continuous-improvement#overview-tab

88. Kim, L.D., Kou, L., Hu, B., Gorodeski, E.Z., & Rothberg, M.B. (2017) Impact of a Connected Care model on 30-day readmission rates from skilled nursing facilities. J Hosp Med. 2017 Apr;12(4):238-244. doi: 10.12788/jhm.2710. PMID: 28411287

89. Windover, A.K., Boissy, A., Rice, T.W., Gilligan, T., Velez, V.J., & Merlino, J. (2014) The REDE Model of Healthcare Communication: Optimizing Relationship as a Therapeutic Agent. J Patient Exp. 2014 May;1(1):8-13. doi: 10.1177/237437431400100103. Epub 2014 May 1. PMID: 28725795

90 . Taqui, A., Cerejo, R., Itrat, A., Briggs, F.B., Reimer, A.P., Winners, S., Organek, N., Buletko, A.B., Sheikhi, L., Cho, S.M., Buttrick, M., Donohue, M.M., Khawaja, Z., Wisco, D., Frontera, J.A., Russman, A.N., Hustey, F.M., Kralovic, D.M., Rasmussen, P., Uchino, K., […] & Cleveland Pre-Hospital Acute Stroke Treatment (PHAST) Group. (Reduction in time to treatment in prehospital telemedicine evaluation and thrombolysis.) Neurology. 2017 Apr 4;88(14):1305-1312. doi: 10.1212/WNL.0000000000003786. Epub 2017 Mar 8. PMID: 28275084

91. Mayo Clinic Mission and Values. (n.d.) Mayo Clinic. Retrieved June 18, 2020 from https://www.mayoclinic.org/about-mayo-clinic/mission-values

92. McCarthy D, Mueller K, & Wren J.; The Commonwealth Fund. (2009, Aug.) Mayo Clinic: Multidisciplinary Teamwork, Physician-Led Governance, and Patient-Centered Culture Drive World-Class Health Care. Retrieved June 18, 2020 from https://www.commonwealthfund.org/sites/default/files/documents/___media_files_publications_case_study_2009_aug_1306_mccarthy_mayo_case_study.pdf

93. Facts and highlights. (2011). Mayo Clinic. Retrieved June 18, 2020 from https://www.mayoclinic.org/documents/mc2045-pdf/doc-20078949

94. An Inside Look at Mayo Clinic. (2019). Retrieved June 18, 2020 from https://mcforms.mayo.edu/mc7300-mc7399/mc7360.pdf and Fast facts

95. 2017 Report to Our Community. (2017). Mayo Clinic Health System. Retrieved June 18, 2020 from https://www.mayoclinichealthsystem.org/-/media/local-files/la-crosse/la-crosse-live-pdf-files/report-to-community-2017.pdf?la=en&rev=7f42ea5c795c4ee3af77be36b290f6de&hash=FF69E18843229B2092F3586F81B75067

96. Anastasijevic D. Mayo Clinic and Medica announce plans to create insurance products. Mayo Clinic New Network. Sept 13, 2018. Retrieved June 18, 2020 from https://newsnetwork.mayoclinic.org/discussion/mayo-clinic-and-medica-announce-plans-to-create-insurance-products/116

97. Viggiano, T.R., Pawlina, W., Lindor, K.D., Olsen, K.D., & Cortese, D.A. (2007). Putting the needs of the patient first: Mayo Clinic's core value, institutional culture, and professionalism covenant. Acad Med. 2007 Nov;82(11):1089-93. doi: 10.1097/ACM.0b013e3181575dcd PMID: 17971697.

98. Shih A, Davis K, Schoenbaum SC, Gauthier A, Nuzum R, & McCarthy D.; Commonwealth Fund. (2008). Organizing the U.S. Healthcare Delivery System for High Performance. Retrieved June 18. 2020 from https://www.commonwealthfund.org/sites/default/files/documents/___media_files_publications_fund_report_2008_aug_organizing_the_u_s__health_care_delivery_system_for_high_performance_shih_organizingushltcaredeliverysys_1155_pdf.pdf

99. Shellum, J,L., Nishimura, R.A., Milliner, D.S., Harper, C,M., & Noseworthy, J.H. (2017). Knowledge management in the era of digital medicine: A programmatic approach to optimize patient care in an academic medical center. Learn Health Syst. 2017 Feb 13;1(2):e10022. doi: 10.1002/lrh2.10022. eCollection 2017 Apr. PMID: 31245559.

100. Kaggal, V.C., Elayavilli, R.K., Mehrabi, S., Pankratz, J.J., Sohn, S., Wang, Y., Li, D., Rastegar, M.M., Murphy, S.P., Ross, J.L., Chaudhry, R., Buntrock, J.D., Liu, H. (2016). Toward a Learning Health-care System – Knowledge Delivery at the Point of Care Empowered by Big Data and NLP. Biomed Inform Insights. 2016 Jun 23;8(Suppl 1):13-22. doi: 10.4137/BII.S37977. eCollection 2016. PMID: 27385912.

101. Kreofsky, B.L.H., Blegen, N., Lokken, T., Kapraun, S.M., Bushman, M.S., & Demaerschalk, B.M. (2018). Sustainable Telemedicine: Designing and Building Infrastructure to Support a Comprehensive Telemedicine Practice. Telemed J E Health. 2018 Dec;24(12):1021-1025. doi: 10.1089/tmj.2017.0291. Epub 2018 Apr 16. PMID: 29658828.

102. Wald, J.T., Lowery-Schrandt, S., Hayes, D.L., & Kotsenas, A.L. (2018). Mayo Clinic Care Network: A Collaborative Health Care Model. J Am Coll Radiol. 2018 Jan;15(1 Pt B):167-172. doi: 10.1016/j.jacr.2017.09.031. Epub 2017 Nov 6. PMID: 29122505.

103. Feely, M.A., Swetz, K.M., Zavaleta, K., Thorsteinsdottir, B., Albright, R.C., & Williams, A.W. (2016). Reengineering Dialysis: The Role of Palliative Medicine. J Palliat Med. 2016 Jun;19(6):652-5. doi: 10.1089/jpm.2015.0181. Epub 2016 Mar 18. PMID: 26991732.

104. Garrison, G.M., Angstman, K.B., O'Connor, S.S., Williams, M.D., & Lineberry, T.W. (2016). Time to Remission for Depression with Collaborative Care Management (CCM) in Primary Care. J Am Board Fam Med. 2016 Jan-Feb;29(1):10-7. doi: 10.3122/jabfm.2016.01.150128. PubMed PMID: 26769872.

105. Young, N.P., Elrashidi, M.Y., Crane, S.J., Ebbert, J.O. (2016). Pilot of integrated, collocated neurology in a primary care medical home. J Eval Clin Pract. 2017 Jun;23(3):548-553. doi: 10.1111/jep.12667. Epub 2016 Dec 12. PMID:27943579

106. North, F., Uthke, L.D., & Tulledge-Scheitel, S.M. (2015). Internal e-consultations in an integrated multispecialty practice: a retrospective review of use, content, and outcomes. J Telemed Telecare. 2015 Apr;21(3):151-9. doi: 10.1177/1357633X15572204. Epub 2015 Feb 22. PMID: 25712108.

107. North, F., Uthke, L.D., & Tulledge-Scheitel, S.M. (2014). Integration of e-consultations into the outpatient care process at a tertiary medical centre. J Telemed Telecare. 2014 Jun;20(4):221-229. Epub 2014 May 6. PMID: 24803274.

108. Angstman, K.B., Rohrer, J.E., Adamson, S.C., & Chaudhry, R. (2009). Impact of e-consults on return visits of primary care patients. Health Care Manag (Frederick). 2009 Jul-Sep;28(3):253-7. doi: 10.1097/HCM.0b013e3181b3efa3. PMID: 19668067.

109. Penza, K.S., Murray, M.A., Pecina, J.L., Myers, J.F., & Furst, J.W. (2018). Electronic Visits for Minor Acute Illnesses: Analysis of Patient Demographics, Prescription Rates, and Follow-Up Care Within an Asynchronous Text-Based Online Visit. Telemed J E Health. 2018 Mar;24(3):210-215. doi: 10.1089/tmj.2017.0091. Epub 2017 Jul 20.175

110. Scheitel, M.R., Kessler, M.E., Shellum, J.L., Peters, S.G., Milliner, D.S., Liu, H., Komandur Elayavilli, R., Poterack, K.A., Miksch, T.A., Boysen, J., Hankey, R.A., & Chaudhry, R. (2017) Effect of a Novel Clinical Decision Support Tool on the Efficiency and Accuracy of Treatment Recommendations for Cholesterol Management. Appl Clin Inform. 2017 Feb 8;8(1):124-136. doi: 10.4338/ACI-2016-07-RA-0114. PMID: 28174820

111. Cook DA, Enders F, Linderbaum JA, Zwart D, Lloyd FJ. Speed and accuracy of a point of care web-based knowledge resource for clinicians: a controlled crossover trial. Interact J Med Res. 2014;3(1):e7. Published 2014 Feb 21. doi:10.2196/ijmr.2811

112. Cook DJ, Pulido JN, Thompson JE, et al. Standardized practice design with electronic support mechanisms for surgical process improvement: reducing mechanical ventilation time. Ann Surg. 2014;260(6):1011-1015. doi:10.1097/SLA.0000000000000726

113. de Mooij MJM, Hodny RL, O'Neil DA, Gardner MR, Beaver M, Brown AT, Barry BA, Ross LM, Jasik AJ, Nesbitt KM, Sobolewski SM, Skinner SM, Chaudhry R, Brost BC, Gostout BS, Harms RW. OB Nest: Reimagining Low-Risk Prenatal Care. Mayo Clin Proc. 2018 Apr;93(4):458-466. doi: 10.1016/j.mayocp.2018.01.022. Epub 2018 Mar 12. Review. PubMed PMID: 29545005.

114. Siegel J, Edwards E, Mooney L, Smith C, Peel JB, Dole A, Maler P, Freeman WD. A feasibility pilot using a mobile personal health assistant (PHA) app to assist stroke patient and caregiver communication after hospital discharge. Mhealth. 2016 Aug 9;2:31. doi: 10.21037/mhealth.2016.08.02. eCollection 2016. PubMed PMID: 28293604; PubMed Central PMCID: PMC5344132.

115. Kazemian P, Sir MY, Van Oyen MP, Lovely JK, Larson DW, Pasupathy KS. Coordinating clinic and surgery appointments to meet access service levels for elective surgery. J Biomed Inform. 2017 Feb;66:105-115. doi: 10.1016/j.jbi.2016.11.007. Epub 2016 Dec 16. PubMed PMID: 27993748.

116. Kaleem T, Miller D, Waddle MR, Yanez M, Gianforti B, Buskirk S. Implementation of patient pagers in radiation oncology waiting rooms for patient privacy and satisfaction. BMC Res Notes. 2018 Jan 22;11(1):59. doi: 10.1186/s13104-018-3164-5. PubMed PMID: 29357904; PubMed Central PMCID: PMC5778759.

117. Demaerschalk BM, Boyd EL, Barrett KM, et al. Comparison of Stroke Outcomes of Hub and Spoke Hospital Treated Patients in Mayo Clinic Telestroke Program. J Stroke Cerebrovasc Dis. 2018;27(11):2940-2942. doi:10.1016/j.jstrokecerebrovasdis.2018.06.024

118. Beck, J.A., Jensen, J.A., Putzier, R.F., Stubert, L.A., Stuart, K.D., Mohammed, H., Kreofsky, B.L., Boles, K.W., Colby, C.E., & Fang, J.L. (2017). Developing a Newborn Resuscitation Telemedicine Program: A Comparison of Two Technologies. Telemed J E Health. 2018 Jul;24(7):481-488. doi: 10.1089/tmj.2017.0121. Epub 2017 Dec 12. PMID: 29232175.

119. O'Carroll, C.B., Hentz, J.G., Aguilar, M.I., & Demaerschalk, B.M. (2015). Robotic Telepresence Versus Standardly Supervised Stroke Alert Team Assessments. Telemed J E Health. 2015 Mar;21(3):151-6. doi: 10.1089/tmj.2014.0064. Epub 2014 Dec 9. PMID: 25490742

120. Demaerschalk, B.M., Vegunta, S., Vargas, B.B., Wu, Q., Channer, D.D., & Hentz, J.G. (2015). Reliability of Real-Time Video Smartphone for Assessing National Institutes of Health Stroke Scale Scores in Acute Stroke Patients. Stroke. 2012 Dec;43(12):3271-7. doi: 10.1161/STROKEAHA.112.669150. Epub 2012 Nov 15. PMID: 23160878183

121. Coylewright M, Dick S, Zmolek B, Askelin J, Hawkins E, Branda M, Inselman JW, Zeballos-Palacios C, Shah ND, Hess EP, LeBlanc A, Montori VM, Ting HH. PCI Choice Decision Aid for Stable Coronary Artery Disease: A Randomized Trial. Circ Cardiovasc Qual Outcomes. 2016 Nov;9(6):767-776. doi: 10.1161/CIRCOUTCOMES.116.002641. Epub 2016 Nov 1. PubMed PMID: 27803090.64

122. LeBlanc A, Herrin J, Williams MD, Inselman JW, Branda ME, Shah ND, Heim EM, Dick SR, Linzer M, Boehm DH, Dall-Winther KM, Matthews MR, Yost KJ, Shepel KK, Montori VM. Shared Decision Making for Antidepressants in Primary Care: A Cluster Randomized Trial. JAMA Intern Med. 2015 Nov;175(11):1761-70. doi: 10.1001/jamainternmed.2015.5214. PubMed PMID: 26414670; PubMed Central PMCID: PMC4754973.

123. Hargraves I, LeBlanc A, Shah ND, Montori VM. Shared Decision Making: The Need For Patient-Clinician Conversation, Not Just Information. Health Aff (Millwood). 2016 Apr;35(4):627-9. doi: 10.1377/hlthaff.2015.1354. Review. PubMed PMID: 27044962.

124. Ballard AY, Kessler M, Scheitel M, Montori VM, Chaudhry R. Exploring differences in the use of the statin choice decision aid and diabetes medication choice decision aid in primary care. BMC Med Inform Decis Mak. 2017 Aug 10;17(1):118. doi: 10.1186/s12911-017-0514-5. PubMed PMID: 28797295; PubMed Central PMCID: PMC5553736.

125. Boehmer KR, Hargraves IG, Allen SV, Matthews MR, Maher C, Montori VM. Meaningful conversations in living with and treating chronic conditions: development of the ICAN discussion aid. BMC Health Serv Res. 2016;16(1):514. Published 2016 Sep 23. doi:10.1186/s12913-016-1742-6

126. Boehmer KR, Dobler CC, Thota A, et al. Changing conversations in primary care for patients living with chronic conditions: pilot and feasibility study of the ICAN Discussion Aid. BMJ Open. 2019;9(9):e029105. Published 2019 Sep 3. doi:10.1136/bmjopen-2019-02910572

127. Fang, J.L., Collura, C.A., Johnson, R.V., Asay, G.F., Carey, W.A., Derleth, D.P., Lang, T.R., Kreofsky, B.L., & Colby, C.E. (2016) Emergency Video Telemedicine Consultation for Newborn Resuscitations: The Mayo Clinic Experience. Mayo Clin Proc. 2016 Dec;91(12):1735-1743. doi: 10.1016/j.mayocp.2016.08.006. Epub 2016 Nov 22. PMID: 27887680

128. Fang, J.L., Campbell, M.S., Weaver, A.L., Mara, K.C., Schuning, V.S., Carey, W.A., & Colby, C.E. (2018) The impact of telemedicine on the quality of newborn resuscitation: A retrospective study. Resuscitation. 2018 Apr;125:48-55. doi: 10.1016/j.resuscitation.2018.01.045. Epub 2018 Feb 3. PMID: 29408329

129. Fang, J.L., Asiedu, G.B., Harris, A.M., Carroll, K., & Colby, C.E. (2018). A Mixed-Methods Study on the Barriers and Facilitators of Telemedicine for Newborn Resuscitation. Telemed J E Health. 2018 Oct;24(10):811-817. doi: 10.1089/tmj.2017.0182. Epub 2018 Feb 8. PMID: 29420138
